# Taxonomic and functional signatures of smoking and periodontitis severity in the subgingival microbiome of older adults

**DOI:** 10.1038/s41514-025-00319-9

**Published:** 2025-12-29

**Authors:** Jale Moradi, Ellen Berggreen, Eva Gerdts, Ester Kringeland, Anne Isine Bolstad, Dagmar F. Bunæs, Randi Jacobsen Bertelsen

**Affiliations:** 1 Oral Health Center of Expertise in Western Norway, Bergen, Norway; 2 Department of Biomedicine, University of Bergen, Bergen, Norway; 3 Department of Clinical Science, University of Bergen, Bergen, Norway; 4 Department of Heart Disease, Haukeland University Hospital, Bergen, Norway; 5 Department of Clinical Dentistry, University of Bergen, Bergen, Norway

**Keywords:** Microbiology, Biomarkers, Diseases, Health care, Medical research, Risk factors

## Abstract

Periodontitis and smoking are major contributors to oral and systemic health deterioration in aging adults. This study investigated the combined effects of smoking status and periodontitis severity on the subgingival microbiome in 1107 individuals aged 69–72 using shotgun metagenomic sequencing. Smoking was linked to reduced microbial diversity, enrichment of periodontal pathogens, and depletion of health-associated commensals, while increasing periodontitis severity was associated with broader dysbiotic shifts, including enrichment of canonical pathogens. The presence of overlapping taxa suggests shared dysbiotic pathways that may accelerate disease progression in older adults. Notably, the combination of smoking and severe periodontitis was characterized by enrichment of key pathogens, such as *Tannerella forsythia*, *Fusobacterium nucleatum*, *Actinomyces israelii*, and *Mogibacterium timidum*. Although former smokers showed fewer opportunistic pathogens than current smokers, their microbiomes remained altered compared to never smokers, suggesting persistent differences potentially related to past smoking. Functional profiling revealed largely additive effects of smoking and periodontitis, with enrichment of lipopolysaccharide biosynthesis, proteolysis, and sulfur metabolism, alongside depletion of commensal biosynthetic functions. Overall, the findings highlight the persistent and additive impacts of smoking and periodontitis on the subgingival microbiome, underscoring the importance of addressing both exposures jointly in long-term oral health strategies for older adults.

## Introduction

Periodontitis—one of the six most prevalent NCDs globally—affects nearly half of the adult population and contributes to systemic inflammation, tooth loss, and impaired quality of life^1^. Disease severity ranges from mild/moderate to severe forms, with increasing severity linked to more extensive tissue destruction and higher risk of systemic complications^2,3^. Smoking, a major modifiable risk factor, further amplifies this burden and plays a key role in the onset and progression of periodontal disease^4–6^. The combined influence of these two conditions has significant implications for oral and systemic health^7,8^.

The subgingival microbiome is central to this relationship. It comprises more than 700 microbial species inhabiting teeth, gingiva, tongue, and periodontal pockets, forming the second-largest and most diverse microbial community in the human body^9,10^. Under healthy conditions, commensal microbes maintain ecological balance and host homeostasis. In contrast, environmental and behavioral factors such as smoking and chronic inflammation can disrupt this balance, leading to enrichment of opportunistic pathogens and depletion of health-associated taxa—processes that characterize periodontal dysbiosis^11,12^.

According to the World Population Prospects 2024, by the late 2070s, the global population aged 65 and older is projected to exceed 2.2 billion, driven by declining fertility rates and increasing life expectancy^13^. As this demographic segment grows, research dedicated to the health of older adults becomes increasingly urgent. Older adults represent a particularly vulnerable group for dysbiotic changes, as age-related physiological alterations, immune decline, and xerostomia—combined with long-term exposures such as smoking and chronic oral inflammation—can destabilize microbial homeostasis and promote persistent inflammation^14,15^. Understanding how these factors jointly shape the oral microbiome is therefore critical for maintaining oral and systemic health in aging populations.

Despite growing evidence linking periodontitis and smoking to oral microbiome alterations, key knowledge gaps remain regarding how these exposures jointly influence microbial composition and function in older adults. In this study, we investigate the combined effects of smoking status and periodontitis severity on the subgingival microbiome in community-dwelling adults aged 69–72 years, using shotgun metagenomic data from 1107 participants. By identifying distinct and overlapping microbial signatures associated with smoking and disease severity, we aim to determine whether their effects are additive or interacting and to provide insights into how subgingival dysbiosis contributes to oral health vulnerability in later life.

## Results

### Microbial diversity and taxonomic abundance correlated with smoking and periodontitis

**Fig. 1:**
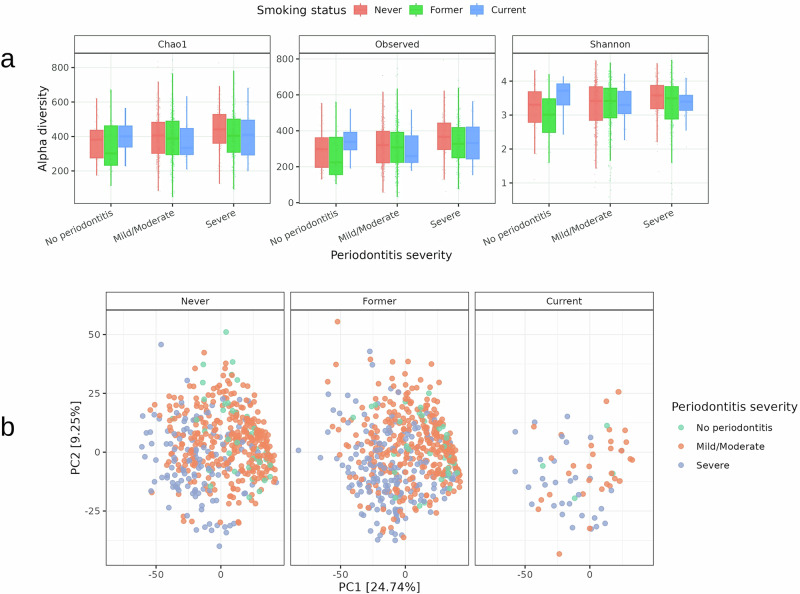
Associations of smoking status and periodontitis severity with microbial community diversity. **a** Boxplots of alpha-diversity indices (Observed, Chao1, and Shannon) across combinations of smoking status (never, former, current) and periodontitis severity (no, mild/moderate, severe). Each box shows the distribution of diversity values per subgroup. Pairwise Wilcoxon rank-sum tests were performed between groups, and *p* values were adjusted using the Benjamini–Hochberg false discovery rate (FDR). Significant contrasts (adjusted *p* < 0.05) are listed in Supplementary Table S1. **b** Principal coordinates analysis (PCoA) of Aitchison distances computed from center log-ratio (CLR)–transformed species abundances. Colors represent periodontitis severity (e.g., blue = no periodontitis, green = mild/moderate, orange = severe) within smoking categories. Two-way PERMANOVA (999 permutations) detected significant main effects of smoking status and periodontitis severity, but no significant smoking × periodontitis interaction. Homogeneity of dispersion was tested using PERMDISP; post-hoc Tukey comparisons are reported in Supplementary Table S2. CLR center log-ratio, PCoA principal coordinates analysis, PERMANOVA permutational multivariate analysis of variance, PERMDISP permutational analysis of multivariate dispersions, FDR false discovery rate.

Alpha-diversity indices (Observed, Chao1, and Shannon), calculated from species-level count data, showed distinct patterns across smoking status and periodontitis severity (Fig. 1a). In unrarefied analyses, microbial richness and diversity tended to decrease in current smokers compared to never and former smokers, while periodontitis severity was consistently associated with increased diversity, particularly in severe cases. Pairwise Wilcoxon rank-sum tests (adjusted using the Benjamini–Hochberg false discovery rate (FDR) procedure) identified several significant contrasts across smoking × periodontitis subgroups (8 significant out of 45 total comparisons; Supplementary Table S1).

To account for library size, a sensitivity analysis using rarefied count data (down-sampling to the 10th percentile of sequencing depth) was performed. In the rarefied analyses, the number of significant contrasts was markedly reduced, and the most consistent differences were observed with increasing periodontitis severity, whereas smoking-related differences were weaker and less stable (0/45 significant contrasts). These findings indicate that periodontitis severity was the primary and consistent driver of alpha-diversity, while smoking was associated with overall reductions in richness but did not consistently alter diversity patterns after accounting for sequencing depth. Importantly, no significant smoking × periodontitis interaction effect on alpha-diversity was observed after FDR correction, a pattern that mirrors the overall lack of interaction reported in the beta-diversity analyses.

To formally evaluate the combined and interactive effects of smoking and periodontitis on overall community composition, we conducted a two-way PERMANOVA on Aitchison distances. This analysis revealed significant main effects of smoking status (*F* = 5.04, *R*^2^ = 0.0087, *p* = 0.001) and periodontitis severity (*F* = 27.06, *R*^2^ = 0.0465, *p* = 0.001), no significant smoking × periodontitis interaction (*F* = 1.16, *R*^2^ = 0.0040, *q* = 0.166), indicating that the two factors act primarily in an additive rather than interactive manner. PERMDISP tests confirmed that these results were not driven by heterogeneity of dispersion, with no significant dispersion effect for smoking status (*p* = 0.283), while periodontitis severity showed significant dispersion differences (*p* = 0.001; Supplementary Table S2a–c).

Beta-diversity patterns visualized using principal coordinates analysis (PCoA) plots further supported these findings (Fig. 1b). In never and former smokers, significant differences were observed between no periodontitis and severe, no periodontitis and mild/moderate, and mild/moderate and severe groups. In current smokers, significant separation was only observed between mild/moderate and severe periodontitis, with weaker contrasts between other subgroups. Together, these results indicate that smoking reduces overall community variation and homogenizes subgingival microbiomes, while periodontitis severity remains a stronger determinant of beta-diversity.

**Fig. 2:**
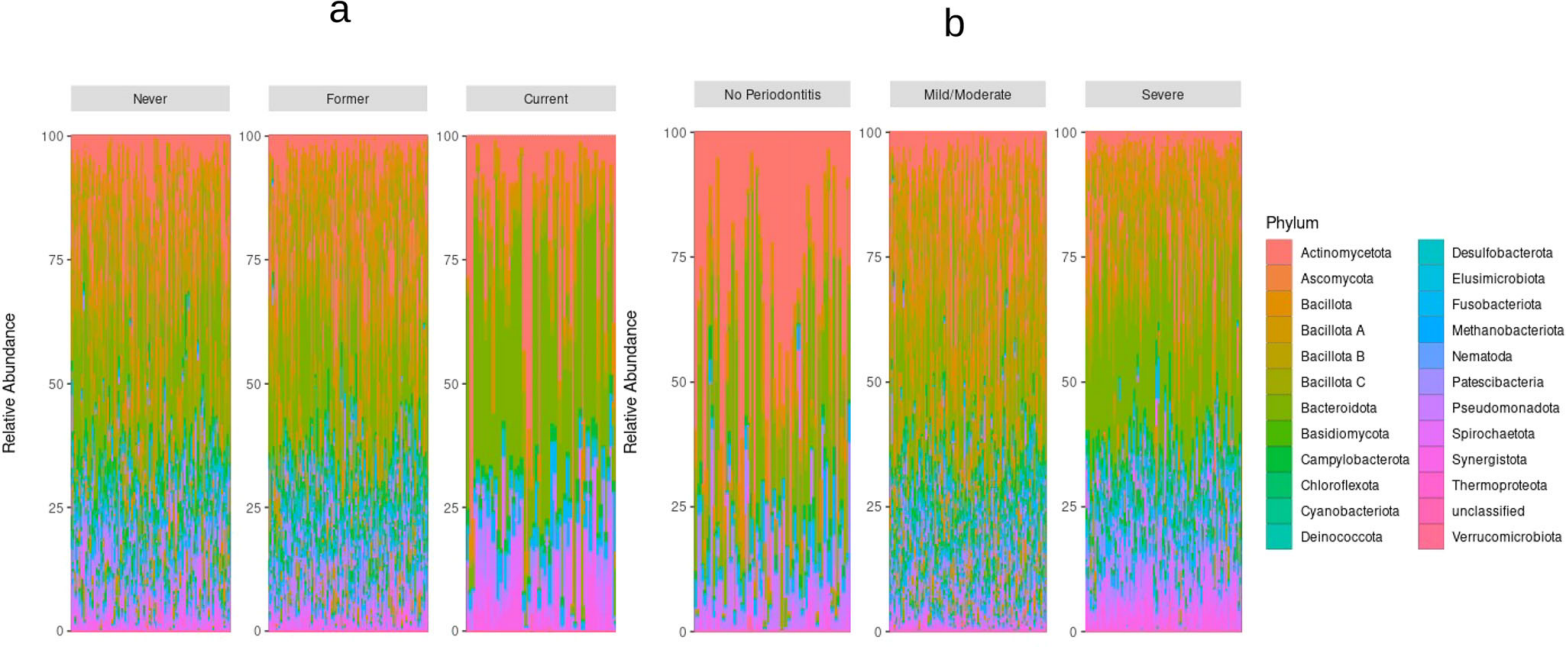
Relative abundance of microbial phyla across smoking status and periodontitis severity. **a** Stacked bar plots showing relative abundance of microbial phyla across smoking groups (never = 494, former = 545, current = 66). The most abundant phyla—Actinomycetota, Bacillota, Bacteroidota, and Pseudomonadota—were consistently higher in never and former smokers than in current smokers. **b** Stacked bar plots showing relative abundance of microbial phyla across periodontitis severity categories (no = 83, mild/moderate = 687, severe = 337). Mild/moderate periodontitis was characterized by higher relative abundance of Actinomycetota, Bacillota, and Bacteroidota, whereas severe periodontitis showed enrichment of Synergistota and depletion of commensal phyla. Only the 20 most abundant phyla are displayed; rarer phyla ( < 0.1% mean relative abundance) are grouped as “other.” Bars were normalized using total-sum scaling.

At the phylum level, Actinomycetota, Bacillota, Bacteroidota, and Pseudomonadota were the most abundant groups across smoking status and periodontitis severity (Fig. 2a, b). These phyla exhibited higher relative abundances in never and former smokers compared to current smokers, and their relative abundance was also higher in the mild/moderate periodontitis group compared to the no periodontitis and severe groups. Medium-abundance phyla such as Fusobacteriota, Patescibacteria, and Synergistota were commonly observed across both smoking status and periodontitis severity. These phyla exhibited lower abundances in current smokers compared to never and former smokers. With respect to periodontitis severity, Fusobacteriota and Patescibacteria were more abundant in the mild/moderate group, whereas Synergistota showed higher abundance in the severe periodontitis group compared to both the no periodontitis and mild/moderate groups.

### Multivariable linear model analysis of microbial taxa associated with smoking and periodontitis severity

**Fig. 3:**
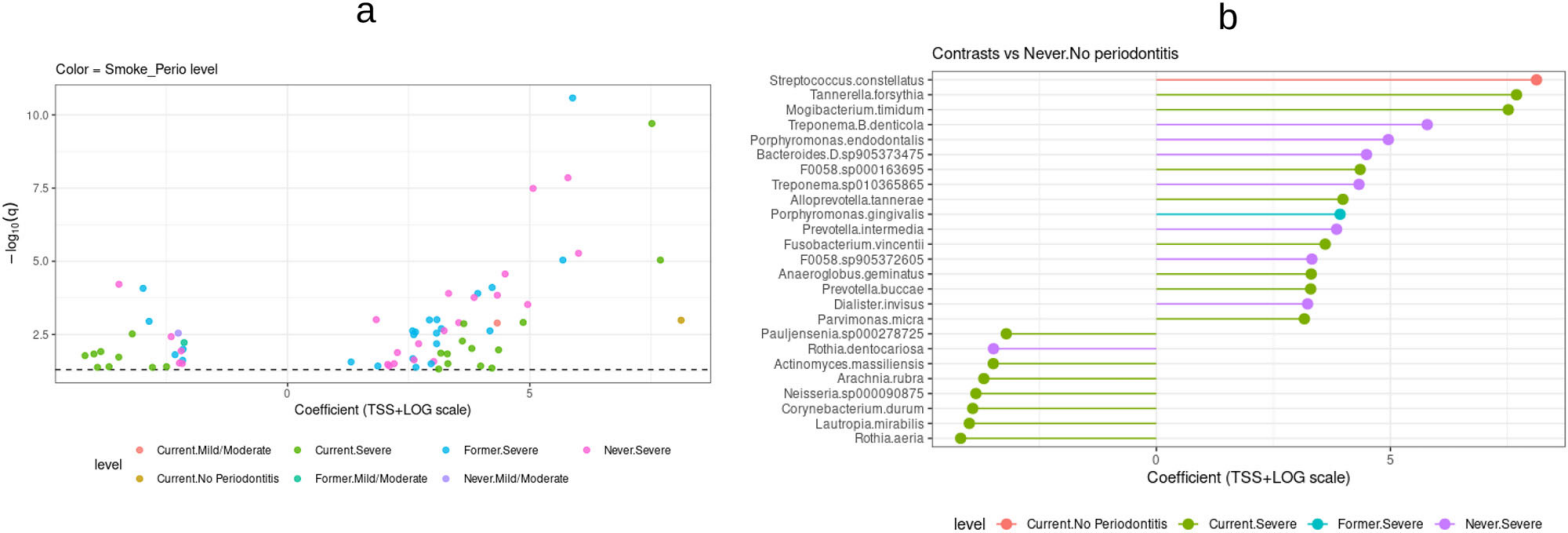
Microbial taxa associated with smoking status and periodontitis severity. **a** Volcano plot showing species-level associations (*q* < 0.05, Benjamini–Hochberg false-discovery-rate correction). Each point represents a microbial species, with the regression coefficient (effect size; TSS + LOG scale) on the *x*-axis and significance (−log_10_ q) on the *y*-axis. Points are colored according to combined smoking × periodontitis categories (amber = current/no periodontitis, dark orange = current/mild–moderate, magenta = current/severe, green = former/mild–moderate, blue = former/severe, navy = never/mild–moderate, gray = never/severe). The dashed horizontal black line marks the FDR threshold (q = 0.05). **b** Lollipop plot showing regression coefficients (TSS + LOG scale) for species significantly associated with smoking status and periodontitis severity (*q* < 0.05) in the adjusted model. Each line corresponds to one species, and colored circles indicate exposure categories (same color scheme as in (**a**). Enriched opportunistic pathogens include *F. nucleatum, T. forsythia, T. denticola, P. micra, and M. timidum*, whereas health-associated commensals such as *Rothia aeria* and *Actinomyces naeslundii* were depleted. Full results are provided in Supplementary Table S4. Together, the two panels illustrate additive, rather than interactive, effects of smoking and periodontitis severity on the subgingival microbiome, highlighting enrichment of canonical periodontal pathogens and depletion of commensal taxa across combined exposure groups. TSS total-sum scaling, FDR false discovery rate.

**Fig. 4:**
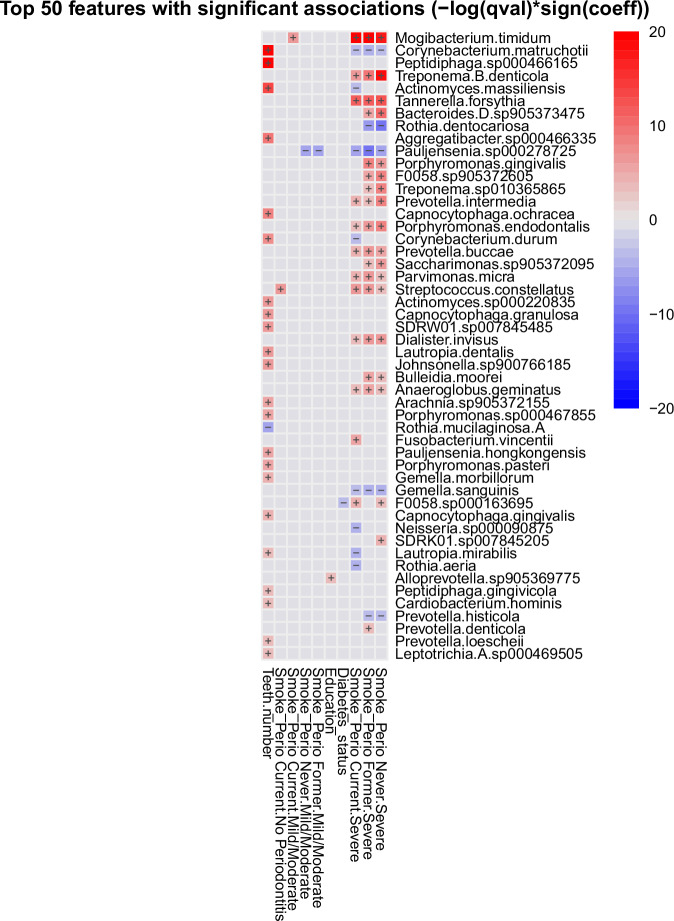
Multivariable analysis patterns associated with smoking and periodontitis severity. Heatmap showing relative effect sizes of 33 species significantly associated with smoking status and periodontitis severity (*q* < 0.05, adjusted model). Rows represent microbial species, and columns correspond to exposure categories and covariates. Warmer colors (red shades) indicate positive associations (enrichment), whereas cooler colors (blue shades) indicate negative associations (depletion). Clustering highlights enrichment of canonical periodontal pathogens (*F. nucleatum*, *T. forsythia*, *T. denticola*, *F. fastidiosum*, and *F. alocis*) and depletion of health-associated commensals (*Actinomyces* spp., *R. aeria*, and *L. mirabilis*). The complete list of significant taxa and coefficients is provided in Supplementary Table S4. q, false-discovery-rate–adjusted *p* value.

The multivariable linear model analysis using MaAsLin2 identified microbial taxa significantly associated with smoking status and periodontitis severity (*q* < 0.05; Figs. 3, 4 and Supplementary Tables S3, S4). In the unadjusted model, 154 unique taxa were associated with at least one variable. When adjusting for potential confounders, including sex, number of teeth, education, and diabetes status, a more conservative set of 72 associations corresponding to 33 unique taxa remained significant. Thus, the unadjusted analysis captured the broadest range of associations, while the adjusted analysis highlighted the most robust taxa linked to smoking and periodontitis.

In the adjusted analysis, most associations were concentrated in groups with severe periodontitis. Twenty-two taxa were linked to current smokers with severe periodontitis, 23 to former smokers with severe periodontitis, and 23 to never smokers with severe periodontitis. Only single taxa were detected for mild/moderate periodontitis in each smoking category and for current smokers without periodontitis, suggesting that the combined effect of smoking and severe disease exerts the strongest influence on the subgingival microbiome.

Taxa positively associated with smoking and periodontitis included several well-known periodontal pathogens. Enriched species were *Tannerella forsythia, Treponema denticola, Parvimonas micra, Mogibacterium timidum*, and *Streptococcus constellatus.* Additional recurrent associations were observed for *Prevotella intermedia, Prevotella buccae, Gemella sanguinis,* and *Dialister invisus*, each appearing in multiple contrasts*. Pauljensenia* sp000278725 was the most frequent, being significant in five exposure categories. These repeated signals highlight a core group of taxa strongly linked to smoking and periodontitis across groups. Conversely, taxa negatively associated with smoking and severe periodontitis included *Rothia aeria*, *Lautropia mirabilis*, and multiple *Actinomyces* spp., organisms typically associated with periodontal health. Importantly, when an explicit smoking × periodontitis interaction term was included in the model, no taxa remained significant after multiple testing correction, indicating that the observed associations represent additive rather than synergistic effects.

In the unadjusted model, a broader set of associations emerged, with 154 unique taxa significantly linked to smoking or periodontitis severity (*q* < 0.05; Supplementary Table S3). Within this, 193 associations were attributed to periodontitis severity (102 with severe periodontitis and 91 with mild/moderate periodontitis), reflecting that several taxa were linked to both severity categories. For smoking status, 24 positive associations were detected, including 20 taxa enriched in current smokers and 4 taxa enriched in former smokers. Examples included opportunistic pathogens such as *Fusobacterium nucleatum*, *T. forsythia*, *Fretibacterium fastidiosum*, *Actinomyces israelii*, and *M. timidum*. In contrast, 45 taxa were depleted in current smokers and 46 in former smokers, including *Actinomyces naeslundii*, *Actinomyces johnsonii*, *L. mirabilis*, and *R. aeria*.

Overlap analysis revealed convergence between smoking and severe periodontitis. A total of 61 taxa were shared between former smokers and severe periodontitis, with 27 showing the same direction of association (18 negative, 9 positive). Current smokers and severe periodontitis shared 66 taxa, of which 41 had concordant associations, including *F. nucleatum*, *F. fastidiosum*, *T. forsythia*, *A. israelii*, *M. timidum*, and *Filifactor alocis*. In mild/moderate disease, 37 taxa overlapped with former smokers, nearly all negatively associated with smoking but positively associated with periodontitis, and 46 overlapped with current smokers, with approximately half positive for both exposures.

Taken together, the adjusted model highlighted a robust set of 33 taxa associated with smoking and periodontitis after accounting for potential confounders, while the unadjusted model revealed a broader range of associations. Importantly, 37 taxa overlapped between the two models, including key pathogens such as *F. nucleatum*, *T. forsythia*, *M. timidum*, and *P. micra*. This overlap supports the robustness of the adjusted findings, whereas additional taxa unique to the unadjusted model likely reflect confounding influences.

### Bipartite association network analysis of microbial interactions in smoking and periodontitis severity

**Fig. 5:**
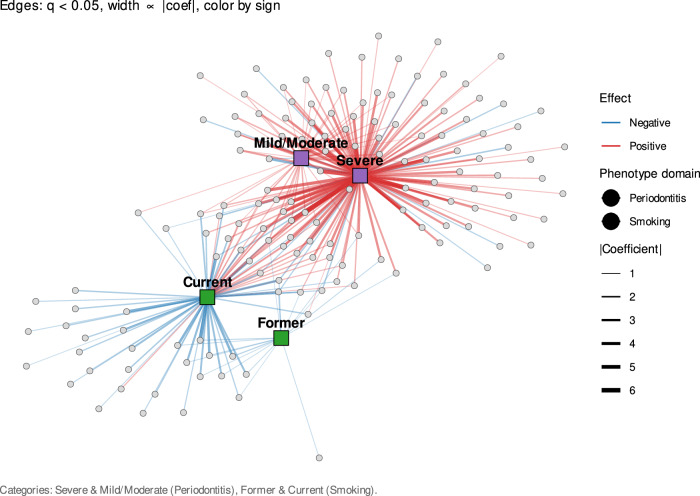
Bipartite association network of microbial taxa linked to smoking status and periodontitis severity. Network graph showing significant MaAsLin2 associations between microbial species and phenotype categories. Nodes represent microbial species (grey circles) and phenotype categories (colored squares): severe periodontitis, mild/moderate periodontitis, current smoking, and former smoking. A total of 154 microbial taxa are included in the network (see Supplementary Table S5). Node size reflects degree centrality, with larger nodes indicating taxa connected to more phenotype categories. Edges represent significant MaAsLin2 associations (*q* < 0.05), with edge width proportional to the absolute value of the regression coefficient. Edge color indicates the direction of association (red for positive associations and blue for negative associations).

The bipartite network comprised 162 nodes (158 microbial taxa and 4 phenotype categories) and 428 MaAsLin2-derived associations (*q* < 0.05) (Fig. 5 and Supplementary Table S5). Category nodes dominated the network structure, with severe periodontitis showing the highest centrality values (degree = 122, strength = 323.8, eigenvector = 1.0), followed by current smokers (degree = 65, strength = 126.8), mild/moderate periodontitis (degree = 46, strength = 61.7), and former smokers (degree = 50, strength = 40.5).

Among microbial taxa, several disease-associated species emerged as central nodes, including *M. timidum, F. fastidiosum, Treponema sp. C, Campylobacter rectus, and T. forsythia*, all of which exhibited high strength and eigenvector centrality. In contrast, health-associated species such as *R. aeria* and *L. mirabilis* remained peripheral with low connectivity and minimal bridging roles. The predominance of severe periodontitis-linked taxa in central positions underscores their ecological importance in maintaining dysbiotic network connectivity, whereas smoking cessation was associated with lower centrality and weaker microbial associations.

### Functional profiling of microbial pathways

Shotgun metagenomic functional profiling identified 142 KEGG-mapped functional features (KO-aggregated pathway/module features) significantly associated with smoking status and periodontitis severity after FDR correction (*q* < 0.05; Supplementary Table S6). These results are based on the unadjusted model and should be interpreted as exploratory, serving to complement the more conservative taxonomic associations. The majority of pathway associations reflected additive rather than interactive effects, consistent with the taxonomic findings.

Pathways positively associated with current smoking included several functions linked to lipopolysaccharide (LPS) biosynthesis, peptidoglycan turnover, and oxidative stress resistance, consistent with enrichment of Gram-negative anaerobic taxa. In contrast, pathways related to short-chain fatty acid metabolism, amino acid biosynthesis (e.g., arginine and lysine), and carbohydrate degradation were depleted in current smokers, indicating disruption of commensal metabolic activity. Former smokers displayed fewer significant associations, though persistent alterations remained in pathways related to glycan biosynthesis and xenobiotic metabolism, suggesting incomplete recovery after cessation.

With respect to periodontitis severity, severe disease was associated with enrichment of pathways involved in proteolysis, sulfur metabolism, and bacterial chemotaxis/motility, all of which are central to advanced periodontal dysbiosis. Conversely, mild/moderate periodontitis showed a smaller number of altered pathways, with depletion of biosynthetic and energy-yielding functions less pronounced than in severe cases.

**Fig. 6:**
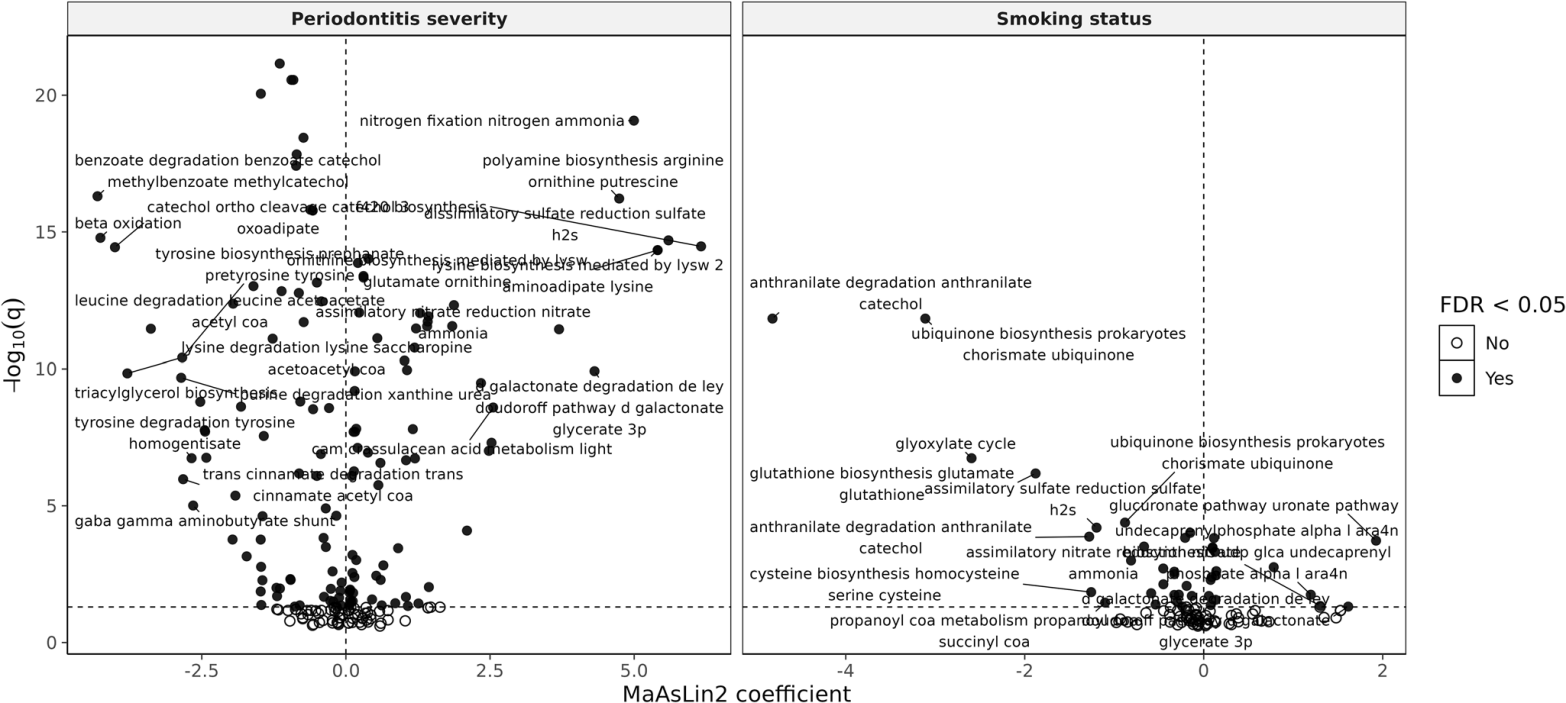
Functional pathway associations with smoking status and periodontitis severity. Volcano plots show MaAsLin2 coefficients (*x*-axis) versus –log10(*q*-values) (*y*-axis) for KEGG Orthology pathways. Pathways passing FDR < 0.05 are highlighted. Left: periodontitis severity – enrichment of proteolysis, sulfur metabolism, and chemotaxis/motility pathways, with depletion of biosynthetic and energy-yielding functions. Right: smoking status—enrichment of LPS biosynthesis, peptidoglycan metabolism, and oxidative stress resistance, alongside depletion of amino acid biosynthesis and carbohydrate degradation. Results reflect the unadjusted model and are exploratory (*q* < 0.05; Supplementary Table S6). Expanded visualizations are shown in Supplementary Figs. S1 and S2.

A volcano plot summarizing these associations highlights distinct functional shifts linked to smoking and periodontitis severity (Fig. 6). Expanded visualizations, including lollipop and heatmap representations, are provided in the Supplementary Information (Supplementary Figs. S1 and S2). Together, these results indicate that both smoking and periodontitis alter the functional capacity of the subgingival microbiome, primarily through additive enrichment of pro-inflammatory and tissue-destructive pathways alongside loss of commensal metabolic functions.

## Discussion

This study provides new insights into how smoking status and periodontitis severity jointly influence the subgingival microbiome in older adults, a population particularly vulnerable to oral and systemic health challenges. We demonstrate that smoking and periodontitis exert distinct but largely additive, rather than interacting, effects on the microbiome. Taxonomic associations were derived from models adjusted for key covariates, providing robust estimates of microbial shifts linked to smoking and periodontitis. In parallel, functional profiling was performed using unadjusted models to maximize detection power, given that pathway-level signals are typically weaker. By integrating these two complementary approaches, we show that compositional changes in key taxa translate directly into functional reprogramming of the microbiome.

The adjusted taxonomic analysis identified a core set of microbial associations concentrated in severe periodontitis, including enrichment of classical periodontal pathogens such as *F. nucleatum, T. forsythia, T. denticola*, and *P. micra*, alongside consistent depletion of health-associated taxa such as *Actinomyces*, *R*. *aeria,* and *L*. *mirabilis*^16,17^. These taxonomic shifts were mirrored by functional enrichment of LPS biosynthesis, proteolysis, and oxidative stress resistance, while depletion of commensal species corresponded with loss of amino acid and carbohydrate metabolism^18,19^. Together, this indicates that disease severity amplifies the impact of smoking by simultaneously eroding health-associated taxa and enhancing community-level inflammatory capacity.

Our analysis reveals that in never smokers, microbial diversity (alpha diversity) increases with periodontitis severity, reflecting ecological shifts typical of disease progression^20^. In contrast, current smokers show reduced alpha diversity regardless of disease severity, indicating patterns consistent with smoking-associated dysbiosis that may obscure microbial changes usually observed during periodontitis^21,22^. However, these alpha-diversity differences were less robust after rarefaction, indicating that periodontitis severity was the more consistent driver, whereas smoking effects were weaker and less stable. Such results align with previous reports suggesting enrichment of pathogen-dominated biofilms in smokers early in disease development, potentially contributing to loss of ecological resilience in later stages^23^. This homogenizing effect of smoking was further supported by functional analysis, where reduced diversity of commensals coincided with depletion of biosynthetic pathways critical for maintaining ecological balance.

Beta diversity analysis further supports this: microbial community differences between disease stages are clear in never and former smokers but become blurred in current smokers, suggesting smoking-associated homogenization of the subgingival microbiome^24^. Two-way PERMANOVA confirmed significant main effects of smoking and periodontitis, but no significant smoking × periodontitis interaction, supporting the conclusion that their influences act independently rather than synergistically. Notably, reductions in Actinomycetota and other commensal phyla among current smokers concur with evidence that cigarette smoke is associated with depletion of beneficial bacteria and relative enrichment of periodontal pathogens^25,26^. Functionally, this corresponded to loss of pathways supporting commensal activity (e.g., amino acid biosynthesis) and enrichment of xenobiotic degradation and stress response pathways, consistent with microbial adaptation to smoke-derived compounds^27^.

Using multivariable modeling, we identified 33 robust taxa in adjusted models linked to smoking and periodontitis, highlighting a complex microbial landscape shaped by these factors. Smoking was positively associated with a smaller set of taxa but negatively with a broader range, indicating a reduction in beneficial or neutral microbes alongside enrichment of opportunistic pathogens^28^. Particularly, Gram-positive species within the *Actinomyces* genus decreased with smoking, consistent with prior findings^25^. This depletion corresponded with reduced carbohydrate and amino acid metabolism, suggesting that loss of these early colonizers weakens the metabolic foundation for microbial homeostasis. Conversely, smoking increased Gram-negative pathogens, including *F. nucleatum*, *T. forsythia*, and various *Prevotella* species—key periodontitis-associated taxa^29,30^. Their enrichment coincided with functional upregulation of LPS biosynthesis and proteolysis, mechanisms central to immune activation and tissue destruction.

These results support the hypothesis that smoking was associated with a higher abundance of anaerobic Gram-negative bacteria adapted to the low-oxygen periodontal pocket environments^21,26^. Distinct microbial patterns between current and former smokers suggest that smoking was associated with long-lasting microbiome alterations persisting into older age^31,32^. While former smokers’ overall community composition more closely resembles never smokers than of current smokers, significant differences remain, indicating persistent impacts of past smoking on oral microbiome and disease risk^33,34^.

Regarding periodontitis severity, positive associations with canonical periodontal pathogens from the Red and Orange complexes—*T. forsythia, T. denticola, F. nucleatum*, and *Prevotella nigrescens*—were confirmed^16,35^. These taxa have been implicated in dysbiosis, inflammation, and tissue destruction, especially in the context of aging and immune decline^16,17^. In contrast, taxa such as *Actinomyces, L. mirabilis*, and *R. aeria* showed inverse relationships, underscoring disruption of commensal balance in severe disease^17^. At the functional level, severe periodontitis was associated with upregulation of sulfur metabolism and bacterial chemotaxis/motility, supporting the ecological shift toward proteolytic, motile communities that dominate advanced periodontal niches^36,37^.

Rather than unique or synergistic interactions, the combined influences of smoking and periodontitis severity appear primarily additive, producing overlapping patterns of dysbiosis that may exacerbate disease progression. Shared microbial taxa abundant in individuals with both smoking and severe periodontitis—including *T. forsythia, F. nucleatum, A. israelii*, and *M. timidum*—likely represent overlapping enrichment patterns rather than distinct combined effects^38,39^. The functional parallels—LPS biosynthesis, proteolysis, and oxidative stress resistance—suggest that these taxa act as hubs linking structural dysbiosis with pro-inflammatory metabolic output.

In the context of severe periodontitis and smoking status, current smokers displayed a higher number of significantly associated taxa, many of which were opportunistic pathogens such as *T. forsythia and F. nucleatum* ^30,40^. In contrast, former smokers showed fewer significant associations with severe periodontitis, and both the number and direction of associations differed. For example, *T. forsythia* was no longer detected, and the association with *F. nucleatum* shifted compared to that in current smokers^22,34^. Functionally, this attenuation in former smokers corresponded with loss of strong LPS enrichment, suggesting partial recovery of immune-related pathways. These findings suggest that smoking cessation is associated with partial microbiome recovery and reduced prevalence of opportunistic pathogens, though some effects of past smoking persist. Importantly, the relatively small number of current smokers in this study (*n* = 66) limits statistical power for subgroup analyses and may have constrained the ability to detect more subtle associations. This lingering dysbiosis could contribute to the continued risk of periodontal breakdown in former smokers when compared to never smokers.

For mild to moderate periodontitis, a similar but less pronounced pattern was observed. Both current and former smokers had fewer significant taxa associated with disease compared to those with severe periodontitis, highlighting the stronger influence of disease severity over smoking status alone^41^. In milder forms of periodontitis, the microbial imbalance appears to be less extensive, which may explain the reduced number of associated taxa. Correspondingly, functional alterations were less pronounced, with only modest depletion of biosynthetic pathways, suggesting that early-stage dysbiosis may retain partial metabolic resilience.

Bipartite association network analysis identified central taxa—such as *M. timidum, F. fastidiosum, Treponema* sp. C*, C. rectus, and T. forsythia*—which appeared to sustain dysbiotic network connectivity^37^. Reduced network connectivity in former smokers suggests partial recovery of microbial community structure, though residual disruption remains. The network structure in current smokers reflected destabilization and reduced modularity, consistent with smoking’s additive role in weakening microbial resilience rather than producing significant interaction effects. These network-level disruptions align with functional findings of reduced biosynthetic capacity and enhanced stress resistance, suggesting that structural fragility and metabolic reprogramming reinforce each other.

In summary, this study highlights the intertwined roles of smoking and periodontitis severity in shaping the aging subgingival microbiome. Taxonomic changes identified in adjusted models were complemented by functional shifts from unadjusted exploratory analysis, both pointing to additive enrichment of pathogens and loss of commensals. Smoking was associated with enrichment of opportunistic pathogen hubs and reduced community stability, while disease progression was associated with patterns that paralleled those observed in smokers. By linking compositional and functional findings, we demonstrate that dysbiosis manifests not only as structural changes but also as altered metabolic capacity, linked to inflammation and tissue destruction. Although cessation was associated with partial recovery, residual dysbiosis underscores the need for early preventive strategies. Our results suggest that these influences are largely additive rather than interactive, reinforcing the importance of addressing both risk factors concurrently. Therapeutic approaches combining periodontal care, smoking cessation, and targeted microbiome modulation may help preserve oral and systemic health in older adults.

## Methods

### Study design and population

The present study is part of HUSK-T (HUSK Dental Health), an oral health sub-study of the Hordaland Health Study (HUSK), a population-based study conducted in Western Norway. It included 1107 participants born in 1950–1951 who had taken part in all three waves of HUSK (HUSK 1–3) and subsequently underwent comprehensive dental and periodontal examinations. Detailed oral examinations were performed by trained dentists, including X-rays (2BW and OPG), mucosal exams, dental status recording, caries assessments, and periodontal measurements. Periodontal probing depth was measured at six sites per tooth to evaluate pocket depth and bleeding on probing (BOP).

Smoking status was determined using a standardized questionnaire and classified into three categories as never smokers (*n* = 494), former smokers (*n* = 545), and current smokers (*n* = 66). In total, smoking status was available for 1105 of the 1107 participants; two participants had missing data and were excluded only from analyses requiring smoking information. After harmonization of sequencing data with metadata, 1104 participants were included in the multivariable MaAsLin2 analyses.

Periodontitis severity was assessed according to the 2018 EFP/AAP classification framework and categorized as: no periodontitis (*n* = 83), mild/moderate periodontitis (Stages I–II; *n* = 687), and severe periodontitis (Stages III–IV; *n* = 337)^2,3^. Sex distribution included 603 females and 504 males.

This study builds on our earlier work in the HUSK-T cohort^42^, which examined intra-individual differences in microbial composition and functional profiles between shallow and deep periodontal pockets. The present manuscript focuses on participant-level associations with smoking status and periodontitis severity, extending the scope to assess their joint and additive effects on the subgingival microbiome. Information on the prevalence and classification of periodontitis in this population has been reported previously^43^.

The HUSK-T registry (No. 279585) was approved by the Norwegian Centre for Research Data (NSD) and by the data protection officer in Western Norway County. This study was approved by the Norwegian Regional Committee for Medical and Health Research Ethics (REK No. 263006). Written informed consent was obtained from all participants. This study was conducted in accordance with the principles of the Declaration of Helsinki.

### Sampling strategy and unit of analysis

Subgingival plaque was collected from two periodontal pocket sites on distinct teeth, primarily in the upper jaw, using sterile paper points (COLTENE, USA). The two-site samples were pooled into a single tube, yielding one metagenome per participant. In the present study, when both shallow pockets (≤4 mm without BOP) and deep pockets (≥5 mm with BOP) were present in an individual, the deep pocket sample was prioritized; if no deep pockets were present, a shallow site was included. All participants fasted for two hours prior to sample collection. In total, 1107 subgingival plaque samples were collected and stored at −80 °C until further processing. Consequently, the unit of analysis in this study was the participant, and all downstream analyses were performed at the participant level.

### Shotgun metagenomic sequencing and processing

DNA was extracted from subgingival plaque samples using the NucleoSpin Soil 96 kit (Macherey-Nagel, Germany). Sequencing libraries were prepared and processed at Clinical Microbiomics A/S (Copenhagen, Denmark), and shotgun metagenomic sequencing was performed on an Illumina NovaSeq 6000 platform, generating 2 × 150 bp paired-end reads with an average of ~33.6 million read pairs per sample. To monitor potential contamination, blank paper point controls were systematically collected (one for every 20 participants) and processed in parallel with biological samples.

Raw reads underwent quality control to remove adapter sequences and low-quality bases (Phred score <30) using AdapterRemoval v2.3.1. Host contamination was excluded by discarding read pairs mapping to the human genome (GRCh38) with Bowtie2 v2.4.4 in local mode. Read pairs were retained if both reads passed filtering and had a length of at least 100 bp. The resulting high-quality non-host reads were mapped to the Clinical Microbiomics HMR05 gene catalog using BWA mem v0.7.17. Reads were classified as uniquely mapped (≥95% identity, ≥100 bp, MAPQ ≥ 20), multi-mapped, or unmapped, and only uniquely mapped reads were used for species abundance profiling.

Species abundances were estimated using a negative binomial distribution model that accounted for effective gene length and read dispersion, and normalized such that the total abundance per sample summed to 100%. Taxonomic classification was performed according to the Genome Taxonomy Database (GTDB, release r214). Functional potential was inferred by mapping genes to orthologous groups using EggNOG-mapper v2.1.7 and assigning functional annotations to the KEGG Orthology (KO) database, which enabled the construction of functional gene sets and modules.

To ensure reproducibility, per-sample quality control metrics including read depth, host-read removal, and percentage of classified reads are provided (Supplementary Table S7). Full details of the sequencing and taxonomic profiling workflow have been described previously^42^.

### Diversity and abundance analyses

Alpha-diversity of the microbiome was represented by the Observed, Chao1, and Shannon indices, calculated from species-level count data. The analysis included groups defined by smoking status (never, former, and current) and periodontitis severity (no periodontitis, mild/moderate, and severe). Statistical comparisons of alpha-diversity indices were performed between periodontitis severity levels within each smoking category using pairwise Wilcoxon rank-sum tests, with *p* values adjusted for multiple testing using the Benjamini–Hochberg FDR, and significance defined as *q* < 0.05.

Beta-diversity was assessed using species-level Aitchison distances, computed from center log-ratio transformed abundance data. The resulting distance matrices were visualized using PCoA. To formally evaluate the effects of smoking, periodontitis severity, and their interaction, we conducted a two-way PERMANOVA (adonis2, 999 permutations) with smoking status, periodontitis severity, and the interaction term (smoking × periodontitis) as predictors. To ensure that significant PERMANOVA results were not driven by differences in dispersion, we also performed PERMDISP (betadisper) tests of homogeneity of multivariate dispersions, followed by Tukey post-hoc comparisons where relevant. For both two-way and pairwise PERMANOVA analyses, *p* values were adjusted using the Benjamini–Hochberg FDR method, with *q* < 0.05 considered significant.

Taxonomic composition was analyzed at multiple taxonomic levels, including phylum, class, order, family, genus, and species. Relative abundances of taxa within each group were compared and visualized using box plots to highlight differences between groups. Additionally, the phyloseq R package (v1.44.0) was used for taxonomic handling and visualization. To formally test differences at the community level, we also applied the Human Microbiome Project package (v2.0), which uses a Dirichlet–multinomial framework to test for differences in microbial composition between groups. This combined multivariate approach enabled the identification of variations in overall composition and highlighted significant shifts in microbial structure across sample groups. All statistical analyses were performed in R (v4.3.2). To ensure reproducibility, random number generation was seeded (set.seed(1234)).

### Multivariate linear regression analysis

Multivariate linear regression models were applied in R using the MaAsLin2 package from the bioBakery suite in R/Bioconductor^44^ to assess associations between microbial taxa abundance and smoking/periodontitis exposures. Taxa relative abundances were treated as dependent variables. Two models were fit. The first was an unadjusted model including smoking status, periodontitis severity, and their interaction as fixed effects. The second, considered the primary model, was a confounder-adjusted model in which smoking and periodontitis were encoded as a combined exposure factor (smoke_perio), while additionally adjusting for sex, number of teeth, education, and diabetes status. Reference categories were never smokers for smoking status, no periodontitis for periodontitis severity, never.no periodontitis for the combined exposure, female for sex, primary for education, and no diabetes for diabetes status. Tooth number was modeled as a continuous covariate.

Taxa abundances were normalized using total sum scaling (TSS) and log-transformed. Regression coefficients are reported as MaAsLin2 effect estimates on the TSS + LOG scale rather than as fold-changes. Multiple testing correction was applied using the Benjamini–Hochberg procedure, and *q* < 0.05 was considered statistically significant. As expected, the explicit smoking × periodontitis interaction term did not yield significant associations after multiple testing correction.

### Co-occurrence network analysis

A bipartite network analysis was performed to visualize associations between significant microbial taxa and metadata categories (smoking status and periodontitis severity) identified in the MaAsLin2 model. Microbial taxa and phenotype categories (severe, mild/moderate, former, and current) were represented as nodes, with edges denoting significant associations (*q* < 0.05). Edge weights reflected MaAsLin2 effect sizes (regression coefficients). Network centrality measures (degree, strength, betweenness, and eigenvector centrality) were calculated to identify hub and influential nodes. This analysis provided complementary insight into the ecological role of taxa across smoking and periodontitis groups.

### Functional profiling of microbial communities

Functional annotation was performed using EggNOG-mapper v2.1.7, which assigned genes to orthologous groups and KEGG Orthology (KO) identifiers. Functional abundances were aggregated at the KEGG pathway level. To assess associations between functional pathways, smoking status, and periodontitis severity, we applied MaAsLin2 models without adjustment for additional covariates. This unadjusted approach was chosen to maximize detection power, as pathway-level signals are typically weaker and more diffuse than taxonomic associations. Relative functional abundances were normalized by total sum scaling and log-transformed prior to modeling. Multiple testing correction was applied using the Benjamini–Hochberg FDR method, with *q* < 0.05 considered significant. As with the taxonomic analyses, the smoking × periodontitis interaction term did not yield significant associations after multiple testing correction. Visualization of significant pathways was performed using ggplot2 (lollipop and volcano plots) and pheatmap (clustering heatmaps).

## Supplementary information

Supplementary information

## Data Availability

All data generated or analyzed during this study are included in this published article and its supplementary information files. The raw metagenomic sequencing data supporting the findings of this study have been deposited in the European Nucleotide Archive (ENA) under the accession number PRJEB87865.

## References

[1] Wu, L. et al. Burden of severe periodontitis: new insights based on a systematic analysis from the Global Burden of Disease Study 2021. *BMC Oral. Health* **25**, 861 (2025). 40450275 10.1186/s12903-025-06271-0 PMC12125741

[2] Papapanou, P. N. et al. Periodontitis: consensus report of workgroup 2 of the 2017 world workshop on the classification of periodontal and peri-implant diseases and conditions. *J. Clin. Periodontol.* **45**, S162–S170 (2018). 29926490 10.1111/jcpe.12946

[3] Tonetti, M. S., Greenwell, H. & Kornman, K. S. Staging and grading of periodontitis: framework and proposal of a new classification and case definition. *J. Clin. Periodontol.* **45**, S149–S161 (2018). 29926495 10.1111/jcpe.12945

[4] Tamashiro, R. et al. Smoking-induced subgingival dysbiosis precedes clinical signs of periodontal disease. *Sci. Rep.* **13**, 3755 (2023). 36882425 10.1038/s41598-023-30203-z PMC9992395

[5] Prince, Y. et al. The effect of cigarette smoking on the oral microbiota in a South African population using subgingival plaque samples. *Heliyon* **10**, e31559 (2024). 38831830 10.1016/j.heliyon.2024.e31559 PMC11145493

[6] Jia, Y.-J. et al. Association between oral microbiota and cigarette smoking in the Chinese population. *Front. Cell. Infect. Microbiol.* **11**, 658203 (2021). 34123872 10.3389/fcimb.2021.658203 PMC8195269

[7] Ebersole, J. L., Al-Sabbagh, M., Gonzalez, O. A. & Dawson, D. R. III Ageing effects on humoral immune responses in chronic periodontitis. *J. Clin. Periodontol.* **45**, 680–692 (2018). 29476652 10.1111/jcpe.12881 PMC5992058

[8] Mason, M. R. et al. The subgingival microbiome of clinically healthy current and never smokers. *ISME J.* **9**, 268–272 (2015). 25012901 10.1038/ismej.2014.114 PMC4274424

[9] Peng, X. et al. Oral microbiota in human systematic diseases. *Int. J. Oral. Sci.* **14**, 1–11 (2022). 35236828 10.1038/s41368-022-00163-7 PMC8891310

[10] Sarafidou, K., Alexakou, E., Talioti, E., Bakopoulou, A. & Anastassiadou, V. The oral microbiome in older adults –a state-of-the-art review. *Arch. Gerontol. Geriatr.* **1**, 100061 (2024).

[11] Georges, F. M., Do, N. T. & Seleem, D. Oral dysbiosis and systemic diseases. *Front. Dent. Med.* **3**, 995423 (2022).

[12] Pisano, M. et al. The interaction between the oral microbiome and systemic diseases: a narrative review. *Microbiol. Res.* **14**, 1862–1878 (2023).

[13] United Nations Department of Economic and Social Affairs, Population Division*.* In *World Population Prospects 2024: Data Sources* (United Nations, 2024).

[14] Mosaddad, S. A., Mahootchi, P., Safari, S., Rahimi, H. & Aghili, S. S. Interactions between systemic diseases and oral microbiota shifts in the aging community: a narrative review. *J. Basic Microbiol.* **63**, 831–854 (2023). 37173818 10.1002/jobm.202300141

[15] Lipsky, M. S., Singh, T., Zakeri, G. & Hung, M. Oral health and older adults: a narrative review. *Dent. J.* **12**, 30 (2024). 10.3390/dj12020030 PMC10887726 38392234

[16] Plachokova, A. S., Andreu-S nchez, S., Noz, M. P., Fu, J. & Riksen, N. P. Oral microbiome in relation to periodontitis severity and systemic inflammation. *Int. J. Mol. Sci.* **22**, 5876 (2021). 34070915 10.3390/ijms22115876 PMC8199296

[17] Veras, E. L. et al. Newly identified pathogens in periodontitis: evidence from an association and an elimination study. *J. Oral. Microbiol.* **15**, 2213111 (2023). 37261036 10.1080/20002297.2023.2213111 PMC10228317

[18] Lamont, R. J. & Hajishengallis, G. Polymicrobial synergy and dysbiosis in inflammatory disease. *Trends Mol. Med.* **21**, 172–183 (2015). 25498392 10.1016/j.molmed.2014.11.004 PMC4352384

[19] Curtis, M. A., Diaz, P. I. & Van Dyke, T. E. The role of the microbiota in periodontal disease. *Periodontol 2000* **83**, 14–25 (2020). 32385883 10.1111/prd.12296

[20] Lafaurie, G. I. et al. Differences in the subgingival microbiome according to stage of periodontitis: a comparison of two geographic regions. *PLoS ONE* **17**, e0273523 (2022). 35998186 10.1371/journal.pone.0273523 PMC9398029

[21] Hanioka, T. et al. Smoking and periodontal microorganisms. *Jpn. Dent. Sci. Rev.* **55**, 88–94 (2019). 31049117 10.1016/j.jdsr.2019.03.002 PMC6484221

[22] Al Kawas, S. et al. The impact of smoking different tobacco types on the subgingival microbiome and periodontal health: a pilot study. *Sci. Rep.* **11**, 1113 (2021). 33441919 10.1038/s41598-020-80937-3 PMC7806658

[23] Jiang, Y., Zhou, X., Cheng, L. & Li, M. The impact of smoking on subgingival microflora: from periodontal health to disease. *Front. Microbiol.* **11**, 66 (2020). 32063898 10.3389/fmicb.2020.00066 PMC7000377

[24] Huang, Q. et al. Association of cigarette smoking with oral bacterial microbiota and cardiometabolic health in Chinese adults. *BMC Microbiol.* **23**, 346 (2023). 37978427 10.1186/s12866-023-03061-y PMC10655299

[25] Chattopadhyay, S. et al. Oral microbiome dysbiosis among cigarette smokers and smokeless tobacco users compared to non-users. *Sci. Rep.* **14**, 10394 (2024). 38710815 10.1038/s41598-024-60730-2 PMC11074290

[26] Cicchinelli, S. et al. The impact of smoking on microbiota: a narrative review. *Biomedicines* **11**, 1144 (2023). 37189762 10.3390/biomedicines11041144 PMC10135766

[27] Bagaitkar, J., Demuth, D. R. & Scott, D. A. Tobacco use increases susceptibility to bacterial infection. *Tob. Induc. Dis.* **4**, 12 (2008). 19094204 10.1186/1617-9625-4-12 PMC2628337

[28] Rosier, B. T., Moya-Gonzalvez, E. M., Corell-Escuin, P. & Mira, A. Isolation and characterization of nitrate-reducing bacteria as potential probiotics for oral and systemic health. *Front. Microbiol.* **11**, 555465 (2020). 33042063 10.3389/fmicb.2020.555465 PMC7522554

[29] Yang, Y. et al. Cigarette smoking and oral microbiota in low-income and African-American populations. *J. Epidemiol. Community Health* **73**, 1108–1115 (2019). 31563898 10.1136/jech-2019-212474 PMC6913090

[30] Shchipkova, A. Y., Nagaraja, H. N. & Kumar, P. S. Subgingival microbial profiles of smokers with periodontitis. *J. Dent. Res.* **89**, 1247–1253 (2010). 20739702 10.1177/0022034510377203 PMC3318026

[31] Antonello, G. et al. Smoking and salivary microbiota: a cross-sectional analysis of an Italian alpine population. *Sci. Rep.* **13**, 18904 (2023). 37919319 10.1038/s41598-023-42474-7 PMC10622503

[32] Fahey, M. C. et al. Expectations and preferences for digital cessation treatment: multimethods study among older adults who smoke cigarettes. *J. Med. Internet Res.* **26**, e52919 (2024). 39196628 10.2196/52919 PMC11391153

[33] Al Bataineh, M. T. et al. Revealing oral microbiota composition and functionality associated with heavy cigarette smoking. *J. Transl. Med.* **18**, 421 (2020). 33167991 10.1186/s12967-020-02579-3 PMC7653996

[34] Delima, S. L., McBride, R. K., Preshaw, P. M., Heasman, P. A. & Kumar, P. S. Response of subgingival bacteria to smoking cessation. *J. Clin. Microbiol.* **48**, 2344–2349 (2010). 20410352 10.1128/JCM.01821-09 PMC2897479

[35] Tsai, C.-Y. et al. Subgingival microbiota in individuals with severe chronic periodontitis. *J. Microbiol. Immunol. Infect.* **51**, 226–234 (2018). 27262209 10.1016/j.jmii.2016.04.007

[36] Olsen, I. & Yilmaz, Ö Modulation of inflammasome activity by Porphyromonas gingivalis in periodontitis and associated systemic diseases. *J. Oral. Microbiol.* **8**, 30385 (2016). 26850450 10.3402/jom.v8.30385 PMC4744328

[37] Ovsepian, A., Kardaras, F. S., Skoulakis, A. & Hatzigeorgiou, A. G. Microbial signatures in human periodontal disease: a metatranscriptome meta-analysis. *Front. Microbiol.* **15**, 1383404 (2024). 38659984 10.3389/fmicb.2024.1383404 PMC11041396

[38] Kunath, B. J., De Rudder, C., Laczny, C. C., Letellier, E. & Wilmes, P. The oral–gut microbiome axis in health and disease. *Nat. Rev. Microbiol*. 10.1038/s41579-024-01075-5 (2024). 10.1038/s41579-024-01075-5 39039286

[39] Baker, J. L., Mark Welch, J. L., Kauffman, K. M., McLean, J. S. & He, X. The oral microbiome: diversity, biogeography and human health. *Nat. Rev. Microbiol.* **22**, 89–104 (2024). 37700024 10.1038/s41579-023-00963-6 PMC11084736

[40] Alwithanani, N. Periodontal disease and smoking: systematic review. *J. Pharm. Bioallied Sci.* **15**, S64–S71 (2023). 37654319 10.4103/jpbs.jpbs_516_22 PMC10466628

[41] Papapanou, P. N. et al. Subgingival microbiome and clinical periodontal status in an elderly cohort: the WHICAP ancillary study of oral health. *J. Periodontol.* **91**, S56–S67 (2020). 32533776 10.1002/JPER.20-0194 PMC8324315

[42] Moradi, J., Berggreen, E., Bunæs, D. F., Bolstad, A. I. & Bertelsen, R. J. Microbiome composition and metabolic pathways in shallow and deep periodontal pockets. *Sci. Rep.* **15**, 12926 (2025). 40234709 10.1038/s41598-025-97531-0 PMC12000285

[43] Elabdeen, H. R. Z. et al. Prevalence of periodontitis in a 70-year-old population in western Norway according to the ACES 2018 classification framework: a cross-sectional study. *J. Clin. Periodontol*. 10.1111/jcpe.14128 (2025). 10.1111/jcpe.14128 PMC12003053 39895368

[44] Mallick, H. et al. Multivariable association discovery in population-scale meta-omics studies. *PLoS Comput. Biol.* **17**, e1009442 (2021). 34784344 10.1371/journal.pcbi.1009442 PMC8714082

