# Supplementary material for: Taxonomic and functional signatures of smoking and periodontitis severity in the subgingival microbiome of older adults
Source: NPJ Aging. 2025 Dec 29;12(1):20. doi: 10.1038/s41514-025-00319-9 (PMC12855792; doi:10.1038/s41514-025-00319-9)
Supplement: Supplementary file 1 — Supplementary information [file 41514_2025_319_MOESM1_ESM.pdf]

Supplementary Information

Supplementary Table S1.
This table reports pairwise Wilcoxon rank-sum test results for alpha-diversity indices (Observed, Chao1, Shannon) across subgroups defined by smoking status and periodontitis severity. P-values were adjusted for multiple testing using the Benjamini–Hochberg false discovery rate (FDR) method, and significant contrasts (adjusted $p < 0.05$) are highlighted.

Supplementary Table S2.
This table summarizes multivariate analyses of community structure. (a) Two-way PERMANOVA on Aitchison distances (Euclidean distances of CLR-transformed abundances) tested the effects of smoking status, periodontitis severity, and their interaction (Smoking × Periodontitis), with F-values, R^2, and permutation-based p-values (999 permutations) reported. (b) PERMDISP results show tests of homogeneity of multivariate dispersions for smoking status and periodontitis severity. (c) Tukey post-hoc pairwise comparisons of dispersions between subgroups are provided.

Supplementary Table S3.
This table lists microbial taxa significantly associated with smoking status and periodontitis severity in the unadjusted MaAsLin2 models ($q < 0.05$). Results include regression coefficients, standard errors, and FDR-adjusted q-values for each taxon across smoking categories (never, former, current) and periodontitis severity levels (no, mild/moderate, severe). In total, 154 unique taxa were detected, reflecting the broader range of associations identified without covariate adjustment.

Supplementary Table S4.
This table presents microbial taxa significantly associated with smoking status and periodontitis severity in the adjusted MaAsLin2 models ($q < 0.05$), with adjustment for sex, number of teeth, education, and diabetes status. Regression coefficients, standard errors, and q-values are reported. A more conservative set of 33 taxa was identified, including enrichment of canonical periodontal pathogens such as *Fusobacterium nucleatum*, *Tannerella forsythia*, *Treponema denticola*, and *Parvimonas micra*, alongside depletion of health-associated commensals including *Actinomyces* spp., *Rothia aeria*, and *Lautropia mirabilis*.

Supplementary Table S5.
This table provides node-level network properties of microbial taxa associated with smoking and periodontitis. Metrics include degree centrality, closeness centrality, betweenness centrality, and categorical assignment (associated with smoking, former smoking, mild/moderate periodontitis, or severe periodontitis). The network comprised 154 taxa and 428 positive edges. High-degree taxa such as *F. nucleatum, T. forsythia,* and *M. timidum* acted as central hubs, whereas taxa such as *R. aeria* and *C. timonae* were peripheral with few connections.

Supplementary Table S6.
This table lists 142 KEGG Orthology (KO)-based pathways that were significantly associated ($q < 0.05$) in the unadjusted MaAsLin2 model. For each pathway, the KEGG annotation, effect size (coefficient), FDR-corrected q-value, direction of association (enriched or depleted), and exposure category (smoking status or periodontitis severity) are reported. The table provides the complete set of exploratory functional associations that underlie the results shown in Figure 6 and Supplementary Figures S1–S2.

Supplementary Table S7.

This table reports per-sample quality control (QC) metrics from shotgun metagenomic sequencing of subgingival plaque samples. For each participant sample, the total number of raw read pairs, percentage of host (human) reads removed, number of high-quality non-host reads retained, percentage of uniquely mapped reads, and percentage of taxonomically classified reads are listed. These QC metrics provide a comprehensive overview of sequencing quality and reproducibility, supporting the taxonomic and functional analyses described in the Methods and Results.

Supplementary Figure S1 (a–b).
Heatmaps showing functional pathways significantly associated with smoking status (a) and periodontitis severity (b) after FDR correction ($q < 0.05$). Rows represent pathways, while columns represent exposure levels. Red shading indicates enrichment and blue shading indicates depletion relative to the reference groups.

Supplementary Figure S2 (a–b).
Lollipop plots summarizing MaAsLin2 coefficients for functional pathways significantly associated with smoking status (a) and periodontitis severity (b) at $q < 0.05$. Smoking was characterized by enrichment of lipopolysaccharide biosynthesis, xenobiotic degradation, and stress-response pathways, together with depletion of amino acid biosynthesis and short-chain fatty acid metabolism. Periodontitis severity was associated with enrichment of proteolytic and sulfur metabolism pathways, and depletion of carbohydrate and energy-yielding pathways.

Supplementary Table 1: Pairwise Wilcoxon Results for Alpha Diversity by smoking status and periodontitis severity (Significant results)

Metric	Group1	Group2	p_raw	p_adj	Significant
Observed	Never | Severe	Never | Mild/Moderate	4.44E-06	3.33E-05	TRUE
Observed	Never | Severe	Former | Mild/Moderate	6.54E-08	9.82E-07	TRUE
Observed	Never | Severe	Current | Mild/Moderate	0.000663429957966	0.002821099133013	TRUE
Observed	Former | Severe	Never | Severe	0.000752293102137	0.002821099133013	TRUE
Chao1	Never | Severe	Never | Mild/Moderate	0.000344612069729	0.002584590522971	TRUE
Chao1	Never | Severe	Former | Mild/Moderate	1.92E-05	0.000288632362591	TRUE
Chao1	Never | Severe	Current | Mild/Moderate	0.001533469252091	0.007667346260454	TRUE
Chao1	Former | Severe	Never | Severe	0.002778113614972	0.010417926056144	TRUE

Supplementary Table 2 (beta-diversity diagnostics)

Supplementary table 2 a: Two-way PERMANOVA on Aitchison distances (Euclidean on CLR-transformed abundances).

Term	Df	Sum of Squares	R2	F_statistic	p_value
Smoking.status	2	22998.844379	0.008663	5.036763	0.001
Periodontitis.severity	2	123561.885095	0.046543	27.06014	0.001
Smoking.status:Periodontitis.severity	4	10548.553714	0.003973	1.15507	0.166
Residual	1094	2497708.88679	0.940821		
Total	1102	2654818.169979	1		

Supplementary table 2b: PERMDISP tests of homogeneity of multivariate dispersions for Smoking.status and Periodontitis.severity.

Factor	Df	Sum of Squares	Mean Square	F_statistic	N_permutations	p_value
Smoking.status	2	311.998734	155.999367	1.209732	999	0.283
Smoking.status	1100	141849.026856	128.953661			
Periodontitis.severity	2	7307.624458	3653.812229	28.591686	999	0.001
Periodontitis.severity	1100	140572.103645	127.792821			

Supplementary table 3c: PERMDISP Tukey post-hoc pairwise comparisons within each factor.

Factor	Df	Sum of Squares	Mean Square	F_statistic	N.Perm	Pr(>F)
Smoking.status	2	311.998734347291	155.9993671736	1.2097319784	999	0.283
Smoking.status	1100	141849.026855928	128.9536607781			
Periodontitis.severity	2	7307.62445799073	3653.812228995	28.591686029	999	0.001
Periodontitis.severity	1100	140572.103644863	127.7928214953			

Supplementary Table 3: Significant associations between metadata and microbial taxa identified by maaslin2 analysis (unadjusted model)

species	metadata	value	coef	stderr	pval	qval
Fusobacterium nucleatum J	Periodontitis.severity	Severe	4.37883334603365	0.407215751294657	1.03456764911754E-25	3.57960406594667E-23
Fretibacterium fastidiosum	Periodontitis.severity	Severe	5.7901261167692	0.537541246412646	8.63595138513003E-26	3.57960406594667E-23
Mogibacterium timidum	Periodontitis.severity	Severe	5.41353974749948	0.523854574082465	5.92473490648146E-24	1.02497913882129E-21
Treponema D sp014334325	Periodontitis.severity	Severe	4.17097955239558	0.406634504613683	1.22596758859287E-23	1.69673914261253E-21
CAJPSE01 sp905373185	Periodontitis.severity	Severe	4.71261993830986	0.487416348691726	2.80401502602578E-21	3.23396399668306E-19
Treponema D buccale	Periodontitis.severity	Severe	4.22110634515148	0.457690919762447	1.43973905053666E-19	1.24537427871422E-17
Olsenella uli	Periodontitis.severity	Severe	3.55237465470747	0.391808763020269	5.50521230258941E-19	4.23289657043541E-17
Slackia exigua	Periodontitis.severity	Severe	3.59748538629184	0.399086332591899	8.59371896610997E-19	5.40623047686191E-17
Selenomonas sp905373085	Periodontitis.severity	Severe	5.21527794127898	0.57807781062061	8.06627518020128E-19	5.40623047686191E-17
CAJPQU01 sp905373705	Periodontitis.severity	Severe	4.34419805595243	0.48797541870074	2.21058340676774E-18	1.2747697645694E-16
Tannerella forsythia	Periodontitis.severity	Severe	6.33390468773935	0.722543734572391	6.90456022658311E-18	3.67535052061193E-16
Desulfobulbus oralis	Periodontitis.severity	Severe	4.96588085188822	0.588125084214092	9.62091539147748E-17	4.75548103635887E-15
Treponema C sp905372025	Periodontitis.severity	Severe	5.92560616956713	0.705913819358226	1.4289877927052E-16	6.18037220345E-15
CAJPNN01 sp905372035	Periodontitis.severity	Severe	4.2268038097756	0.50325486187036	1.37627912909494E-16	6.18037220345E-15
Treponema B denticola	Periodontitis.severity	Severe	4.71667110403445	0.572848420963798	5.10480562063903E-16	2.07795617028365E-14
Allisonella pneumosinta	Periodontitis.severity	Severe	4.02168734339615	0.488947971135458	5.45898002294976E-16	2.09867454215624E-14
Campylobacter A rectus	Periodontitis.severity	Severe	5.47239174131779	0.669813665302903	8.41123257136577E-16	3.06345944178164E-14
Catonella morbi	Periodontitis.severity	Severe	3.84616959263797	0.482330760019052	3.8263510437104E-15	1.3239174611238E-13
Hornefia nodata	Periodontitis.severity	Severe	3.48696839255482	0.443351738704751	8.77620225121812E-15	2.89196759897283E-13
Treponema D paredis	Periodontitis.severity	Severe	2.18624054492203	0.280973453157756	1.65245593985192E-14	5.19772504717058E-13
CAJPQU01 sp905372875	Periodontitis.severity	Severe	3.81739062450215	0.494625156201018	2.64897373656038E-14	7.96995576391211E-13
Selenomonas sputigena	Periodontitis.severity	Severe	3.3761317379492	0.438028410910261	2.85729615799934E-14	8.23853725556475E-13
Parvimonas micra	Periodontitis.severity	Severe	3.20199135960195	0.447081056521787	1.45336320977655E-12	3.86818208140528E-11
Rothia dentocariosa	Periodontitis.severity	Severe	-3.23280274674694	0.457625061754158	2.85994590050922E-12	7.29276383552676E-11
Actinomyces israelii	Periodontitis.severity	Severe	3.43775371979381	0.486950425836822	2.95082929761198E-12	7.29276383552676E-11
Porphyromonas endodontalis	Periodontitis.severity	Severe	4.85921021400556	0.704939400465989	9.18460919604018E-12	2.19163778057235E-10
Actinomyces naeslundii	Smoking.status	Current	-3.51389454279606	0.515056307256059	1.47644813320229E-11	3.2958132521806E-10
Filifactor alocis	Periodontitis.severity	Severe	4.16823399816952	0.618963039427341	2.65116584543032E-11	5.73314614074308E-10
Selenomonas sp905372355	Periodontitis.severity	Severe	3.60742727098218	0.551020185968155	9.00192102374487E-11	1.88767556013074E-09
Lautropia mirabilis	Smoking.status	Current	-4.35743958905789	0.668222322996534	1.06313568441789E-10	2.16379380475641E-09
Gallibacter brachus	Periodontitis.severity	Severe	3.19103766129542	0.493232904608487	1.47612820024309E-10	2.91851632733777E-09
Eubacterium B infirmum	Periodontitis.severity	Severe	2.9875462120481	0.465256971150574	2.00700409755737E-10	3.85790787641584E-09
Saccharimonas sp905371715	Periodontitis.severity	Severe	2.79509065195989	0.437009212941267	2.3555004226343E-10	4.40542241206199E-09
Alloprevotella rava	Periodontitis.severity	Severe	3.09854934616467	0.490233840018657	3.78130042117516E-10	6.8859470827716E-09
Pseudoramibacter alactolyticus	Periodontitis.severity	Severe	3.16818281157802	0.507933311279979	6.33631333871974E-10	1.12428944369078E-08
Centipeda sp001683335	Periodontitis.severity	Severe	2.68171735725112	0.430620393796346	6.73242462983407E-10	1.16470946096129E-08

F0058 sp905372605	Periodontitis.severity	Severe	2.86523403696459	0.461717846155944	7.70705953670211E-10	1.30080126814582E-08
Dialister invisus	Periodontitis.severity	Severe	3.29703350207805	0.533852635051999	9.2446100479468E-10	1.52315956028076E-08
Treponema sp010365865	Periodontitis.severity	Severe	3.46260105078594	0.576691833828863	2.60846670564019E-09	4.19781153558839E-08
Prevotella seregens	Periodontitis.severity	Severe	3.22149525134039	0.543558823794871	4.13287817586619E-09	6.4998902220441E-08
Bacteroides D sp905373475	Periodontitis.severity	Severe	3.4296272614213	0.585774591143682	6.29821813617505E-09	9.68525988940697E-08
Lautropia dentalis	Smoking.status	Current	-3.21257306916447	0.551458263442061	7.46796846446432E-09	1.12344221248028E-07
Prevotella buccae	Periodontitis.severity	Severe	2.82352811547584	0.488846309657011	9.95615746838816E-09	1.46588531236694E-07
Lautropia dentalis	Smoking.status	Previous	-1.45418353827415	0.260777352004637	3.09046039475704E-08	4.36448692484056E-07
Neisseria sp000090875	Smoking.status	Current	-3.30031913366483	0.60128844970968	5.02449443789984E-08	6.95390030205338E-07
Nanoperiomorbus sp905372225	Periodontitis.severity	Severe	2.82575177393464	0.515382093409948	5.1908850245679E-08	7.0433185039235E-07
Tannerella sp002890585	Periodontitis.severity	Severe	2.53094676901182	0.466288671959986	7.01556287349747E-08	9.3360952085774E-07
Bulleidia moorei	Periodontitis.severity	Severe	2.48671208910962	0.45999541392695	7.90423706488194E-08	1.03202491488647E-06
F0058 sp000163695	Periodontitis.severity	Severe	3.28643369490493	0.616487753811586	1.18555792958152E-07	1.51927053198224E-06
Rothia aeria	Smoking.status	Current	-3.58308353707762	0.67377183295863	1.27081468442442E-07	1.59891593022127E-06
Alloprevotella tannerae	Periodontitis.severity	Severe	3.21977249446365	0.612403521725127	1.75291539560101E-07	2.09140940302741E-06
Nanoperiomorbus sp905373385	Periodontitis.severity	Severe	2.3430103246544	0.447830055690584	2.00803318544636E-07	2.35518468530319E-06
Prevotella oralis	Periodontitis.severity	Severe	3.11309828496895	0.600560324299144	2.58804650078226E-07	2.98488029756888E-06
Bacteroides D sp013333835	Periodontitis.severity	Severe	2.83730829025634	0.561901661864801	5.18304081047334E-07	5.78494232394766E-06
Aggregatibacter sp000466335	Smoking.status	Current	-2.74499702742744	0.543456238140951	5.14277587417645E-07	5.78494232394766E-06
Anaeroglobus geminatus	Periodontitis.severity	Severe	2.70796365104274	0.539017860727103	5.90618686202709E-07	6.21692860064445E-06
Metamycoplasma salivarium	Smoking.status	Current	2.30916711089264	0.459587378165735	5.88964867165234E-07	6.21692860064445E-06
Fusobacterium nucleatum	Periodontitis.severity	Severe	2.53074359363904	0.503819747606137	5.92944057286898E-07	6.21692860064445E-06
Prevotella sp013333935	Periodontitis.severity	Severe	2.57085762851605	0.513238889257441	6.36786340986959E-07	6.57695743228322E-06
Centipeda periodontii	Periodontitis.severity	Severe	2.63222710959309	0.526532510207277	6.69638126826884E-07	6.71579106904643E-06
Nanoperiomorbus sp905373275	Periodontitis.severity	Severe	3.02334150968296	0.605598077278019	6.93287056076862E-07	6.85363775435983E-06
Arachnia rubra	Smoking.status	Current	-3.26679554135723	0.655230617467766	7.16853025037242E-07	6.98679286374326E-06
Saccharimonas sp018127705	Periodontitis.severity	Severe	2.13150344342443	0.436189121616325	1.17795861722819E-06	1.1321491154471E-05
Olsenella F sp001189515	Periodontitis.severity	Severe	2.69733967488186	0.555428529448017	1.36896846420514E-06	1.29770709209583E-05
Fusobacterium vincentii	Periodontitis.severity	Severe	2.38057144834311	0.49244259528531	1.52667577516374E-06	1.40861284855108E-05
Prevotella oris	Periodontitis.severity	Severe	2.62135759790352	0.550106789795691	2.13970630974539E-06	1.92295683940755E-05
Treponema D buccale	Periodontitis.severity	Mild/Moderate	2.0643970999114	0.43322045937535	2.13913406776195E-06	1.92295683940755E-05
Centipeda flueggei	Periodontitis.severity	Severe	1.91880921353327	0.40505693048515	2.45121185950951E-06	2.17466488048792E-05
Tannerella serpentiformis	Smoking.status	Current	-2.72492114491333	0.576530631100766	2.58147515838026E-06	2.26124153113815E-05
Actinomyces dentalis	Periodontitis.severity	Severe	1.83955586957207	0.389975987740935	2.69996631254765E-06	2.33547086035372E-05
Cardiobacterium hominis	Smoking.status	Current	-2.80876405755543	0.595941751842022	2.75177345714194E-06	2.35089781770645E-05
Actinomyces sp000195595	Smoking.status	Current	-2.75145770734614	0.584513257298556	2.83082096053817E-06	2.38893671303953E-05
Prevotella maculosa	Periodontitis.severity	Severe	2.64115911762943	0.561574072059459	2.88751882287991E-06	2.40742533184686E-05
Actinomyces sp000195595	Smoking.status	Previous	-1.2954399256827	0.276408623380684	3.1245498135171E-06	2.57403389399266E-05

Actinomyces sp001278845	Periodontitis.severity	Severe	1.97314875247977	0.422152535434826	3.31965548315887E-06	2.70259011099522E-05
Neisseria elongata	Smoking.status	Current	-2.52822943488452	0.547229050458206	4.29002222562855E-06	3.45197137224995E-05
Cardiobacterium sp916618745	Smoking.status	Current	-2.43833002043002	0.531001604458943	4.89806579413244E-06	3.85166082902233E-05
Nanoperiomorbus periodonticus	Periodontitis.severity	Severe	1.74174938642196	0.379149116249702	4.8546599605377E-06	3.85166082902233E-05
Centipeda sp001717585	Periodontitis.severity	Severe	1.61614506578056	0.353452815767415	5.3673828948167E-06	4.17329096990242E-05
Mogibacterium timidum	Smoking.status	Current	2.56965168135652	0.562418595749793	5.45655143935313E-06	4.19548177336929E-05
Catonella morbi	Periodontitis.severity	Mild/Moderate	2.05716724949675	0.456542929745621	7.31392274214633E-06	5.50134188865789E-05
Peptoanaerobacter yurii	Periodontitis.severity	Severe	2.16480207462383	0.480817448970854	7.43768679272843E-06	5.53427877480438E-05
Metamycoplasma salivarium	Periodontitis.severity	Severe	1.91203956017325	0.428074306330004	8.76784065959485E-06	6.45462312387195E-05
Actinomyces massiliensis	Smoking.status	Current	-2.490796885267	0.561621705312374	1.01313147716187E-05	7.37986297048436E-05
Corynebacterium durum	Smoking.status	Current	-3.09352155691347	0.706272394300994	1.29978494264994E-05	9.27269258055419E-05
Actinomyces sp002999235	Periodontitis.severity	Severe	2.55957363793872	0.588386017802018	1.48688195537116E-05	0.000104992072767
Treponema D paredis	Periodontitis.severity	Mild/Moderate	1.14629654901418	0.265951198054047	1.77749085901754E-05	0.0001242448156
Fusobacterium animalis	Periodontitis.severity	Severe	1.73680995231273	0.414557771291902	3.01916525899616E-05	0.000206857659329
Prevotella denticola	Periodontitis.severity	Severe	2.64431027272905	0.634424531863795	3.31358396166E-05	0.000224803931517
Centipeda sp905372865	Periodontitis.severity	Severe	1.72600543433493	0.41524904053557	3.4819084387596E-05	0.000233930159187
Johnsonella sp900766185	Smoking.status	Current	-1.75634048695474	0.42661387656431	4.126883189506E-05	0.000274596458379
Oribacterium sp000160135	Periodontitis.severity	Severe	2.27920870536775	0.555553287664333	4.38712937753329E-05	0.000289132717072
Prevotella conceptionensis	Periodontitis.severity	Severe	2.38622277938104	0.585872705301625	4.9755995803014E-05	0.000318806936071
Actinomyces oris	Periodontitis.severity	Severe	-1.91994984646531	0.47457383474163	5.5831123569127E-05	0.000354450802843
Aggregatibacter sp000466335	Smoking.status	Previous	-1.03858671677532	0.256993299598436	5.68569225191979E-05	0.000357681730757
Lautropia mirabilis	Smoking.status	Previous	-1.27401629657978	0.315993538393558	5.9174970344671E-05	0.000366631133303
Campylobacter A sp905372745	Smoking.status	Current	-2.12826193953365	0.52795811909062	5.9339142962401E-05	0.000366631133303
Campylobacter B gracilis A	Periodontitis.severity	Severe	1.16981497017774	0.291086847166375	6.24847401872435E-05	0.000381657049442
Prevotella pleuritidis	Periodontitis.severity	Severe	3.13861549773095	0.800575104775061	9.38524039955803E-05	0.000559878134181
Pseudoramibacter alactolyticus	Smoking.status	Current	2.13289711073341	0.545325274986829	9.74545042683969E-05	0.000576397580801
Arachnia rubra	Smoking.status	Previous	-1.1954768039959	0.309849931904339	0.000120847350107	0.000702742573733
Capnocytophaga periodontitidis	Periodontitis.severity	Severe	2.11117500317815	0.551678701418617	0.000137125696312	0.000790758182066
Eikenella exigua	Periodontitis.severity	Severe	1.78764785906795	0.471670423650494	0.000158805065375	0.000908207481318
Neisseria sp000090875	Smoking.status	Previous	-1.06315438181238	0.284341391001276	0.000194272347731	0.001101938234673
Capnocytophaga granulosa	Smoking.status	Current	-2.00252452187994	0.537159318567424	0.000202850744347	0.001141241586083
Cardiobacterium sp916618745	Smoking.status	Previous	-0.926773237399031	0.251103667314192	0.000234515326281	0.001308746820858
Leptotrichia buccalis	Smoking.status	Previous	-0.65757387380742	0.1793302409147	0.000257322669321	0.001424538297364
CAJPQU01 sp905372875	Periodontitis.severity	Mild/Moderate	1.71070776314098	0.468180005623067	0.000270422897099	0.001485179720575
Actinomyces johnsonii	Smoking.status	Current	-2.09123927344711	0.574413426551335	0.000284608032938	0.00155077762829
Corynebacterium durum	Smoking.status	Previous	-1.21229294137872	0.333986916127043	0.00029673838764	0.001604241908181
Nanoperiomorbus sp905373275	Smoking.status	Previous	-1.11364916601075	0.307461428916698	0.000305587705009	0.001639276681135
Prevotella saccharolytica	Smoking.status	Current	-2.06332595392507	0.572135124087587	0.000324463336753	0.001727143300254

Fretibacterium fastidiosum	Periodontitis.severity	Mild/Moderate	1.82124078375095	0.508801585631089	0.000359314413602	0.001898057818418
Actinomyces israelii	Smoking.status	Current	1.87011753301965	0.522797715718326	0.000362503960929	0.001900399552748
Fusobacterium nucleatum J	Smoking.status	Current	1.56237511057723	0.437193301998896	0.000367309772386	0.00191111550745
Actinomyces sp915069725	Periodontitis.severity	Severe	-1.950804466665	0.546987330661995	0.000377419532904	0.001949062065444
Selenomonas sp905373085	Periodontitis.severity	Mild/Moderate	1.94824770260288	0.5471708610731	0.000385857613745	0.00197787754601
Lancefieldella rimae	Periodontitis.severity	Severe	1.73418055769253	0.487323659998495	0.000388777792697	0.00197819288637
Nanoperiomorbus sp905372225	Smoking.status	Previous	-0.929994432634298	0.261658880408161	0.000395199639793	0.001996190881292
Centipeda infelix	Smoking.status	Current	-1.8372373397596	0.518250059084003	0.00040900684669	0.002050961868911
Actinomyces massiliensis	Periodontitis.severity	Severe	-1.83777214422356	0.523112324975058	0.000460827170736	0.002277802872494
Prevotella saccharolytica	Periodontitis.severity	Severe	1.85441577566013	0.532904857718925	0.000521412687882	0.002558989929182
Slackia exigua	Periodontitis.severity	Mild/Moderate	1.31234259992265	0.377749168424887	0.00053251958612	0.002595095447855
Treponema D sp014334325	Periodontitis.severity	Mild/Moderate	1.33430630308952	0.384893777176179	0.000547280092102	0.002648376389755
Treponema sp010365865	Periodontitis.severity	Mild/Moderate	1.88626398473861	0.545858985577043	0.000570059866183	0.002721260185369
Slackia exigua	Smoking.status	Current	1.4805701887522	0.428465428888166	0.000570206252715	0.002721260185369
Campylobacter B sp905373215	Periodontitis.severity	Severe	1.16211188888095	0.33804866840279	0.00060863132369	0.002884745725984
CAJPQU01 sp905373705	Periodontitis.severity	Mild/Moderate	1.58475439097761	0.461885796561435	0.000623592993053	0.002935553409472
Capnocytophaga granulosa	Periodontitis.severity	Severe	1.70398293692062	0.500327279661554	0.000683709900368	0.003133293053343
Fusobacterium nucleatum J	Periodontitis.severity	Mild/Moderate	1.31132043356469	0.385443947483846	0.000692765777752	0.003153907356609
Corynebacterium matruchotii	Periodontitis.severity	Severe	-1.60847015924016	0.474735816132906	0.000728634808375	0.003295524754218
Centipeda infelix	Periodontitis.severity	Severe	1.63374771376167	0.482714593758634	0.00073825480275	0.003317352750019
Treponema B denticola	Periodontitis.severity	Mild/Moderate	1.82985612178009	0.542221060909809	0.000764653028208	0.003370317805861
Mogibacterium timidum	Periodontitis.severity	Mild/Moderate	1.67503274926137	0.495846671696422	0.00075539943191	0.003370317805861
Actinomyces naeslundii	Smoking.status	Previous	-0.822308213518966	0.243563346210758	0.000760769930134	0.003370317805861
Fretibacterium fastidiosum	Smoking.status	Current	1.92728427244626	0.577112824669929	0.000867385304942	0.003798928044431
Treponema C lecithinolyticum	Periodontitis.severity	Severe	2.2687146174567	0.682200657737346	0.000911501592151	0.003967038375902
Nanoperiomorbus sp905373385	Smoking.status	Previous	-0.751706220403509	0.227362790604205	0.000976293943475	0.004222471305529
Gemella morbillorum	Smoking.status	Previous	-0.824912920736076	0.25030889141546	0.001013565925165	0.004355750886491
Tannerella serpentiformis	Periodontitis.severity	Severe	1.768803459119	0.536998972799162	0.001019698906953	0.004355750886491
Granulicatella adiacens	Periodontitis.severity	Severe	-1.52718752291107	0.464052077973989	0.001030108076784	0.004373219565242
Treponema C sp905372025	Periodontitis.severity	Mild/Moderate	2.19759898377742	0.668172113312855	0.001037425700573	0.004377430395099
Prevotella nigrescens	Periodontitis.severity	Severe	1.97920691917876	0.602442211997992	0.001050883806243	0.004407342993456
Olsenella uli	Periodontitis.severity	Mild/Moderate	1.20981046703376	0.37086069435467	0.001139629977692	0.004750746653994
Pauljensenia hongkongensis	Smoking.status	Previous	-0.727829378354498	0.22688222063202	0.0013754115615	0.005647525481725
Parvimonas micra	Smoking.status	Current	1.53859044007814	0.47999332722393	0.001387397878458	0.005647525481725
Nanoperiomorbus periodonticus	Smoking.status	Previous	-0.617404083027699	0.192493558728918	0.001378067005797	0.005647525481725
Actinomyces sp000220835	Smoking.status	Previous	-1.00711620855374	0.315426115257835	0.001448622071186	0.005841300487902
Centipeda timonae	Periodontitis.severity	Severe	1.24525359273067	0.390090214107633	0.001451883936299	0.005841300487902
Actinomyces oris E	Periodontitis.severity	Severe	-1.7249846000527	0.541702912502872	0.001491445509628	0.005965782038514

Streptococcus gordonii	Periodontitis.severity	Severe	-1.93826548724578	0.612739232924122	0.001602797665647	0.006374344739239
Prevotella buccae	Smoking.status	Current	1.65500305142938	0.524833166716769	0.001657604345889	0.006554641184886
Prevotella oulorum	Periodontitis.severity	Severe	1.64514283892797	0.52333209225668	0.001713585469607	0.006699441496993
Capnocytophaga periodontitidis	Smoking.status	Previous	-0.88062415385938	0.280086625445488	0.00171056462345	0.006699441496993
Capnocytophaga periodontitidis	Periodontitis.severity	Mild/Moderate	1.63943361885557	0.522183181130655	0.001737038786613	0.006752982249081
Capnocytophaga periodontitidis	Smoking.status	Current	-1.85127993474648	0.59229102103456	0.001820805532067	0.007039091777601
Prevotella oralis	Smoking.status	Current	2.01104563657625	0.64477110817819	0.00186183390347	0.007157717006673
Campylobacter B sp905373295	Periodontitis.severity	Severe	1.03370678011834	0.332249200128223	0.001911070599052	0.007306413561018
Rothia dentocariosa	Periodontitis.severity	Mild/Moderate	-1.34617627113115	0.433158122467685	0.001933139745681	0.00735017969237
Actinomyces naeslundii	Periodontitis.severity	Severe	-1.48338790416881	0.479739831693161	0.002037762643289	0.007663759506281
Burkholderia sp018375725	Periodontitis.severity	Severe	-1.36774492994694	0.442157003448775	0.002029074031349	0.007663759506281
Prevotella conceptionensis	Smoking.status	Previous	-0.918530652841948	0.297446880850372	0.0020652741423	0.007725241656604
Actinomyces massiliensis	Smoking.status	Previous	-0.818215763816326	0.265583509848117	0.002115613705683	0.007828902055255
Actinomyces israelii	Smoking.status	Previous	0.761041550661238	0.247224156345283	0.002133123941849	0.007851711530637
Selenomonas sputigena	Periodontitis.severity	Mild/Moderate	1.26890510343194	0.414609207218902	0.002263399732109	0.008243540076946
Centipeda infelix	Smoking.status	Previous	-0.749349519687006	0.245073629399648	0.00228472884198	0.008277656328012
Arachnia propionica	Periodontitis.severity	Mild/Moderate	1.36352155385219	0.44746005841508	0.002364612005282	0.008522455769039
Allisonella pneumosinta	Periodontitis.severity	Mild/Moderate	1.40567800962916	0.462806351447587	0.002443449089186	0.008760967718738
Eikenella corrodens	Periodontitis.severity	Severe	1.31773623716428	0.434314936360401	0.002469581007233	0.008809020912396
Campylobacter B sp900539505	Periodontitis.severity	Severe	0.983112072490015	0.324822155009643	0.0025306580817	0.008980591756595
SDRW01 sp007845485	Smoking.status	Current	-1.68932599441766	0.558805294806916	0.002559976735693	0.009038285209693
Leptotrichia hofstadii	Smoking.status	Previous	-0.648424772961978	0.21595385084462	0.00273729313444	0.009615263193057
Actinomyces oris E	Smoking.status	Previous	-0.824329625012518	0.275021929189529	0.002784802681671	0.00973274472584
Peptidiphaga sp000466165	Smoking.status	Current	-1.86426415809353	0.622426433485899	0.002804504729775	0.00975234810555
Gemella morbillorum	Smoking.status	Current	-1.58001848111932	0.529320915037216	0.00289860950568	0.010029188889652
Prevotella saccharolytica	Smoking.status	Previous	-0.803260203394595	0.27055516716195	0.003053181107082	0.010511449383585
Saccharimonas sp018127705	Periodontitis.severity	Mild/Moderate	1.22263308372298	0.412868255588799	0.003129108218589	0.010719519243881
Actinomyces oris D	Smoking.status	Previous	-0.689106613365972	0.235010599908634	0.003435052733638	0.011709637890038
Capnocytophaga sputigena	Smoking.status	Current	-1.67388154113411	0.575147847931586	0.003683041433487	0.012493454274376
Campylobacter A rectus	Periodontitis.severity	Mild/Moderate	1.82647875954154	0.63400205520577	0.004042814995208	0.013646965739922
Lachnoanaerobaculum saburreum	Smoking.status	Previous	-0.748805962032461	0.260852697170862	0.004175390718321	0.013958311000378
CAJPSE01 sp905373185	Periodontitis.severity	Mild/Moderate	1.32481265785211	0.461356617249217	0.004162951156682	0.013958311000378
Capnocytophaga leadbetteri	Smoking.status	Current	-1.77666089083017	0.619854466878811	0.004232810385872	0.014082234552998
Actinomyces dentalis	Periodontitis.severity	Mild/Moderate	1.05027691273177	0.369125908467161	0.004519233159731	0.014963202614994
Actinomyces sp000195595	Periodontitis.severity	Severe	-1.54611305657516	0.544434244816312	0.004596659392898	0.015147087142311
Arachnia propionica	Periodontitis.severity	Severe	1.34090273100915	0.472734842643974	0.004645132909446	0.01523427475515
Arachnia sp905372155	Smoking.status	Current	-1.86923902399638	0.663433613968541	0.004926285006138	0.016080137850223
CAJPSE01 sp905373185	Smoking.status	Current	1.46907207730286	0.523297937899724	0.005083673087914	0.01651597078327

Actinomyces johnsonii	Periodontitis.severity	Severe	-1.49432255278877	0.5350269411205	0.005313045409656	0.017180501978888
Centipeda sp905372865	Smoking.status	Current	-1.23952389677577	0.445817968991715	0.005522975598972	0.017776274951109
Porphyromonas catoniae	Smoking.status	Current	-1.60164039718719	0.577213639769148	0.005617550635572	0.017996967776926
Peptoanaerobacter yurii	Periodontitis.severity	Mild/Moderate	1.2546343168479	0.455110527923408	0.005934209840482	0.01892383967564
Streptococcus mutans	Smoking.status	Current	1.52108801189856	0.556898635859002	0.006408426445913	0.020342344498036
Actinomyces oris A	Periodontitis.severity	Severe	-1.48937664200079	0.545839177266443	0.006461844440114	0.0204182481852
Centipeda felix	Periodontitis.severity	Severe	1.24325898729488	0.455969572152502	0.00650033234783	0.020446499930447
Eubacterium B infirmum	Periodontitis.severity	Mild/Moderate	1.19617571950819	0.440381991572063	0.006706758923571	0.021000349208649
Capnocytophaga granulosa	Periodontitis.severity	Mild/Moderate	1.27899288092232	0.473577264861399	0.00702560905637	0.021899646247784
Johnsonella sp900766185	Smoking.status	Previous	-0.543755491671833	0.201740085214196	0.007139276292267	0.022154166790354
SDRW01 sp007845485	Smoking.status	Previous	-0.711033363715276	0.264251666402364	0.0072374439134	0.022358532089611
Centipeda artemidis	Smoking.status	Current	-1.43016950931453	0.532314485071433	0.007325030545119	0.022528538387655
Corynebacterium matruchotii	Smoking.status	Previous	-0.64439050530592	0.241022813418129	0.007616183512384	0.023217616698546
Actinomyces johnsonii	Smoking.status	Previous	-0.726363030182047	0.27163254639978	0.007604949027828	0.023217616698546
Fusobacterium vincentii	Smoking.status	Current	1.41214840513473	0.528694196118919	0.007674008458555	0.023291288830351
Corynebacterium durum	Periodontitis.severity	Severe	-1.7522508603561	0.657844578928801	0.007843442909233	0.023460323266204
Pauljensenia meyeri	Periodontitis.severity	Severe	1.21665298076252	0.456927734796966	0.007865310690404	0.023460323266204
Treponema D sp014334325	Smoking.status	Current	1.16323468129402	0.43656926632512	0.007823404708215	0.023460323266204
Actinomyces oris	Smoking.status	Current	1.35378716330063	0.50951001083168	0.00799734178894	0.0237517618796
Porphyromonas catoniae	Smoking.status	Previous	-0.723032656731106	0.272956730361467	0.008191415236774	0.024224185230117
Streptococcus intermedius	Periodontitis.severity	Severe	-1.62444715302746	0.616916917921527	0.00857791624253	0.025152195084029
Tannerella serpentiformis	Smoking.status	Previous	-0.716562186143472	0.272633744555024	0.008701016190439	0.025405498750143
Bacteroides D sp905373475	Smoking.status	Previous	-0.781119189425539	0.297397068408209	0.008746277028269	0.025430351695638
Kingella B oralis	Periodontitis.severity	Severe	-1.25865012608947	0.480222367638937	0.008888927201693	0.025736977504482
Porphyromonas pasteri	Smoking.status	Current	-1.46546480234116	0.564218835411451	0.009520591822803	0.027451039755747
Veillonella sp900757715	Smoking.status	Previous	-0.603474764353331	0.233172241564387	0.009777819399423	0.028075730391705
Anaeroglobus geminatus	Smoking.status	Current	1.48751523947603	0.578698141264053	0.010287388264398	0.029416829251916
Actinomyces oris D	Smoking.status	Current	-1.2629901935076	0.496970064002289	0.011178078531378	0.031832223636681
Streptococcus gordonii	Smoking.status	Previous	-0.786106075163884	0.311086985207986	0.011644687436977	0.033025097157329
Prevotella seregens	Smoking.status	Previous	0.692126660813805	0.275964173161546	0.012283835980904	0.034695569382797
Leptotrichia buccalis	Smoking.status	Current	-0.948189830842838	0.379224432172731	0.012552225652912	0.035063116617505
Centipeda periodontii	Smoking.status	Current	-1.41345099875904	0.565293670530732	0.012550673933185	0.035063116617505
Granulicatella adiacens	Smoking.status	Previous	-0.585135182831776	0.235598692167156	0.013154038190516	0.036556604127859
Nanoperiomorbus sp905372225	Periodontitis.severity	Mild/Moderate	1.21076616490864	0.487827172487433	0.013214844608356	0.036578689875929
CAJPQU01 sp905373705	Smoking.status	Current	1.29772880400664	0.523898164346055	0.013396401148183	0.036933504360726
Actinomyces oris E	Smoking.status	Current	-1.43783439404642	0.581580855520942	0.013575729525477	0.037279384252499
Actinomyces gerencseriae	Smoking.status	Current	-1.23512078209359	0.501013259846035	0.013843919340786	0.037721987208368
Leptotrichia hofstadii	Smoking.status	Current	-1.1179990423517	0.456671312347256	0.014514784409893	0.039389140437827

Capnocytophaga gingivalis	Smoking.status	Current	-1.31547688458808	0.538306594235337	0.014692675617729	0.039716138779173
Olsenella F sp001189515	Periodontitis.severity	Mild/Moderate	1.28341680555326	0.525732524478603	0.014795625819758	0.039838805709231
Pauljensenia hongkongensis	Smoking.status	Current	-1.17041147694411	0.479781217325144	0.014866188296252	0.039873652329482
Metamycoplasma salivarium	Periodontitis.severity	Mild/Moderate	0.986877765562809	0.405187299174129	0.015024885068215	0.040143708367587
Johnsonella ignava	Smoking.status	Current	-1.31338216158599	0.540716261567314	0.015301413168958	0.040725299665072
Centipeda flueggei	Periodontitis.severity	Mild/Moderate	0.928186637346812	0.383400548101378	0.015641972453837	0.041472202827798
Actinomyces sp001278845	Periodontitis.severity	Mild/Moderate	0.965703025372886	0.39958213595862	0.015820445811591	0.041785299624507
Selenomonas sp905372355	Periodontitis.severity	Mild/Moderate	1.25753134335351	0.521559873230854	0.016067883415855	0.04227747271396
Actinomyces gerencseriae	Smoking.status	Previous	-0.568517592407357	0.23692257398839	0.016579011242134	0.043457105225593
Campylobacter A sp905372745	Periodontitis.severity	Severe	1.17898579085975	0.491756989722006	0.016673638730672	0.043540218874056
Neisseria elongata	Smoking.status	Previous	-0.619358870252744	0.25877741286852	0.016859825918684	0.043549807821
Tannerella sp002890585	Periodontitis.severity	Mild/Moderate	1.05641265905267	0.441358532463071	0.016853257955894	0.043549807821
Prevotella maculosa	Smoking.status	Previous	-0.681107074140637	0.285110493438214	0.017065749268841	0.043873678206202
Actinomyces oris D	Periodontitis.severity	Mild/Moderate	1.0461969309994	0.438145100518547	0.017118342652709	0.043873678206202
Gallibacter brachus	Smoking.status	Current	1.26215282647484	0.529542684767754	0.017319016727842	0.044224205076261
Bulleidia moorei	Periodontitis.severity	Mild/Moderate	1.03655973887519	0.435401743681999	0.017449554281316	0.044393718980407
Johnsonella sp900766185	Periodontitis.severity	Severe	0.942950574771498	0.397361737848171	0.017814185757918	0.045155371957799
Metamycoplasma salivarium	Smoking.status	Previous	0.515070335490814	0.217332819975788	0.017961867217746	0.045363547863797
Gallibacter brachus	Periodontitis.severity	Mild/Moderate	1.10470800236587	0.466862190808661	0.018142486973448	0.045653094493185
Campylobacter A sp905372745	Periodontitis.severity	Mild/Moderate	1.09993034575619	0.465465185761123	0.018297239705926	0.045875687958337
Centipeda sp001683335	Periodontitis.severity	Mild/Moderate	0.96072997132786	0.407597259988625	0.018595055684737	0.046454074129379
Capnocytophaga leadbetteri	Smoking.status	Previous	-0.689885847332579	0.29312101607103	0.018768619551751	0.046719009819466
Olsenella F sp001189515	Smoking.status	Previous	-0.662863647929962	0.281990408709288	0.018916123166647	0.046917409431253
Centipeda sp001717585	Periodontitis.severity	Mild/Moderate	0.780653767117382	0.334555449109073	0.019806469152948	0.04877607350121
Prevotella buccae	Smoking.status	Previous	0.577684632428218	0.248186694322674	0.02011307914896	0.049355499188229
Lancefieldella rimae	Smoking.status	Current	1.21607255770039	0.523198425845677	0.020290727272237	0.049615488595009
Lachnoanaerobaculum saburreum	Periodontitis.severity	Severe	1.1899380768512	0.513794177097445	0.020742566851924	0.050189435339856
Eikenella corrodens	Smoking.status	Previous	-0.511127907235917	0.220501180475066	0.020630684402858	0.050189435339856
Prevotella sp013333935	Periodontitis.severity	Mild/Moderate	1.12509659187847	0.485798554816875	0.020743032524854	0.050189435339856
Cardiobacterium valvarum	Smoking.status	Current	-1.1783212739933	0.510797642923604	0.021249773399876	0.051058483308035
Nanoperiomorbus sp905373385	Periodontitis.severity	Mild/Moderate	0.977938521858956	0.42388680673206	0.02123585451451	0.051058483308035
Prevotella pleuritidis	Smoking.status	Current	1.9789460439198	0.859510155100705	0.021498160436745	0.051476564090752
Desulfobulbus oralis	Periodontitis.severity	Mild/Moderate	1.27589388916949	0.556680956846679	0.022095983606503	0.05272558846793
Aggregatibacter sp000466335	Periodontitis.severity	Severe	1.15416486596213	0.506192430896149	0.022793170559177	0.054202316243816
Streptococcus mutans	Periodontitis.severity	Severe	-1.18076642441452	0.518713107816244	0.023017454797761	0.054362043413142
Tannerella serpentiformis	Periodontitis.severity	Mild/Moderate	1.1576345498871	0.508288304694552	0.022946880775826	0.054362043413142
CAJPNN01 sp905372035	Smoking.status	Current	1.22229152671979	0.540302417351477	0.023877468013556	0.056201387297214
Johnsonella ignava	Smoking.status	Previous	-0.576345438838523	0.255697600752674	0.024391776482014	0.05721731974764

Leptotrichia A sp001274535	Smoking.status	Current	-1.14507423520542	0.510043056553129	0.024962892166735	0.058359193849258
Fusobacterium polymorphum	Smoking.status	Previous	-0.494372120625893	0.220467430069774	0.025135503844448	0.058427195666887
CAJPNN01 sp905372035	Periodontitis.severity	Mild/Moderate	1.06796849663077	0.476348323789149	0.025160844376781	0.058427195666887
Bacteroides D sp013333835	Periodontitis.severity	Mild/Moderate	1.19131865476423	0.531859570653459	0.025295936186868	0.058447155663681
Prevotella oralis	Smoking.status	Previous	0.682759183199304	0.304903767676464	0.025338362281943	0.058447155663681
Capnocytophaga granulosa	Smoking.status	Previous	-0.565369092144042	0.254015569240528	0.026234782201123	0.060114136699262
Prevotella salivae	Periodontitis.severity	Severe	1.20564869822246	0.541464103300724	0.026173025139104	0.060114136699262
Actinomyces sp000220835	Periodontitis.severity	Severe	-1.37612909292625	0.621285894612733	0.026966659608674	0.061587222604628
Tannerella forsythia	Smoking.status	Current	1.7099037656301	0.775734435988803	0.027714259816893	0.063086407214769
Hornefia nodata	Smoking.status	Current	1.04829182033806	0.475989472349826	0.027848206938567	0.063183472791766
Fusobacterium animalis	Periodontitis.severity	Mild/Moderate	0.863637716090736	0.392393426135509	0.027947036520253	0.063200487817043
Nanoperiomorbus sp905373275	Periodontitis.severity	Mild/Moderate	1.25140865494788	0.573219755749977	0.029237837802152	0.065904181625698
Arachnia rubra	Periodontitis.severity	Severe	-1.31704616779388	0.610302644032896	0.031142252576339	0.069820191457968
Centipeda sp905372865	Smoking.status	Previous	-0.454863434284642	0.210821447672367	0.031176935203052	0.069820191457968
Actinomyces israelii	Periodontitis.severity	Mild/Moderate	0.993125919868012	0.460915604975389	0.031403590135189	0.070100917334034
Saccharimonas sp905371715	Periodontitis.severity	Mild/Moderate	0.889849765315238	0.413644500703528	0.03167448045923	0.070478265201888
Lachnoanaerobaculum saburreum	Periodontitis.severity	Mild/Moderate	1.04444808946507	0.486324154173876	0.031960709534979	0.070660738013435
Prevotella maculosa	Smoking.status	Current	-1.27577665894417	0.602914848210249	0.034567956269665	0.07593976424955
Desulfobulbus oralis	Smoking.status	Current	1.3316569374164	0.631420436803991	0.035171540581045	0.077021221778744
Cardiobacterium hominis	Periodontitis.severity	Mild/Moderate	1.10448082422442	0.525401785091866	0.035765972797461	0.078075877526319
Pauljensenia sp000185285	Smoking.status	Current	1.05172520524497	0.502348028342477	0.036522681704953	0.079477030628388
Prevotella saccharolytica	Periodontitis.severity	Mild/Moderate	1.05117286261647	0.504413081614494	0.03739505194303	0.081120300766698
Saccharimonas sp018127705	Smoking.status	Previous	-0.459235116679123	0.221452702116996	0.038336033527784	0.082342141919167
Centipeda noxia	Smoking.status	Previous	-0.535015806722497	0.258126901780601	0.038434265664582	0.082342141919167
Treponema C sp905372025	Smoking.status	Previous	-0.741729304832361	0.358391612746624	0.03872254371564	0.082703704479083
Allisonella pneumosinta	Smoking.status	Current	1.07991486726804	0.524942312095618	0.039901897349219	0.084960347586644
Dialister invisus	Periodontitis.severity	Mild/Moderate	1.03612896869899	0.505310186000645	0.040554299097212	0.086084585813713
Porphyromonas endodontalis	Periodontitis.severity	Mild/Moderate	1.36201823295413	0.667249791759397	0.041465259217592	0.08764881129398
Leptotrichia wadei	Periodontitis.severity	Severe	0.950249844712991	0.465706895980194	0.041544523272291	0.08764881129398
Peptoanaerobacter yurii	Smoking.status	Current	-1.05206507275657	0.516213254290715	0.041784629805675	0.087887428041115
Alloprevotella rava	Periodontitis.severity	Mild/Moderate	0.93739456128497	0.464023471307787	0.043610618577605	0.091173861195476
F0058 sp905372605	Periodontitis.severity	Mild/Moderate	0.882303276323422	0.437032086014058	0.043745029634265	0.091179399117203
Bacteroides D sp905373475	Periodontitis.severity	Mild/Moderate	1.11371735614959	0.554456132967171	0.044816780902914	0.092588289044466
Neisseria sp000090875	Periodontitis.severity	Severe	-1.12615835740399	0.56005919275027	0.044592100560717	0.092588289044466
Prevotella seregens	Smoking.status	Current	1.17217355474979	0.583573391377883	0.044822365361121	0.092588289044466
Centipeda sp001717585	Smoking.status	Current	-0.758571297359999	0.379472560024696	0.045852654378423	0.094434633422228
Oribacterium sp000160135	Periodontitis.severity	Mild/Moderate	1.05042768453495	0.525850612492696	0.046008573153774	0.094474577514574
Tannerella sp002890585	Smoking.status	Previous	-0.472219803816062	0.236734208293515	0.046319451010451	0.094831538755125

Cardiobacterium hominis	Smoking.status	Previous	-0.560796928061765	0.281813007976977	0.046842670621759	0.095059026598995
Olsenella uli	Smoking.status	Current	0.83765557406224	0.420652114542067	0.046692258323474	0.095059026598995
Porphyromonas pasteri	Smoking.status	Previous	-0.529460377208968	0.266811658476848	0.047459936944256	0.096030047852121
Treponema sp010365865	Smoking.status	Previous	-0.578538545741202	0.292785763241803	0.048406934961361	0.097376741259482
Capnocytophaga leadbetteri	Periodontitis.severity	Severe	1.13869232894226	0.577352171841007	0.048829596884848	0.097379407742806
Prevotella maculosa	Periodontitis.severity	Mild/Moderate	1.04894786413967	0.531549495448054	0.048702777173087	0.097379407742806
Campylobacter B gracilis A	Periodontitis.severity	Mild/Moderate	0.543404509787597	0.275523879112548	0.048830425558893	0.097379407742806
Actinomyces gerencseriae	Periodontitis.severity	Severe	-0.91099861029098	0.466659690539595	0.051171786613772	0.101463829045072
Pseudoramibacter alactolyticus	Smoking.status	Previous	0.501832863427303	0.257877142514172	0.051907525466993	0.102336204054585
Cardiobacterium valvarum	Smoking.status	Previous	-0.470116558575708	0.241549479919658	0.05187886299763	0.102336204054585
Eikenella exigua	Smoking.status	Previous	-0.461402524214694	0.239466517273549	0.054262962362712	0.106676051008514
Actinomyces sp002999235	Smoking.status	Previous	-0.57479161215011	0.298722886639819	0.054592106581081	0.10701908712212
Hornefia nodata	Periodontitis.severity	Mild/Moderate	0.799111910008052	0.419647923114186	0.057139383188921	0.111393971145399
Centipeda sp905372865	Periodontitis.severity	Mild/Moderate	0.748439468083916	0.393047736645863	0.057145751093377	0.111393971145399
Peptidiphaga sp000466165	Smoking.status	Previous	-0.558262938496845	0.294337265215715	0.058132497602745	0.112999124553651
Corynebacterium matruchotii	Smoking.status	Current	-0.963863893653543	0.509683916627537	0.05887363106426	0.114119195228202
Prevotella denticola	Periodontitis.severity	Mild/Moderate	1.13200098693461	0.60050500297379	0.059682450325935	0.114878706087576
Aggregatibacter aphrophilus	Smoking.status	Previous	-0.495048655055692	0.262674101357245	0.059740854013614	0.114878706087576
Lautropia dentalis	Periodontitis.severity	Severe	-0.967956372612825	0.513645771855336	0.05976348871608	0.114878706087576
Oribacterium sp000160135	Smoking.status	Current	1.118958188943	0.596450838402279	0.060916381128702	0.116127095705405
Prevotella buccae	Periodontitis.severity	Mild/Moderate	0.862626933245626	0.462710125303497	0.062547110740578	0.118908243495823
Eikenella corrodens	Periodontitis.severity	Mild/Moderate	0.764964763202046	0.411094273710489	0.063039143146958	0.119515307007383
Prevotella oulorum	Periodontitis.severity	Mild/Moderate	0.920147229707986	0.49535212437899	0.063498577035193	0.120057418875283
Campylobacter B sp900539505	Periodontitis.severity	Mild/Moderate	0.570625712325444	0.307455527589679	0.063726532957721	0.120160111190035
Capnocytophaga ochracea	Smoking.status	Previous	-0.457322092437701	0.247417440050578	0.064815506272022	0.121881332446302
Fusobacterium polymorphum	Periodontitis.severity	Mild/Moderate	0.755095548325155	0.411031350698824	0.066468671141622	0.124651274878055
Centipeda periodontii	Periodontitis.severity	Mild/Moderate	0.911365865196846	0.498381431876439	0.067722765761757	0.126318474143223
Olsenella uli	Smoking.status	Previous	0.360201365700353	0.198920846449438	0.070448292639216	0.130697636746213
Eikenella corrodens	Smoking.status	Current	-0.840444203481542	0.466287417741484	0.071753773240853	0.132763665996445
SDRW01 sp007845485	Periodontitis.severity	Mild/Moderate	0.885750689202411	0.492661067130886	0.072468734272123	0.13337330881997
Pauljensenia georgiae	Periodontitis.severity	Severe	-0.787174045369422	0.437652703372799	0.072351980926609	0.13337330881997
Tannerella forsythia	Periodontitis.severity	Mild/Moderate	1.22645585299691	0.683912909551926	0.073201048161285	0.134008268062458
Parvimonas micra	Periodontitis.severity	Mild/Moderate	0.759161320723942	0.423177852828962	0.073095420720874	0.134008268062458
Campylobacter B sp905373295	Periodontitis.severity	Mild/Moderate	0.56270490793976	0.314485485491712	0.07384383906103	0.134828328839664
Capnocytophaga sp000192225	Smoking.status	Previous	-0.439160665831878	0.246832943473394	0.075486293284178	0.137464513033293
Capnocytophaga sputigena	Periodontitis.severity	Mild/Moderate	0.898242277223674	0.507069197721032	0.076764771136567	0.139425778547255
Peptoanaerobacter yurii	Smoking.status	Previous	-0.430533126667119	0.244110451230499	0.078063413138246	0.141413303381324
Fusobacterium polymorphum	Smoking.status	Current	-0.817449751245692	0.466216046743389	0.079817093111471	0.144212606875034

Nanoperiomorbus periodonticus	Periodontitis.severity	Mild/Moderate	0.626774763627908	0.358877896023601	0.081006133704523	0.145979803446693
Fusobacterium vincentii	Periodontitis.severity	Mild/Moderate	0.804505520650893	0.466114135399973	0.084631363225655	0.152116632083515
Leptotrichia hongkongensis	Smoking.status	Current	-0.919756596025932	0.538137752993263	0.087706018975453	0.157234624691746
Alloprevotella tannerae	Periodontitis.severity	Mild/Moderate	0.987307196772302	0.579661346881301	0.088804344043063	0.158383005355153
Prevotella nigrescens	Smoking.status	Previous	-0.518243857888543	0.305859199839573	0.09047508407389	0.160947964470776
Neisseria elongata	Periodontitis.severity	Mild/Moderate	0.813640986917024	0.482455070610803	0.09199112694007	0.163225281647508
Campylobacter B sp905373215	Periodontitis.severity	Mild/Moderate	0.538908638595458	0.319974885000325	0.092422787589785	0.16357178775481
Pauljensenia sp000185285	Smoking.status	Previous	-0.399488228041707	0.237553768436123	0.092915987253397	0.164025161171813
Campylobacter B gracilis	Periodontitis.severity	Severe	0.64626383107233	0.387029628544155	0.095242814518283	0.167704904953312
Leptotrichia buccalis	Periodontitis.severity	Severe	0.583667739976209	0.353221701591619	0.098736251243169	0.172975913570312
Prevotella conceptionensis	Smoking.status	Current	-1.03704372105645	0.629002247009113	0.099492400345236	0.17351642960342
Alloprevotella rava	Smoking.status	Current	0.867616640961296	0.526322841363448	0.099546275364968	0.17351642960342
F0058 sp000163695	Smoking.status	Previous	-0.510697780119931	0.312990104837368	0.103034695227284	0.179145751500704
Neisseria elongata	Periodontitis.severity	Severe	0.826518511544069	0.509706548324849	0.10518494913231	0.181873171531736
Fusobacterium nucleatum	Periodontitis.severity	Mild/Moderate	0.772833256807214	0.476883007890103	0.10539182338761	0.181873171531736
Leptotrichia A sp001274535	Periodontitis.severity	Mild/Moderate	0.728750991489259	0.449670679321297	0.105383306617854	0.181873171531736
Capnocytophaga leadbetteri	Periodontitis.severity	Mild/Moderate	0.870602836755357	0.546484018595204	0.11142565860797	0.191540900390101
Lancefieldella rimae	Periodontitis.severity	Mild/Moderate	0.734595967760181	0.461268884160081	0.111547663088455	0.191540900390101
Aggregatibacter aphrophilus	Periodontitis.severity	Severe	0.820204687489592	0.517381745388876	0.113186128073997	0.193873268879222
F0058 sp000163695	Periodontitis.severity	Mild/Moderate	0.919983181481932	0.583527215362174	0.115177680869354	0.195834309731775
Filifactor alocis	Periodontitis.severity	Mild/Moderate	0.924546172995067	0.585870159749402	0.114836498445917	0.195834309731775
Burkholderia sp018375725	Smoking.status	Previous	-0.353914741268648	0.224482588678159	0.115180005868255	0.195834309731775
Porphyromonas pasteri	Periodontitis.severity	Severe	-0.82097692089238	0.52553137458005	0.118532445500314	0.201040324230925
Lachnoanaerobaculum saburreum	Smoking.status	Current	-0.854139711010581	0.551617593668423	0.121807366963964	0.206089726012379
Centipeda artemidis	Smoking.status	Previous	-0.386976678818707	0.251724511269791	0.124507131422367	0.210143743766532
Pauljensenia sp000185285	Periodontitis.severity	Severe	-0.716999023630892	0.467902936384392	0.125719689425262	0.211674026964189
Actinomyces oris B	Periodontitis.severity	Mild/Moderate	0.935969830160103	0.614476230860798	0.127996504961168	0.214984420954196
Dialister invisus	Smoking.status	Current	0.870069526884492	0.573152672894304	0.129291542079285	0.216633770263596
Leptotrichia buccalis	Periodontitis.severity	Mild/Moderate	0.5067876975329	0.334336691460444	0.129857383576025	0.217056302982148
Prevotella conceptionensis	Periodontitis.severity	Mild/Moderate	0.833191200651613	0.554549001448366	0.133263989083199	0.22167952030186
Fusobacterium nucleatum	Smoking.status	Previous	-0.381965476720251	0.255788691093084	0.13564956128352	0.225106706014859
Bacteroides D sp013333835	Smoking.status	Previous	-0.425108149787038	0.285276810395662	0.13646904770663	0.225924834959302
Kingella B oralis	Smoking.status	Current	-0.759494766807164	0.515574365515829	0.141009277088926	0.232884056671926
Peptidiphaga sp000466165	Periodontitis.severity	Severe	-0.852374976045104	0.579747783368956	0.141779910096694	0.233279447325229
Fusobacterium nucleatum J	Smoking.status	Previous	0.303854949533101	0.206742956208171	0.141922900757112	0.233279447325229
Aggregatibacter aphrophilus	Smoking.status	Current	-0.815263396976558	0.555469263999176	0.142471285288753	0.233625899099093
Centipeda noxia	Periodontitis.severity	Severe	0.742497355289147	0.50842525504042	0.144469699053876	0.236342864646058
Pauljensenia meyeri	Periodontitis.severity	Mild/Moderate	0.628369629662392	0.432498078119664	0.146541360201829	0.239166559574683

Eikenella exigua	Periodontitis.severity	Mild/Moderate	0.6474422445377	0.446452548618812	0.147290102118165	0.239822942742988
Filifactor alocis	Smoking.status	Current	0.96079824022261	0.664528555592889	0.148508758735412	0.240838761206517
Campylobacter B gracilis	Periodontitis.severity	Mild/Moderate	0.529530495864492	0.366337076463729	0.14861004484853	0.240838761206517
Mogibacterium timidum	Smoking.status	Previous	0.380552667249319	0.265960348843711	0.152753922368661	0.246975967941854

Supplementary Table 4: Significant associations between metadata and microbial taxa identified by maaslin2 analysis (adjusted model)

species	metadata	value	coef	stderr	pval	qval
Mogibacterium.timidum	Smoke_Perio	Former.Sever	5.883283014889	0.7560773941519	1.6612557476E-14	2.641396638686E-11
Corynebacterium.matruchotii	Teeth.number	Teeth.numbe	0.938853208875	0.1223870915375	3.77119875744E-14	2.998103012168E-11
Mogibacterium.timidum	Smoke_Perio	Current.Sever	7.519620934785	1.0222013462691	3.71521318212E-13	1.969062986521E-10
Peptidiphaga.sp000466165	Teeth.number	Teeth.numbe	1.03165818426	0.1481433669923	5.71584379889E-12	2.27204791006E-09
Treponema.B.denticola	Smoke_Perio	Never.Severe	5.787062324414	0.869242422841	4.40612339941E-11	1.401147241011E-08
Mogibacterium.timidum	Smoke_Perio	Never.Severe	5.067288329337	0.7796260968474	1.22368272183E-10	3.242759212852E-08
Actinomyces.massiliensis	Teeth.number	Teeth.numbe	0.803013747497	0.1382628405847	8.29840341033E-09	1.884923060346E-06
Tannerella.forsythia	Smoke_Perio	Never.Severe	6.004895556488	1.0718371282294	2.67662429136E-08	5.319790779078E-06
Tannerella.forsythia	Smoke_Perio	Current.Sever	7.694691716711	1.4053318121183	5.42021697337E-08	9.075657012919E-06
Tannerella.forsythia	Smoke_Perio	Former.Sever	5.681634267581	1.0394621551844	5.70796038548E-08	9.075657012919E-06
Bacteroides.D.sp905373475	Smoke_Perio	Never.Severe	4.489922554112	0.8560505595232	1.87800364483E-07	2.7145689048E-05
Rothia.dentocariosa	Smoke_Perio	Never.Severe	-3.480721725625	0.6860770766664	4.59285068674E-07	6.085527159927E-05
Treponema.B.denticola	Smoke_Perio	Former.Sever	4.221721931829	0.8429868479333	6.41108836481E-07	7.841254230807E-05
Aggregatibacter.sp00046633!	Teeth.number	Teeth.numbe	0.685434507117	0.1378907264162	7.73894214852E-07	8.378601929248E-05
Pauljensenia.sp000278725	Smoke_Perio	Former.Sever	-2.982755983177	0.6005559092406	7.90434144269E-07	8.378601929248E-05
Porphyromonas.gingivalis	Smoke_Perio	Former.Sever	3.925322689486	0.8069029085932	1.31533694581E-06	0.000125259021582
F0058.sp905372605	Smoke_Perio	Never.Severe	3.323500763121	0.6837022044223	1.33924740056E-06	0.000125259021582
Treponema.sp010365865	Smoke_Perio	Never.Severe	4.329318504999	0.8980700134734	1.63371268768E-06	0.000144311287412
Prevotella.intermedia	Smoke_Perio	Never.Severe	3.850407153516	0.8067309105549	2.06395496415E-06	0.000172720441736
Capnocytophaga.ochracea	Teeth.number	Teeth.numbe	0.608302889514	0.1297083604907	3.0822905989E-06	0.000245042102613
Porphyromonas.endodontalis	Smoke_Perio	Never.Severe	4.955715322998	1.0686760052901	3.95910418619E-06	0.000299760745526
Corynebacterium.durum	Teeth.number	Teeth.numbe	0.759363470596	0.1722723157871	1.14699479139E-05	0.000828964417416
Prevotella.buccae	Smoke_Perio	Former.Sever	3.081359271399	0.7081104238468	1.4791381463E-05	0.00098907546906
Saccharimonas.sp905372095	Smoke_Perio	Never.Severe	1.828721628403	0.4204488358031	1.49294410424E-05	0.00098907546906
Parvimonas.micra	Smoke_Perio	Former.Sever	2.926717642511	0.6749605596344	1.58464792613E-05	0.001007836081018
Streptococcus.constellatus	Smoke_Perio	Current.No Pe	8.121909720499	1.8787076338408	1.6792797746E-05	0.001026944169853
Rothia.dentocariosa	Smoke_Perio	Former.Sever	-2.857512197782	0.6653540336976	1.90532069663E-05	0.00112202218801 6
Treponema.B.denticola	Smoke_Perio	Current.Sever	4.863059097864	1.1397011701575	2.15404856938E-05	0.001223191866182
Actinomyces.sp000220835	Teeth.number	Teeth.numbe	0.724063433537	0.1700153284582	2.2327202239E-05	0.001224146605518
Porphyromonas.gingivalis	Smoke_Perio	Never.Severe	3.533237138087	0.8320346171267	2.35680548111E-05	0.001249106904989
Capnocytophaga.granulosa	Teeth.number	Teeth.numbe	0.572703933679	0.1355369777393	2.58444681273E-05	0.001284147010075
Mogibacterium.timidum	Smoke_Perio	Current.Mild/	4.32730403314	1.024050338913	2.5818405043E-05	0.001284147010075
SDRW01.sp007845485	Teeth.number	Teeth.numbe	0.608399693628	0.1448762474548	2.89445754518E-05	0.001353584557891
Streptococcus.constellatus	Smoke_Perio	Current.Sever	3.636335455725	0.8648824336639	2.83242098136E-05	0.001353584557891
Dialister.invisus	Smoke_Perio	Former.Sever	3.173384463817	0.7734280400793	4.38242487027E-05	0.001990873012493
Lautropia.dentalis	Teeth.number	Teeth.numbe	0.520583675854	0.1279953242695	5.10111687344E-05	0.002252993285767

Dialister.invisus	Smoke_Perio	Never.Severe	3.230977078842	0.7975171440692	5.45527060579E-05	0.00234429196303
Porphyromonas.endodontalis	Smoke_Perio	Former.Sever	4.175842787471	1.0363965143545	5.98597380589E-05	0.00237942458784
Streptococcus.constellatus	Smoke_Perio	Former.Sever	2.580556854837	0.6397155111164	5.86870097189E-05	0.00237942458784
Johnsonella.sp900766185	Teeth.number	Teeth.numbe	0.422069768517	0.1044957280659	5.74207140683E-05	0.00237942458784
Bulleidia.moorei	Smoke_Perio	Former.Sever	2.640206238137	0.6598078860056	6.72088572551E-05	0.00260639226916
Anaeroglobus.geminatus	Smoke_Perio	Former.Sever	3.074115391031	0.7734178221603	7.51100613575E-05	0.002843452322819
Pauljensenia.sp000278725	Smoke_Perio	Never.Mild/M	-2.256496999605	0.5685561797178	7.69650202217E-05	0.002845915864013
Pauljensenia.sp000278725	Smoke_Perio	Current.Sever	-3.206391110536	0.8119394438769	8.35066619905E-05	0.003017627103748
F0058.sp905372605	Smoke_Perio	Former.Sever	2.604398872408	0.6630508947634	9.10868075249E-05	0.003218400532545
Arachnia.sp905372155	Teeth.number	Teeth.numbe	0.597222275205	0.1537243268321	0.000108496918967	0.003750219590389
Pauljensenia.sp000278725	Smoke_Perio	Never.Severe	-2.402420612544	0.6192607570089	0.000110955695302	0.003753607564467
Porphyromonas.sp000467855	Teeth.number	Teeth.numbe	0.496678502167	0.1285751447372	0.000118643646805	0.003930070800427
Rothia.mucilaginosa.A	Teeth.number	Teeth.numbe	-0.409550573124	0.1070436498157	0.000137644362364	0.004466419105287
Fusobacterium.vincentii	Smoke_Perio	Current.Sever	3.607145593259	0.9546404600839	0.000166305758613	0.005288523123881
Pauljensenia.sp000278725	Smoke_Perio	Former.Mild/N	-2.13619399602	0.5707640180624	0.000191576602964	0.00597268232771
Pauljensenia.hongkongensis	Teeth.number	Teeth.numbe	0.488951248565	0.1314476134757	0.000209608335587	0.006409177953516
Prevotella.buccae	Smoke_Perio	Never.Severe	2.705081149786	0.730165152603	0.000222235020002	0.006543586700071
Bacteroides.D.sp905373475	Smoke_Perio	Former.Sever	3.076495204921	0.8301934464788	0.000221366951064	0.006543586700071
Porphyromonas.pasteri	Teeth.number	Teeth.numbe	0.511939382236	0.1384393668148	0.000228213770454	0.006597452636753
Gemella.morbillorum	Teeth.number	Teeth.numbe	0.460574245168	0.1259640857014	0.000268017861309	0.00760979284788
Prevotella.intermedia	Smoke_Perio	Current.Sever	3.802561781338	1.057739634654	0.000338945434021	0.009454793685839
Gemella.sanguinis	Smoke_Perio	Former.Sever	-2.157157724955	0.6037663071313	0.000368595746261	0.010104607526815
F0058.sp000163695	Smoke_Perio	Current.Sever	4.355125298889	1.2250576478219	0.0003941591364	0.010622254692823
Gemella.sanguinis	Smoke_Perio	Never.Severe	-2.196867326223	0.6225711455964	0.00043504320923	0.011528645044607
Capnocytophaga.gingivalis	Teeth.number	Teeth.numbe	0.466547507711	0.1330451606718	0.000472227672828	0.012110354835424
Neisseria.sp000090875	Smoke_Perio	Current.Sever	-3.854733343098	1.098756804692	0.000469458423447	0.012110354835424
SDRK01.sp007845205	Smoke_Perio	Never.Severe	2.267634112496	0.6513746601727	0.000518771634339	0.013092807914267
Parvimonas.micra	Smoke_Perio	Current.Sever	3.162029626459	0.9125330264777	0.000550668625207	0.01368067365748
Prevotella.buccae	Smoke_Perio	Current.Sever	3.297334538459	0.9573509724825	0.000594512146772	0.014542681744121
Lautropia.mirabilis	Smoke_Perio	Current.Sever	-3.996345619838	1.162096635265	0.000606183765052	0.014603517976248
Corynebacterium.matruchotii	Smoke_Perio	Former.Sever	-2.323156035641	0.6795147585477	0.000652173311059	0.015476948725121
Rothia.aeria	Smoke_Perio	Current.Sever	-4.181094625138	1.2317057956404	0.000712191456673	0.016652712001614
Actinomyces.massiliensis	Smoke_Perio	Current.Sever	-3.4841939931	1.0378604200562	0.000814912846463	0.018778426461965
Alloprevotella.sp905369775	Education	Unknown	4.946807826952	1.4873809272454	0.000910929611278	0.020463663315407
Peptidiphaga.gingivicola	Teeth.number	Teeth.numbe	0.34743924202	0.104547837242	0.000919360427562	0.020463663315407
Cardiobacterium.hominis	Teeth.number	Teeth.numbe	0.4818104865	0.1450787171707	0.000926656452018	0.020463663315407
Lautropia.mirabilis	Teeth.number	Teeth.numbe	0.51221982069	0.1548134784993	0.000968260349318	0.021081681985579
Prevotella.intermedia	Smoke_Perio	Former.Sever	2.585617706035	0.7823635036085	0.000981160042096	0.021081681985579

Anaeroglobus.geminatus	Smoke_Perio	Never.Severe	2.609659774742	0.797506607904	0.001100391131289	0.023328291983322
Prevotella.histicola	Smoke_Perio	Former.Sever	-2.16635113322	0.6647157849369	0.001152407124945	0.02410957011399
F0058.sp000163695	Smoke_Perio	Never.Severe	3.017045066388	0.9343432346968	0.001279143726533	0.026413487340105
Saccharimonas.sp905372095	Smoke_Perio	Former.Sever	1.310786763626	0.4077491267079	0.001344263946017	0.027402303514971
Corynebacterium.matruchotii	Smoke_Perio	Never.Severe	-2.232235719526	0.7006788498828	0.001484500678406	0.029877925046408
Prevotella.histicola	Smoke_Perio	Never.Severe	-2.174186946321	0.6854189490806	0.001555999102391	0.030925482160014
Anaeroglobus.geminatus	Smoke_Perio	Current.Sever	3.308499044708	1.0456452542501	0.001598903347666	0.031362421676288
Parvimonas.micra	Smoke_Perio	Never.Severe	2.199784444171	0.6959828063951	0.001617433067582	0.031362421676288
Prevotella.denticola	Smoke_Perio	Former.Sever	2.961718015209	0.9385777581352	0.001645992641064	0.031531666256522
Streptococcus.constellatus	Smoke_Perio	Never.Severe	2.068962671169	0.6596400195033	0.001755643398549	0.033231821472536
Prevotella.loescheii	Teeth.number	Teeth.number	0.486000255896	0.1557952915022	0.001859356076726	0.034780896023471
Leptotrichia.A.sp000469505	Teeth.number	Teeth.number	0.31885245974	0.1029349342365	0.002000857409472	0.036992596291409
Bulleidia.moorei	Smoke_Perio	Never.Severe	2.100585051495	0.6803581892733	0.002069788620584	0.037707982874998
Peptostreptococcus.stomatis	Smoke_Perio	Former.Sever	1.863715849995	0.6045379300939	0.002101714289089	0.037707982874998
Alloprevotella.tannerae	Smoke_Perio	Current.Sever	3.985859514776	1.2934401020158	0.0021106984125	0.037707982874998
Arachnia.rubra	Smoke_Perio	Current.Sever	-3.686032729699	1.2051022480338	0.002277441532834	0.039792659749508
Gemella.sanguinis	Smoke_Perio	Current.Sever	-2.498691200502	0.8162798368992	0.002259548366577	0.039792659749508
Treponema.sp010365865	Smoke_Perio	Former.Sever	2.649309821721	0.8709436976246	0.00240735692014	0.041605407641547
Corynebacterium.matruchotii	Smoke_Perio	Current.Sever	-2.789037872112	0.918690211306	0.00245535713223	0.041788337414525
Corynebacterium.durum	Smoke_Perio	Current.Sever	-3.923435891512	1.2931501860566	0.002470505482368	0.041788337414525
F0058.sp000163695	Diabetes_status	Diabetes	-2.065766140668	0.6851755797648	0.002629865115991	0.04401563720448
Porphyromonas.endodontalis	Smoke_Perio	Current.Sever	4.217466785593	1.4011871277147	0.002673353537121	0.044277417958575
Dialister.invisus	Smoke_Perio	Current.Sever	3.119572943572	1.0456590686699	0.002914662356442	0.047776424193216
Stomatobaculum.longum	Education	Unknown	5.045070672794	1.6979881210913	0.003031522251765	0.049184901839862

Supplementary Table 5:Network properties of microbial taxa associated with smoking and periodontitis.

Taxon	Degree	Strength	Betweenness
Capnocytophaga.periodontitic	4	6.4825	0.0003
Metamycoplasma.salivarium	4	5.7232	0
Mogibacterium.timidum	3	9.6582	0.217
Fretibacterium.fastidiosum	3	9.5387	0
CAJPSE01.sp905373185	3	7.5065	0
Fusobacterium.nucleatum.J	3	7.2525	0
CAJPQU01.sp905373705	3	7.2267	0
Treponema.D.sp014334325	3	6.6685	0
Slackia.exigua	3	6.3904	0
Actinomyces.israelii	3	6.0689	0
Corynebacterium.durum	3	6.0581	0
Actinomyces.naeslundii	3	5.8196	0
Actinomyces.sp000195595	3	5.593	0
Gallibacter.brachus	3	5.5579	0
Tannerella.serpentiformis	3	5.2103	0
Actinomyces.massiliensis	3	5.1468	0
Prevotella.buccae	3	5.0562	0
Capnocytophaga.granulosa	3	4.9855	0
Nanoperiomorbus.sp9053722	3	4.9665	0
Prevotella.saccharolytica	3	4.721	0
Olsenella.F.sp001189515	3	4.6436	0
Campylobacter.A.sp90537274	3	4.4072	0
Actinomyces.johnsonii	3	4.3119	0
Centipeda.infelix	3	4.2203	0
Centipeda.hMGS.04979	3	4.1909	0
Actinomyces.oris.E	3	3.9871	0
Johnsonella.sp900766185	3	3.243	0
Actinomyces.oris.D	3	2.9983	0
Treponema.C.sp905372025	2	8.1232	0.0065
Campylobacter.A.rectus	2	7.2989	0
Selenomonas.sp905373085	2	7.1635	0
Treponema.B.denticola	2	6.5465	0
Treponema.D.buccale	2	6.2855	0
CAJPSE01.hMGS.02407	2	6.2128	0
Treponema.D.hMGS.03080	2	6.0501	0
Catonella.morbi	2	5.9033	0

Lautropia.mirabilis	2	5.6315	0
CAJPQU01.sp905372875	2	5.5281	0
Allisonella.pneumosinta	2	5.4274	0
Treponema.sp010365865	2	5.3489	0
Pseudoramibacter.alactolyticu	2	5.3011	0
Prevotella.oralis	2	5.1241	0
Selenomonas.sp905372355	2	4.865	0
Olsenella.uli	2	4.7622	0
Parvimonas.micra	2	4.7406	0
Cryptobacteroides.hMGS.0309	2	4.729	0
Lautropia.dentalis	2	4.6668	0.0082
Centipeda.hMGS.05237	2	4.6518	0
Selenomonas.sputigena	2	4.645	0
Rothia.dentocariosa	2	4.579	0
Arachnia.rubra	2	4.4623	0
Neisseria.sp000090875	2	4.3635	0
Bacteroides.D.sp905373475	2	4.2107	0
Anaeroglobus.geminatus	2	4.1955	0
Eubacterium.B.infirmum	2	4.1837	0
Nanoperiomorbus.sp9053732	2	4.137	0.0287
Centipeda.periodontii	2	4.0457	0
Prevotella.seregens	2	3.9136	0
Fusobacterium.vincentii	2	3.7927	0
Aggregatibacter.sp00046633	2	3.7836	0
Centipeda.sp001683335	2	3.6424	0
Tannerella.sp002890585	2	3.5874	0
Cardiobacterium.hMGS.03071	2	3.5287	0
Bulleidia.moorei	2	3.5233	0
Cardiobacterium.hMGS.03122	2	3.4518	0
Peptoanaerobacter.yurii	2	3.4194	0
Cardiobacterium.sp91661874	2	3.3651	0
Saccharimonas.sp018127705	2	3.3541	0
Treponema.D.paredis	2	3.3325	0
Prevotella.maculosa	2	3.3223	0
Prevotella.conceptionensis	2	3.3048	0
Actinomyces.oris	2	3.2737	0
Campylobacter.B.hMGS.0311	2	3.1595	0
Neisseria.elongata	2	3.1476	0

Nanoperiomorbus.sp9053733	2	3.0947	0
Centipeda.sp905372865	2	2.9655	0
Lancefieldella.rimae	2	2.9503	0
Catonella.hMGS.05203	2	2.9459	0
Actinomyces.sp001278845	2	2.9389	0
Actinomyces.dentalis	2	2.8898	0
Catonella.hMGS.05016	2	2.8659	0
Centipeda.flueggei	2	2.847	0
Streptococcus.gordonii	2	2.7244	0
Arachnia.propionica	2	2.7044	0
Capnocytophaga.leadbetteri	2	2.4665	0
Gemella.morbillorum	2	2.4049	0
SDRW01.sp007845485	2	2.4004	0
Centipeda.sp001717585	2	2.3968	0
Nanoperiomorbus.periodontic	2	2.3592	0
Porphyromonas.catoniae	2	2.3247	0
JABCPE02.hMGS.05228	2	2.302	0
Corynebacterium.matruchotii	2	2.2529	0
Fusobacterium.hMGS.06114	2	2.1608	0
Granulicatella.adiacens	2	2.1123	0
Campylobacter.B.hMGS.0522	2	1.9239	0
Pauljensenia.hongkongensis	2	1.8982	0
Actinomyces.gerencseriae	2	1.8036	0
Leptotrichia.hofstadii	2	1.7664	0
Leptotrichia.buccalis	2	1.6058	0
Tannerella.forsythia	1	6.3339	0
Desulfobulbus.oralis	1	4.9659	0
Porphyromonas.endodontalis	1	4.8592	0
CAJPNN01.sp905372035	1	4.2268	0
Filifactor.alocis	1	4.1682	0
Rothia.aeria	1	3.5831	0
Hornefia.nodata	1	3.487	0
Dialister.invisus	1	3.297	0
F0058.sp000163695	1	3.2864	0
Alloprevotella.tannerae	1	3.2198	0
Prevotella.pleuritidis	1	3.1386	0
Alloprevotella.rava	1	3.0985	0
F0058.sp905372605	1	2.8652	0

Bacteroides.D.sp013333835	1	2.8373	0
Cardiobacterium.hominis	1	2.8088	0
Saccharimonas.sp905371715	1	2.7951	0
Prevotella.denticola	1	2.6443	0
Prevotella.oris	1	2.6214	0
Prevotella.sp013333935	1	2.5709	0
Actinomyces.sp002999235	1	2.5596	0
Fusobacterium.nucleatum	1	2.5307	0
Oribacterium.sp000160135	1	2.2792	0
Treponema.C.lecithinolyticum	1	2.2687	0
Prevotella.nigrescens	1	1.9792	0
Actinomyces.sp915069725	1	1.9508	0
Arachnia.sp905372155	1	1.8692	0
Peptidiphaga.sp000466165	1	1.8643	0
Eikenella.exigua	1	1.7876	0
Fusobacterium.animalis	1	1.7368	0
Campylobacter.B.hMGS.0524	1	1.6955	0
Capnocytophaga.sputigena	1	1.6739	0
Prevotella.oulorum	1	1.6451	0
Nanosynbacter.hMGS.03124	1	1.6402	0
Propionivibrio.hMGS.03093	1	1.6377	0
Streptococcus.intermedius	1	1.6244	0
Campylobacter.B.hMGS.0522	1	1.6237	0
Streptococcus.mutans	1	1.5211	0
Actinomyces.oris.A	1	1.4894	0
Porphyromonas.pasteri	1	1.4655	0
Centipeda.artemidis	1	1.4302	0
Burkholderia.sp018375725	1	1.3677	0
Eikenella.corrodens	1	1.3177	0
Capnocytophaga.gingivalis	1	1.3155	0
Johnsonella.ignava	1	1.3134	0
Kingella.B.oralis	1	1.2587	0
Centipeda.timonae	1	1.2453	0
Centipeda.felix	1	1.2433	0
Pauljensenia.meyeri	1	1.2167	0
Campylobacter.B.gracilis.A	1	1.1698	0
Campylobacter.B.sp90537321	1	1.1621	0
Campylobacter.B.sp90537329	1	1.0337	0

Actinomyces.sp000220835	1	1.0071	0
Campylobacter.B.sp9005395(	1	0.9831	0
Lachnoanaerobaculum.saburr	1	0.7488	0
Veillonella.sp900757715	1	0.6035	0
Severe	122	323.8399	0.8341
Current	65	126.8142	0.349
Former	50	40.517	0.038
Mild/Moderate	46	61.7191	0.0011

Supplementary Table 6:Pathways associated with smoking status and periodontitis severity.

Pathway	metadata	value	coef	stderr	pval	qval
tryptophan biosynthesis chori	Periodontitis.severit	Severe	-1.147959870331	0.1094675394727	1.37572173982E-24	6.988666438293E-22
pentose phosphate pathway c	Periodontitis.severit	Severe	-0.94534671032	0.0924371774683	1.63344768447E-23	2.765971412361E-21
isoleucine biosynthesis threo	Periodontitis.severit	Severe	-0.91173856012	0.0889301436061	1.2854779604E-23	2.765971412361E-21
pyrimidine deoxyribonuleotide	Periodontitis.severit	Severe	-1.475276148747	0.1464719535343	6.96708110586E-23	8.848193004447E-21
nitrogen fixation nitrogen am	Periodontitis.severit	Severe	4.994928141301	0.5095838902719	8.37874229073E-22	8.512802167386E-20
cysteine biosynthesis serine c	Periodontitis.severit	Severe	-0.73637431911	0.0765281530766	4.25098838016E-21	3.599170161873E-19
valine isoleucine biosynthesis	Periodontitis.severit	Severe	-0.853736418991	0.0903665457001	2.01439333328E-20	1.461874019012E-18
heme biosynthesis glutamate	Periodontitis.severit	Severe	-0.863576251682	0.0926281205909	6.01057752909E-20	3.816716730971E-18
benzoate degradation benzoa	Periodontitis.severit	Severe	-4.308719115041	0.4781242181726	8.78476836944E-19	4.95851370186E-17
polyamine biosynthesis argini	Periodontitis.severit	Severe	4.740219578541	0.5280725702345	1.18457425381E-18	6.017637209367E-17
fatty acid biosynthesis initiati	Periodontitis.severit	Severe	-0.616619022247	0.0696467974162	3.33342149571E-18	1.539434654383E-16
threonine biosynthesis aspart	Periodontitis.severit	Severe	-0.575209883161	0.0651100632093	3.91022314493E-18	1.655327798021E-16
beta oxidation	Periodontitis.severit	Severe	-4.25802773075	0.4982549852731	4.21004652665E-17	1.645156642722E-15
dissimilatory sulfate reductior	Periodontitis.severit	Severe	5.594835207187	0.6574029467002	5.60787722776E-17	2.03485830836E-15
f420 biosynthesis	Periodontitis.severit	Severe	6.160188918577	0.7299401704599	9.9569788217E-17	3.372096827615E-15
catechol ortho cleavage catec	Periodontitis.severit	Severe	-4.005802507248	0.4756347256022	1.14395368966E-16	3.632052964675E-15
lysine biosynthesis mediated	Periodontitis.severit	Severe	5.406641706769	0.6454171834742	1.64033058949E-16	4.629377441445E-15
ornithine biosynthesis mediat	Periodontitis.severit	Severe	5.406641706769	0.6454171834742	1.64033058949E-16	4.629377441445E-15
prpp biosynthesis ribose 5p p	Periodontitis.severit	Severe	0.388502590651	0.0469311983939	3.59661328314E-16	9.616208146495E-15
glycolysis embden meyerhof p	Periodontitis.severit	Severe	0.208392437426	0.0253283868206	5.35531481602E-16	1.360249963268E-14
c5 isoprenoid biosynthesis n	Periodontitis.severit	Severe	0.304601222721	0.037698057236	1.69368765752E-15	4.097111095327E-14
gluconeogenesis oxaloacetate	Periodontitis.severit	Severe	0.305938057843	0.0379710686958	2.02153197712E-15	4.667901110794E-14
menaquinone biosynthesis ch	Periodontitis.severit	Severe	-0.503537400737	0.0629681627739	3.21880765069E-15	7.109366463262E-14
denitrification nitrate nitrogen	Periodontitis.severit	Severe	-1.603925661265	0.2016998907667	4.53027182548E-15	9.589075363932E-14
c10 c20 isoprenoid biosynthes	Periodontitis.severit	Severe	-1.115903578852	0.1414152251889	7.21001162602E-15	1.465074362407E-13
nucleotide sugar biosynthesis	Periodontitis.severit	Severe	-0.816537902602	0.1038065848708	8.71409422931E-15	1.70259994942E-13
lysine biosynthesis succinyl d	Periodontitis.severit	Severe	-0.425219909335	0.054752262097	1.8446627043E-14	3.470698717712E-13
assimilatory sulfate reduction	Periodontitis.severit	Severe	-1.956090696212	0.2528524633683	2.31059907221E-14	4.192086888152E-13
pyrimidine degradation uracil	Periodontitis.severit	Severe	1.873917777221	0.242911321856	2.71548067014E-14	4.756773035962E-13
glycolysis core module involv	Periodontitis.severit	Severe	0.230428580785	0.030218652333	5.24783914615E-14	8.88634095414E-13
phosphatidylethanolamine pe	Periodontitis.severit	Severe	1.285843652736	0.1688489646145	5.64970778968E-14	9.258230829537E-13
biotin biosynthesis biow pathw	Periodontitis.severit	Severe	1.432391811421	0.1891282835009	7.67152969415E-14	1.217855338947E-12
ubiquinone biosynthesis proka	Smoking.status	Current	-3.11101553935	0.4126487839344	9.87140224257E-14	1.474903629185E-12
anthranilate degradation anth	Smoking.status	Current	-4.818670707881	0.6390520374797	9.78505172892E-14	1.474903629185E-12
biotin biosynthesis bioi pathw	Periodontitis.severit	Severe	1.418720317591	0.1891462838049	1.30604078094E-13	1.895624904909E-12
nucleotide sugar biosynthesis	Periodontitis.severit	Severe	-0.732467427999	0.0977854303756	1.40524781575E-13	1.982960806664E-12

nad biosynthesis aspartate na	Periodontitis.severit	Severe	1.84394604769	0.2478279318515	2.01891337479E-13	2.771913498357E-12
biotin biosynthesis pimeloyl a	Periodontitis.severit	Severe	1.408338329348	0.1894113347953	2.0942554582E-13	2.799688875694E-12
lipopolysaccharide biosynthes	Periodontitis.severit	Severe	1.21588745959	0.164187085022	2.59549943233E-13	3.380804388776E-12
leucine degradation leucine a	Periodontitis.severit	Severe	-3.382977299652	0.4572402498966	2.72553175926E-13	3.461425334255E-12
assimilatory nitrate reduction	Periodontitis.severit	Severe	3.696474673567	0.5002890609417	2.92824380199E-13	3.628165491246E-12
riboflavin biosynthesis gtp rib	Periodontitis.severit	Severe	0.544810332597	0.0748335063386	6.33310716577E-13	7.66004390527E-12
c10 c20 isoprenoid biosynthes	Periodontitis.severit	Severe	-1.270981660368	0.1748024991086	6.76533485032E-13	7.992535125495E-12
cmp kdo biosynthesis	Periodontitis.severit	Severe	1.190759299708	0.16628346959	1.46325022819E-12	1.689388899815E-11
tyrosine biosynthesis prephan	Periodontitis.severit	Severe	-2.839167056956	0.4034535176928	3.44680781589E-12	3.891063045496E-11
histidine degradation histidi	Periodontitis.severit	Severe	1.016329139572	0.1452582627733	4.54694730896E-12	5.021411375977E-11
pimeloyl acp biosynthesis bio	Periodontitis.severit	Severe	1.059962262058	0.1541097473539	1.01690330357E-11	1.09912101748E-10
d galactonate degradation de	Periodontitis.severit	Severe	4.312256224555	0.6284441451116	1.13367169734E-11	1.19980254635E-10
pyruvate oxidation pyruvate a	Periodontitis.severit	Severe	0.156349567588	0.0228007153143	1.16902477613E-11	1.21196854342E-10
lysine degradation lysine sac	Periodontitis.severit	Severe	-3.791100304938	0.5552304316114	1.42196900421E-11	1.444720508274E-10
purine degradation xanthine u	Periodontitis.severit	Severe	-2.857235559206	0.4220719033175	2.09838442219E-11	2.090155463676E-10
d galacturonate degradation I	Periodontitis.severit	Severe	2.342876123631	0.3497300797095	3.34150215712E-11	3.264390568874E-10
fatty acid biosynthesis elonga	Periodontitis.severit	Severe	0.151525653454	0.0229779676023	6.6193703509E-11	6.344604034449E-10
citrate cycle tca cycle krebs c	Periodontitis.severit	Severe	-0.791567778985	0.1226655748773	1.64049693255E-10	1.543282299507E-09
triacylglycerol biosynthesis	Periodontitis.severit	Severe	-2.526284116502	0.3918943993889	1.71206223314E-10	1.581322935341E-09
cam crassulacean acid metab	Periodontitis.severit	Severe	-1.819352800579	0.2851843678807	2.61180186878E-10	2.369277409532E-09
cam crassulacean acid metab	Periodontitis.severit	Severe	2.55391428496	0.4012029104199	2.85087281798E-10	2.54077787988E-09
pentose phosphate pathway p	Periodontitis.severit	Severe	-0.292648045149	0.0460495128392	3.04648794438E-10	2.668303234047E-09
histidine biosynthesis prpp his	Periodontitis.severit	Severe	-0.569639691783	0.0898924945828	3.41399067495E-10	2.93950383538E-09
pentose phosphate pathway r	Periodontitis.severit	Severe	0.178858152414	0.0294888204022	1.80927536687E-09	1.531853143954E-08
semi phosphorylative entner c	Periodontitis.severit	Severe	1.16084922762	0.1916249236609	1.89123406333E-09	1.574994924869E-08
malonate semialdehyde pathw	Periodontitis.severit	Severe	-2.448644394494	0.405331686207	2.09249181176E-09	1.71449329093E-08
benzene degradation benzene	Periodontitis.severit	Severe	-2.441880033285	0.4057948412352	2.40963858596E-09	1.943010161378E-08
beta oxidation acyl coa synthe	Periodontitis.severit	Severe	0.158052798538	0.0262839640572	2.47155358993E-09	1.961795662011E-08
shikimate pathway phosphoer	Periodontitis.severit	Severe	0.133653249614	0.0222396828861	2.52534015834E-09	1.973650462207E-08
betaine biosynthesis choline b	Periodontitis.severit	Severe	-1.424511533932	0.2394941299601	3.64359294936E-09	2.804462451933E-08
pyridoxal biosynthesis erythrc	Periodontitis.severit	Severe	2.525619045404	0.4318324593277	6.53200841489E-09	4.952627275765E-08
uridine monophosphate biosy	Periodontitis.severit	Severe	0.202518302112	0.0350900984669	1.02189656315E-08	7.634168442386E-08
glucuronate pathway uronate	Periodontitis.severit	Severe	2.479273122119	0.4329981694328	1.32730489504E-08	9.772041835969E-08
trehalose biosynthesis d gluc	Periodontitis.severit	Severe	0.381962809838	0.067038074285	1.55846535422E-08	1.131000571347E-07
proline biosynthesis glutamat	Periodontitis.severit	Severe	-0.433936679597	0.0764794365044	1.78424374436E-08	1.276613833994E-07
glyoxylate cycle	Periodontitis.severit	Severe	-2.421584968795	0.4312255344365	2.48023031354E-08	1.749940276773E-07
tyrosine degradation tyrosine	Periodontitis.severit	Severe	-2.676845987419	0.4776842683697	2.65004886057E-08	1.81922273131E-07
thiamine biosynthesis air thia	Periodontitis.severit	Severe	1.197306506441	0.2138606730605	2.7294424114E-08	1.848742326655E-07

entner doudoroff pathway glu	Periodontitis.severit	Severe	1.042990413359	0.1873008671735	3.22824887996E-08	2.157829514496E-07
pantothenate biosynthesis val	Periodontitis.severit	Severe	0.598747459962	0.1083601260361	4.10026047014E-08	2.705106907573E-07
adenine ribonucleotide biosyn	Periodontitis.severit	Severe	0.13998898878	0.0259522319929	8.42944312526E-08	5.489945009782E-07
glutathione biosynthesis glut	Smoking.status	Current	-1.878758882828	0.3507472878762	1.03332491192E-07	6.561613190715E-07
dissimilatory nitrate reductio	Periodontitis.severit	Severe	-0.812105052367	0.1515487660154	1.02081739678E-07	6.561613190715E-07
citrate cycle first carbon oxid	Periodontitis.severit	Severe	-0.503525603716	0.0947397668632	1.29222243392E-07	8.084634014246E-07
guanine ribonucleotide biosyn	Periodontitis.severit	Severe	0.102041069089	0.0192059583695	1.30499997868E-07	8.084634014246E-07
trans cinnamate degradation	Periodontitis.severit	Severe	-2.823191059511	0.5368558010116	1.74224352572E-07	1.066337001281E-06
cobalamin biosynthesis cobin	Periodontitis.severit	Severe	0.562658879943	0.1090034077403	2.90050633364E-07	1.754115735106E-06
gaba gamma aminobutyrate s	Periodontitis.severit	Severe	-2.649752361646	0.5500024218426	1.65561984517E-06	9.779707922612E-06
isoleucine biosynthesis pyruv	Periodontitis.severit	Severe	-0.34934706665	0.0732966435512	2.1290363652E-06	1.243161463819E-05
lysine biosynthesis dap amino	Periodontitis.severit	Severe	-0.166471881485	0.0359182915801	4.00196699377E-06	2.310226400951E-05
homoprotocatechuate degrad	Periodontitis.severit	Severe	-1.446559328773	0.3128175735769	4.20497966935E-06	2.400145698912E-05
citrate cycle second carbon o	Smoking.status	Current	-0.151098779114	0.0350582136798	1.7793635659E-05	9.719534316939E-05
lysine biosynthesis acetyl dap	Periodontitis.severit	Severe	-0.38754029777	0.0921600772097	2.8222099247E-05	0.000149341941849
pentose phosphate pathway a	Periodontitis.severit	Severe	-1.477773593799	0.354736669445	3.34538530042E-05	0.000171662195213
ornithine biosynthesis glutam	Periodontitis.severit	Severe	-0.347687044477	0.0866780471495	6.44836743066E-05	0.000321153985762
polyamine biosynthesis argini	Periodontitis.severit	Severe	0.905907786341	0.2275118033543	7.28885275826E-05	0.000356032423192
reductive pentose phosphate	Periodontitis.severit	Severe	0.113988753032	0.0297148020133	0.000132118546464	0.000627254407509
galactose degradation leloir	Periodontitis.severit	Severe	0.178690594649	0.0479859403422	0.000206196579037	0.000960989561019
urea cycle	Periodontitis.severit	Severe	0.649642935161	0.1803356407109	0.000329377759769	0.001507422540203
tryptophan metabolism trypt	Periodontitis.severit	Severe	-1.471162364838	0.4123174329517	0.000375176671588	0.00170169418899
leucine biosynthesis 2 oxoiso	Periodontitis.severit	Severe	-0.231780533174	0.0670901546659	0.000571790431513	0.002504047751799
reductive citrate cycle arnon	Periodontitis.severit	Severe	0.525875523691	0.1573429680594	0.000859086000584	0.003606741225592
coenzyme a biosynthesis pant	Smoking.status	Current	0.089104688139	0.0269278833886	0.000966628193897	0.004020576970151
d glucuronate degradation d g	Periodontitis.severit	Severe	0.602950403653	0.186654879932	0.001273282816083	0.005118096624562
catechol meta cleavage catec	Periodontitis.severit	Severe	-0.95791223546	0.2964905520656	0.001270901784624	0.005118096624562
lysine biosynthesis dap dehyd	Periodontitis.severit	Severe	-0.073666502409	0.0233250470542	0.00163041433588	0.006420546376953
cysteine biosynthesis homocy	Smoking.status	Current	-1.257853810154	0.4355383656762	0.003952460760935	0.014341786189678
c1 unit interconversion prokar	Periodontitis.severit	Severe	0.125465375736	0.0438249861755	0.004277906182209	0.015304058736354
undecaprenylphosphate alpha	Smoking.status	Current	1.196206758684	0.4261100288316	0.005084643939757	0.017813787044115
cysteine biosynthesis methior	Periodontitis.severit	Severe	1.040898247479	0.3810617721131	0.006404212031936	0.021545296107439
propanoyl coa metabolism pro	Smoking.status	Current	-1.101854018431	0.4324272974817	0.010967334925125	0.03460500709294
methionine salvage pathway	Periodontitis.severit	Severe	0.856261730172	0.3388373464647	0.011641628524044	0.036505847470457
phosphate acetyltransferase a	Periodontitis.severit	Severe	0.066174825764	0.0267681543097	0.013580972736212	0.041812934242397
formaldehyde assimilation ri	Periodontitis.severit	Mild/Moderate	0.621527498342	0.2540454507125	0.014580107972442	0.043826596745567
methionine degradation	Periodontitis.severit	Severe	1.074590838516	0.443829643035	0.015631419979451	0.045900354621739

Supplementary Table 7: Per-sample quality control metrics for shotgun metagenomic sequencing of subgingival plaque samples.						
**shotgun_file**	**Low quality**	**Host**	**Unmapped**	**Mapped**	**Total (read pairs)**	**Total (bases)**
BERHUSFR22703531	18330	26118501	306847	866490	27310168	8193050400
BERHUSFR22703532	44822	16623111	1396604	4025425	22089962	6626988600
BERHUSFR22703538	13003	22792495	130911	310296	23246705	6974011500
BERHUSFR22703547B	6035	21730953	68951	261285	22067224	6620167200
BERHUSFR22703548	78201	29075079	428980	1677852	31260112	9378033600
BERHUSFR22703555	19738	24826721	83952	462584	25392995	7617898500
BERHUSFR22703573	94821	39506164	878995	1713611	42193591	12658077300
BERHUSFR22703584	7329	22200414	123894	303489	22635126	6790537800
BERHUSFR22703590	18499	22647914	39717	80947	22787077	6836123100
BERHUSFR22703595	25162	20076613	461732	1070151	21633658	6490097400
BERHUSFR22703597	165873	22749094	596878	1469295	24981140	7494342000
BERHUSFR22703608	7897	23945574	20993	34923	24009387	7202816100
BERHUSFR22703610	58959	20730801	200123	653535	21643418	6493025400
BERHUSFR22703629	60515	29042708	229741	516051	29849015	8954704500
BERHUSFR22703641	30987	21904166	177764	528738	22641655	6792496500
BERHUSFR22703647	12952	26125469	72294	238671	26449386	7934815800
BERHUSFR22703659	162663	58117604	1618170	3144387	63042824	18912847200
BERHUSFR22703661B	6207	23416333	91651	132999	23647190	7094157000
BERHUSFR22703668	64062	26924231	613367	1181483	28783143	8634942900
BERHUSFR22703679	54188	25437130	1450095	3517998	30459411	9137823300
BERHUSFR22703705	14608	28940672	37266	76481	29069027	8720708100
BERHUSFR22703713	50174	24188696	238464	571851	25049185	7514755500
BERHUSFR22703719	111381	41223588	536660	1029040	42900669	12870200700
BERHUSFR22703723	63361	25388483	722954	1835099	28009897	8402969100
BERHUSFR22703724	80056	24786326	369768	1059397	26295547	7888664100
BERHUSFR22703725	67378	34822053	675949	1369809	36935189	11080556700
BERHUSFR22703726	150769	53423928	1207839	2831889	57614425	17284327500
BERHUSFR22703728	22084	21510815	94932	143656	21771487	6531446100
BERHUSFR22703732	99296	30344934	687200	2043761	33175191	9952557300
BERHUSFR22703733	32391	27339719	387272	771829	28531211	8559363300
BERHUSFR22703736	26906	34336908	132789	174764	34671367	10401410100
BERHUSFR22703738	17882	28051527	202972	587114	28859495	8657848500
BERHUSFR22703740	9326	24003085	39392	125018	24176821	7253046300
BERHUSFR22703741	22994	35121920	126222	206969	35478105	10643431500
BERHUSFR22703743	13014	19215179	37138	77268	19342599	5802779700
BERHUSFR22703746	13163	21674142	84049	210836	21982190	6594657000

BERHUSFR22703749	178713	46648401	1650287	5395344	53872745	16161823500
BERHUSFR22703752	50380	35151618	250888	596279	36049165	10814749500
BERHUSFR22703755	23215	24100789	486509	1461940	26072453	7821735900
BERHUSFR22703756	2810	6034353	69406	94691	6201260	1860378000
BERHUSFR22703760	33381	37218645	59861	99143	37411030	11223309000
BERHUSFR22703762	31340	27976454	242204	469954	28719952	8615985600
BERHUSFR22703763	33881	28176444	147387	254143	28611855	8583556500
BERHUSFR22703764	22981	25214412	57285	59141	25353819	7606145700
BERHUSFR22703766	60341	29805728	720200	1207144	31793413	9538023900
BERHUSFR22703767	142230	26311897	994947	2256431	29705505	8911651500
BERHUSFR22703771	8555	21669811	60958	159623	21898947	6569684100
BERHUSFR22703773	17927	23513680	157372	471369	24160348	7248104400
BERHUSFR22703775	12955	27549657	102627	235307	27900546	8370163800
BERHUSFR22703777	46905	33469661	596042	1425028	35537636	10661290800
BERHUSFR22703778	49163	41570290	393758	985854	42999065	12899719500
BERHUSFR22703781	41498	20814703	221943	379142	21457286	6437185800
BERHUSFR22703782	35199	21040223	629271	2187456	23892149	7167644700
BERHUSFR22703783	18591	22175835	89773	224002	22508201	6752460300
BERHUSFR22703784	71631	31164903	623574	2043023	33903131	10170939300
BERHUSFR22703787	72502	33895872	758631	1788247	36515252	10954575600
BERHUSFR22703790	15540	21507244	31666	50514	21604964	6481489200
BERHUSFR22703791	217373	30426101	1057527	2847452	34548453	10364535900
BERHUSFR22703796	13691	21852495	321884	688736	22876806	6863041800
BERHUSFR22703799	46730	32879341	87463	231106	33244640	9973392000
BERHUSFR22703800	38276	23608349	218804	527786	24393215	7317964500
BERHUSFR22703801	401241	184622375	3166564	5300516	193490696	58047208800
BERHUSFR22703802	32274	41837012	100369	20580	41990235	12597070500
BERHUSFR22703805	19455	24264286	165099	427086	24875926	7462777800
BERHUSFR22703808	25836	28106274	155322	278895	28566327	8569898100
BERHUSFR22703809	17892	62471233	122446	20890	62632461	18789738300
BERHUSFR22703811	115283	38840015	851604	2212636	42019538	12605861400
BERHUSFR22703813	27061	45159653	129030	347095	45662839	13698851700
BERHUSFR22703816	25294	25960340	46259	92356	26124249	7837274700
BERHUSFR22703817	109381	22568249	577610	1428322	24683562	7405068600
BERHUSFR22703818	37211	33324987	201674	327289	33891161	10167348300
BERHUSFR22703819	89802	43279940	574799	1554305	45498846	13649653800
BERHUSFR22703820	55911	40639510	347010	1075027	42117458	12635237400
BERHUSFR22703822	16648	25206459	99631	295633	25618371	7685511300

BERHUSFR22703824	44542	22731026	305077	1065400	24146045	7243813500
BERHUSFR22703827	5649	2031659	79041	209522	2325871	697761300
BERHUSFR22703834	20480	32600744	123135	179706	32924065	9877219500
BERHUSFR22703835	72394	36524188	563721	1173394	38333697	11500109100
BERHUSFR22703850	10012	26338209	135070	316912	26800203	8040060900
BERHUSFR22703851	10185	24479699	89557	244224	24823665	7447099500
BERHUSFR22703855	13419	23529562	88487	224451	23855919	7156775700
BERHUSFR22703857	27029	42868722	174782	335075	43405608	13021682400
BERHUSFR22703858	13642	22241169	58486	111478	22424775	6727432500
BERHUSFR22703859	16300	21120353	157798	493688	21788139	6536441700
BERHUSFR22703861	22108	30410466	186098	460133	31078805	9323641500
BERHUSFR22703864	331027	25402139	1581502	5462017	32776685	9833005500
BERHUSFR22703866	18122	31015310	84254	72100	31189786	9356935800
BERHUSFR22703867	67524	23240551	505341	1306522	25119938	7535981400
BERHUSFR22703869	394144	94770963	2796263	9611530	107572900	32271870000
BERHUSFR22703870B	9733	21257101	199237	899512	22365583	6709674900
BERHUSFR22703873	20135	20783873	252898	786147	21843053	6552915900
BERHUSFR22703876	63981	26194600	505416	1547911	28311908	8493572400
BERHUSFR22703877	7329	12454877	18793	33010	12514009	3754202700
BERHUSFR22703884	130959	117106446	1262347	4377408	122877160	36863148000
BERHUSFR22703886	13919	23701495	77754	173054	23966222	7189866600
BERHUSFR22703888	21870	21878012	136512	389260	22425654	6727696200
BERHUSFR22703889	27958	25314985	482243	1201788	27026974	8108092200
BERHUSFR22703890	2526	5499781	36306	76170	5614783	1684434900
BERHUSFR22703891	16209	20629092	258591	806839	21710731	6513219300
BERHUSFR22703892	21093	24118292	170345	503234	24812964	7443889200
BERHUSFR22703895	86674	114750395	1231207	2856329	118924605	35677381500
BERHUSFR22703896	20718	23132807	167012	657057	23977594	7193278200
BERHUSFR22703897	9019	22467200	118896	310095	22905210	6871563000
BERHUSFR22703899	51209	20432306	311004	634152	21428671	6428601300
BERHUSFR22703900	12181	24009288	34576	60988	24117033	7235109900
BERHUSFR22703901	46456	20470344	287933	905713	21710446	6513133800
BERHUSFR22703903	25413	26030041	267266	733635	27056355	8116906500
BERHUSFR22703905	40205	20291184	530360	1738339	22600088	6780026400
BERHUSFR22703908	28609	24712779	172878	508434	25422700	7626810000
BERHUSFR22703909	36328	48246668	313943	761430	49358369	14807510700
BERHUSFR22703910	3609	22122301	15214	25029	22166153	6649845900
BERHUSFR22703913	10017	22134156	22269	32282	22198724	6659617200

BERHUSFR22703914	7321	23325635	19838	39119	23391913	7017573900
BERHUSFR22704107	114142	18886296	606106	1645616	21252160	6375648000
BERHUSFR22704110	44126	32735959	270829	655205	33706119	10111835700
BERHUSFR22704114	76719	31387959	276561	640386	32381625	9714487500
BERHUSFR22704115	6231	3014636	46099	97759	3164725	949417500
BERHUSFR22704116	78906	25420802	561613	1619097	27680418	8304125400
BERHUSFR22704119	34862	34674392	272730	1197378	36179362	10853808600
BERHUSFR22704120	130130	22568071	1589331	3566760	27854292	8356287600
BERHUSFR22704122	115161	29483421	494337	842380	30935299	9280589700
BERHUSFR22704125	64163	24022204	500213	755967	25342547	7602764100
BERHUSFR22704126	69215	22677197	360990	1078404	24185806	7255741800
BERHUSFR22704128	70818	24368415	369042	1135069	25943344	7783003200
BERHUSFR22704129	13169	26706344	76355	163362	26959230	8087769000
BERHUSFR22704130	46028	24802268	379790	982321	26210407	7863122100
BERHUSFR22704131	16241	32713119	55625	125286	32910271	9873081300
BERHUSFR22704134	33461	28535418	133066	479212	29181157	8754347100
BERHUSFR22704137	182592	25452445	918162	1872291	28425490	8527647000
BERHUSFR22704139	166274	22230287	628165	1611992	24636718	7391015400
BERHUSFR22704144	51151	21854184	265672	692347	22863354	6859006200
BERHUSFR22704146	423062	19494233	4815445	16620672	41353412	12406023600
BERHUSFR22704147	74786	33062324	485220	1448455	35070785	10521235500
BERHUSFR22704149	14257	23545750	83923	305548	23949478	7184843400
BERHUSFR22704150	31588	32182920	333810	876662	33424980	10027494000
BERHUSFR22704153	74194	34919037	482306	1071562	36547099	10964129700
BERHUSFR22704154	135438	20228403	699376	1761804	22825021	6847506300
BERHUSFR22704155	19273	28569972	40798	67401	28697444	8609233200
BERHUSFR22704157	41810	24146680	214069	487750	24890309	7467092700
BERHUSFR22704159	19241	32786142	41063	68736	32915182	9874554600
BERHUSFR22704161	9469	25580507	23413	30869	25644258	7693277400
BERHUSFR22704163	2210	2134148	15314	27633	2179305	653791500
BERHUSFR22704164	44348	26916221	239418	550460	27750447	8325134100
BERHUSFR22704165	59482	42643491	501073	1130078	44334124	13300237200
BERHUSFR22704166	22207	21059872	333577	939831	22355487	6706646100
BERHUSFR22704168	45166	33959189	358456	1106600	35469411	10640823300
BERHUSFR22704169	28373	23905837	250662	652499	24837371	7451211300
BERHUSFR22704171	93584	22943833	291217	844019	24172653	7251795900
BERHUSFR22704172	110551	31654646	709485	2644510	35119192	10535757600
BERHUSFR22704173	54536	29786436	435578	1314345	31590895	9477268500

BERHUSFR22704174	34631	24291762	161399	414265	24902057	7470617100
BERHUSFR22704175	82186	20250965	518836	1382835	22234822	6670446600
BERHUSFR22704176	117333	33330587	829994	3873112	38151026	11445307800
BERHUSFR22704178	19598	30323884	65745	181145	30590372	9177111600
BERHUSFR22704179	117831	40860779	304439	714442	41997491	12599247300
BERHUSFR22704181	28348	24697909	234664	310098	25271019	7581305700
BERHUSFR22704182	31368	25133215	233160	670553	26068296	7820488800
BERHUSFR22704183	55329	20322200	881717	2927693	24186939	7256081700
BERHUSFR22704184	61093	32368637	341607	994955	33766292	10129887600
BERHUSFR22704185	95945	31571489	629144	1723470	34020048	10206014400
BERHUSFR22704186	64193	25341312	336703	639714	26381922	7914576600
BERHUSFR22704189	75198	22161417	468646	1056407	23761668	7128500400
BERHUSFR22704190	147980	21628004	861141	1976028	24613153	7383945900
BERHUSFR22704191	41120	33003016	328166	939656	34311958	10293587400
BERHUSFR22704193	15203	27549879	173967	542106	28281155	8484346500
BERHUSFR22704194	47971	22625493	308107	1050747	24032318	7209695400
BERHUSFR22704195	51763	23973521	229193	587450	24841927	7452578100
BERHUSFR22704196	207413	18497779	1323381	3688097	23716670	7115001000
BERHUSFR22704197	49634	24981666	420275	1087769	26539344	7961803200
BERHUSFR22704198	16871	23006532	119464	341375	23484242	7045272600
BERHUSFR22704200	22939	19279434	130398	318272	19751043	5925312900
BERHUSFR22704201	104051	25221061	700547	1780858	27806517	8341955100
BERHUSFR22704202	52805	33931917	418652	1124957	35528331	10658499300
BERHUSFR22704203	100571	28888081	750332	1457169	31196153	9358845900
BERHUSFR22704207	46190	39345186	272178	693643	40357197	12107159100
BERHUSFR22704208	29696	29922633	161576	505736	30619641	9185892300
BERHUSFR22704210	173790	32539258	1091694	2947569	36752311	11025693300
BERHUSFR22704215	47705	20923283	291570	791743	22054301	6616290300
BERHUSFR22704218	10492	23905188	42008	103516	24061204	7218361200
BERHUSFR22704224	15801	29283729	17130	2934	29319594	8795878200
BERHUSFR22704228	22234	29125511	93438	128731	29369914	8810974200
BERHUSFR22704244	24086	22424892	355206	822771	23626955	7088086500
BERHUSFR22704257	431034	138977428	5740951	16166746	161316159	48394847700
BERHUSFR22704259	23958	32576466	456098	1350395	34406917	10322075100
BERHUSFR22704260	33634	26292401	105404	141505	26572944	7971883200
BERHUSFR22704263	19957	20113732	344304	1025334	21503327	6450998100
BERHUSFR22704267	33873	24793373	200272	419788	25447306	7634191800
BERHUSFR22704273	75702	34983340	433990	1066800	36559832	10967949600

BERHUSFR22704274	74579	22601456	433075	927662	24036772	7211031600
BERHUSFR22704276	19431	20312381	213122	449648	20994582	6298374600
BERHUSFR22704282	101985	22355653	703487	1297707	24458832	7337649600
BERHUSFR22704284	133625	23372620	988229	3837538	28332012	8499603600
BERHUSFR22704285	15823	21961129	29989	55036	22061977	6618593100
BERHUSFR22704287	6701	7018229	323665	840347	8188942	2456682600
BERHUSFR22704292	27905	24840326	219064	703517	25790812	7737243600
BERHUSFR22704298	16331	21629249	203641	589846	22439067	6731720100
BERHUSFR22704299	20764	25047660	202957	543652	25815033	7744509900
BERHUSFR22704301	19991	29695743	74174	111457	29901365	8970409500
BERHUSFR22704323	28598	28739438	154086	357266	29279388	8783816400
BERHUSFR22704325	26113	30706442	118438	312925	31163918	9349175400
BERHUSFR22704329	104792	32777313	692047	1859920	35434072	10630221600
BERHUSFR22704332	110901	19946670	364965	833450	21255986	6376795800
BERHUSFR22704337	3419	3639261	115347	334196	4092223	1227666900
BERHUSFR22704354	43120	39147174	413653	1165343	40769290	12230787000
BERHUSFR22704360	71137	28036764	532910	1209659	29850470	8955141000
BERHUSFR22704363	70357	27891441	488056	1194267	29644121	8893236300
BERHUSFR22704369	8160	3023936	42287	93560	3167943	950382900
BERHUSFR22704370	88711	21750802	1003683	3056539	25899735	7769920500
BERHUSFR22704374	139484	20204185	1156973	3515366	25016008	7504802400
BERHUSFR22704375	60623	27595206	268451	747776	28672056	8601616800
BERHUSFR22704380	65504	39799577	518856	2011546	42395483	12718644900
BERHUSFR22704385B	19267	45549002	293652	746723	46608644	13982593200
BERHUSFR22704392	142048	20345123	2287803	5894668	28669642	8600892600
BERHUSFR22704393	11407	23549914	32746	78235	23672302	7101690600
BERHUSFR22704491	22955	24212629	194521	576753	25006858	7502057400
BERHUSFR22704492	16382	20603416	140462	358013	21118273	6335481900
BERHUSFR22704496	105846	23068683	486380	1448538	25109447	7532834100
BERHUSFR22704498	23868	40394246	148334	255611	40822059	12246617700
BERHUSFR22704500	19723	24490513	103829	386377	25000442	7500132600
BERHUSFR22704501	141129	31648437	1187075	2608508	35585149	10675544700
BERHUSFR22704503	42641	21682685	402489	1030162	23157977	6947393100
BERHUSFR22704504	54161	19836640	510237	2142823	22543861	6763158300
BERHUSFR22704507	41977	30163179	292951	700793	31198900	9359670000
BERHUSFR22704508	184459	38632842	1269167	3083705	43170173	12951051900
BERHUSFR22704509	27844	19534612	380154	1690580	21633190	6489957000
BERHUSFR22704513	70731	32484717	1049939	2421834	36027221	10808166300

BERHUSFR22704514	44605	26892068	250006	515474	27702153	8310645900
BERHUSFR22704515	70474	29098405	402537	736346	30307762	9092328600
BERHUSFR22704571	32535	13003463	466112	1243705	14745815	4423744500
BERHUSFR22704575	31293	16700671	22165	35029	16789158	5036747400
BERHUSFR22704577	23428	33836859	26297	28062	33914646	10174393800
BERHUSFR22704578	44932	31710658	52971	115192	31923753	9577125900
BERHUSFR22704580	15939	24168033	54327	107755	24346054	7303816200
BERHUSFR22704582	32418	25654164	231702	715323	26633607	7990082100
BERHUSFR22704584	73665	27970449	431324	1188045	29663483	8899044900
BERHUSFR22704585	32134	29439664	279693	841678	30593169	9177950700
BERHUSFR22704586	35479	22408436	471890	1948621	24864426	7459327800
BERHUSFR22704587	21818	21090819	281876	1190757	22585270	6775581000
BERHUSFR22704588	114958	16754240	1366952	4086210	22322360	6696708000
BERHUSFR22704592	47306	22251258	394037	1389996	24082597	7224779100
BERHUSFR22704593	42667	20977276	316768	802948	22139659	6641897700
BERHUSFR22704595	20844	27982955	209159	593911	28806869	8642060700
BERHUSFR22704597	484046	8867059	3146280	9060690	21558075	6467422500
BERHUSFR22704599	22317	42472890	741817	1819695	45056719	13517015700
BERHUSFR22704600	12373	62822337	180851	526986	63542547	19062764100
BERHUSFR22704601	17768	22727038	331303	770139	23846248	7153874400
BERHUSFR22704602	24531	21004847	443769	1715561	23188708	6956612400
BERHUSFR22704603	10055	22600257	44655	69546	22724513	6817353900
BERHUSFR22704604	18984	30964473	215214	556820	31755491	9526647300
BERHUSFR22704605	15029	21366192	313063	677865	22372149	6711644700
BERHUSFR22704606	25218	27029916	218924	672794	27946852	8384055600
BERHUSFR22704608	88019	18093572	889481	2497813	21568885	6470665500
BERHUSFR22704611	6928	35643181	152964	561788	36364861	10909458300
BERHUSFR22704614	13238	22599246	175692	457949	23246125	6973837500
BERHUSFR22704616	45070	20836370	810731	2085686	23777857	7133357100
BERHUSFR22704617	30824	118345665	1089084	3148300	122613873	36784161900
BERHUSFR22704618B	7357	26437555	239201	879431	27563544	8269063200
BERHUSFR22704619	30616	23789440	415638	1576212	25811906	7743571800
BERHUSFR22704622	27945	22050114	322942	773071	23174072	6952221600
BERHUSFR22704623	15601	36630693	657241	1695741	38999276	11699782800
BERHUSFR22704624	58726	26664941	375940	782285	27881892	8364567600
BERHUSFR22704625	34860	24374168	545981	1564750	26519759	7955927700
BERHUSFR22704628	3456	26641483	10785	7989	26663713	7999113900
BERHUSFR22704631	79996	30448340	609407	2096477	33234220	9970266000

BERHUSFR22704632	15018	20514695	338529	927000	21795242	6538572600
BERHUSFR22704635	25022	19413557	645367	1684302	21768248	6530474400
BERHUSFR22704638	11802	25589122	13968	11910	25626802	7688040600
BERHUSFR22704639	54099	21285063	263935	865216	22468313	6740493900
BERHUSFR22704642	40143	26149498	321517	1149977	27661135	8298340500
BERHUSFR22704646	14078	23881536	41535	74385	24011534	7203460200
BERHUSFR22704647	117164	29519279	1419740	5411764	36467947	10940384100
BERHUSFR22704650	25836	21431645	251793	1070882	22780156	6834046800
BERHUSFR22704652	76084	24312406	592513	1979251	26960254	8088076200
BERHUSFR22704654	25790	30463831	326399	1267688	32083708	9625112400
BERHUSFR22704655	27189	21478293	210125	627981	22343588	6703076400
BERHUSFR22704656	4943	24191929	19860	34945	24251677	7275503100
BERHUSFR22704658	7919	21586857	20064	31610	21646450	6493935000
BERHUSFR22704659	18764	25222950	151424	392514	25785652	7735695600
BERHUSFR22704660	42239	22819086	234333	721438	23817096	7145128800
BERHUSFR22704661	21436	24825691	168552	542691	25558370	7667511000
BERHUSFR22704663	8026	24032755	31415	70627	24142823	7242846900
BERHUSFR22704665	9434	28481289	129562	316950	28937235	8681170500
BERHUSFR22704667	94975	12414142	1304605	3433209	17246931	5174079300
BERHUSFR22704669	38255	23591035	344287	924859	24898436	7469530800
BERHUSFR22704672	97729	26151073	1122732	3065168	30436702	9131010600
BERHUSFR22704673	7674	31599340	103138	253839	31963991	9589197300
BERHUSFR22704677	37710	21919805	380923	1506489	23844927	7153478100
BERHUSFR22704681	12952	24536536	30159	96208	24675855	7402756500
BERHUSFR22704875	18235	25051072	85119	191928	25346354	7603906200
BERHUSFR22704878	203273	165035851	3433204	14812629	183484957	55045487100
BERHUSFR22704879	28386	25643238	185182	497268	26354074	7906222200
BERHUSFR22704880	36481	21822592	116744	224563	22200380	6660114000
BERHUSFR22704882	25860	20433711	491087	1275929	22226587	6667976100
BERHUSFR22704884	54365	22906738	425969	740118	24127190	7238157000
BERHUSFR22704885	67465	18973813	551707	1898944	21491929	6447578700
BERHUSFR22704892	62476	21744330	293080	619932	22719818	6815945400
BERHUSFR22704893	18105	24619360	87530	176746	24901741	7470522300
BERHUSFR22704895	24148	23545765	146738	383957	24100608	7230182400
BERHUSFR22704896	19290	31070307	114487	187224	31391308	9417392400
BERHUSFR22704898	44023	55718646	307615	862217	56932501	17079750300
BERHUSFR22704901	61916	73386539	508584	1350390	75307429	22592228700
BERHUSFR22704902	53450	98100949	153003	346624	98654026	29596207800

BERHUSFR22704904	13502	22614822	218932	452397	23299653	6989895900
BERHUSFR22704905	19039	28027498	116983	134367	28297887	8489366100
BERHUSFR22704906	19820	24911686	120305	280854	25332665	7599799500
BERHUSFR22704907	4235	22637646	35635	93897	22771413	6831423900
BERHUSFR22704909	57820	21357668	477089	1544301	23436878	7031063400
BERHUSFR22704911	54040	33304749	560831	1592965	35512585	10653775500
BERHUSFR22704913	47039	31645234	186398	305163	32183834	9655150200
BERHUSFR22704915	4992	16681510	115143	348027	17149672	5144901600
BERHUSFR22704917	19800	26143820	159603	631371	26954594	8086378200
BERHUSFR22704919	59066	19577597	580099	2135526	22352288	6705686400
BERHUSFR22704920	12811	24319999	42074	99715	24474599	7342379700
BERHUSFR22704922	120609	17633457	1038782	2679320	21472168	6441650400
BERHUSFR22704925	37604	24465537	620309	2541657	27665107	8299532100
BERHUSFR22704926	16264	20720310	148506	526994	21412074	6423622200
BERHUSFR22704928	24322	21347899	199395	635845	22207461	6662238300
BERHUSFR22704929	16196	23453403	222255	639554	24331408	7299422400
BERHUSFR22704931	13242	21335236	117790	336595	21802863	6540858900
BERHUSFR22704934B	12602	23343849	524867	1795204	25676522	7702956600
BERHUSFR22704935	157314	26753014	917633	2510894	30338855	9101656500
BERHUSFR22704937	19298	21392586	178289	488492	22078665	6623599500
BERHUSFR22704939	64121	23351264	507572	1185181	25108138	7532441400
BERHUSFR22704940	22785	22854061	127600	305201	23309647	6992894100
BERHUSFR22704942	5611	22854244	63217	173299	23096371	6928911300
BERHUSFR22704945	49182	22799725	378461	1714309	24941677	7482503100
BERHUSFR22704947	15947	21086813	58626	129401	21290787	6387236100
BERHUSFR22704948	28048	20144317	529344	1239386	21941095	6582328500
BERHUSFR22704950B	13234	13603718	33156	59528	13709636	4112890800
BERHUSFR22704952	10885	28171356	256602	684483	29123326	8736997800
BERHUSFR22704955	59620	18634304	574730	2111417	21380071	6414021300
BERHUSFR22704956	151971	32187128	1598502	5213386	39150987	11745296100
BERHUSFR22704957	34755	23212977	282974	707888	24238594	7271578200
BERHUSFR22704958	6785	20995188	98209	190238	21290420	6387126000
BERHUSFR22704960	17551	24641253	119052	354778	25132634	7539790200
BERHUSFR22704966	32706	27274392	285482	582310	28174890	8452467000
BERHUSFR22704967B	3312	21788171	67389	187919	22046791	6614037300
BERHUSFR22704968	134876	25364238	1515015	4808469	31822598	9546779400
BERHUSFR22704969	59016	64654866	399746	911161	66024789	19807436700
BERHUSFR22704970	7475	22273183	25565	35957	22342180	6702654000

BERHUSFR22707853	49865	23543756	532553	1758011	25884185	7765255500
BERHUSFR22707855	16516	59716477	101373	97655	59932021	17979606300
BERHUSFR22707856	94154	29441804	566840	1526880	31629678	9488903400
BERHUSFR22707857	56377	23442581	401537	1472436	25372931	7611879300
BERHUSFR22707861B	5994	26509883	129539	325513	26970929	8091278700
BERHUSFR22707864	48167	26690380	901524	2752169	30392240	9117672000
BERHUSFR22707865	19540	24305840	156453	442421	24924254	7477276200
BERHUSFR22707869	24375	24964675	188509	492686	25670245	7701073500
BERHUSFR22707870B	4966	22335084	77439	194430	22611919	6783575700
BERHUSFR22707872	47100	36026519	577591	1631171	38282381	11484714300
BERHUSFR22707874	30908	24969992	282379	825865	26109144	7832743200
BERHUSFR22707875	23787	23377781	209238	544360	24155166	7246549800
BERHUSFR22707876	45844	22251988	502568	1518861	24319261	7295778300
BERHUSFR22707878	19078	47292372	283623	749684	48344757	14503427100
BERHUSFR22707880	10886	30119006	177195	542475	30849562	9254868600
BERHUSFR22707884	2769	5423121	94851	216155	5736896	1721068800
BERHUSFR22707887	80764	20313487	467080	1602258	22463589	6739076700
BERHUSFR22707889	12758	19646043	415483	1262986	21337270	6401181000
BERHUSFR22707890	8812	21063525	69445	126417	21268199	6380459700
BERHUSFR22707891	12187	23670806	154848	372851	24210692	7263207600
BERHUSFR22707895	5982	23796640	46879	124999	23974500	7192350000
BERHUSFR22707898	12142	21837314	45805	101398	21996659	6598997700
BERHUSFR22707899	11023	21795210	27446	45217	21878896	6563668800
BERHUSFR22707900	23603	32566927	461847	1617783	34670160	10401048000
BERHUSFR22707901	8305	21432780	159033	271570	21871688	6561506400
BERHUSFR22707902	21459	18927690	598465	1938710	21486324	6445897200
BERHUSFR22707904	33042	28899291	438706	1410637	30781676	9234502800
BERHUSFR22707906	40323	19937716	298165	970571	21246775	6374032500
BERHUSFR22707909	25584	24274698	119328	337175	24756785	7427035500
BERHUSFR22707910	210192	22858392	1674096	4006700	28749380	8624814000
BERHUSFR22707911	14918	24953797	297909	669053	25935677	7780703100
BERHUSFR22707913	18308	24424227	43365	103892	24589792	7376937600
BERHUSFR22707914	41946	22920360	265406	880663	24108375	7232512500
BERHUSFR22707915	6799	21454344	15303	8851	21485297	6445589100
BERHUSFR22707916	81166	26910767	475100	1541493	29008526	8702557800
BERHUSFR22707918	50336	23824776	419927	819022	25114061	7534218300
BERHUSFR22707921	111548	19400634	674277	2462329	22648788	6794636400
BERHUSFR22707922	45810	29889034	269810	425847	30630501	9189150300

BERHUSFR22707925	24083	28211260	90478	196810	28522631	8556789300
BERHUSFR22707928	73321	29075795	637130	1744846	31531092	9459327600
BERHUSFR22707929	17806	21422245	903579	2870755	25214385	7564315500
BERHUSFR22707931	40261	65481501	526857	1562868	67611487	20283446100
BERHUSFR22707932	13221	8013035	179814	331609	8537679	2561303700
BERHUSFR22707933	17546	23663635	134265	208997	24024443	7207332900
BERHUSFR22707934	19541	25518505	123874	323866	25985786	7795735800
BERHUSFR22707939	32897	32702698	209919	514585	33460099	10038029700
BERHUSFR22707940	58219	25756823	377720	860713	27053475	8116042500
BERHUSFR22707943	52516	26854306	969873	1515212	29391907	8817572100
BERHUSFR22707944	9279	26899064	51401	81662	27041406	8112421800
BERHUSFR22707946	12039	22794172	186159	387725	23380095	7014028500
BERHUSFR22707957	46077	49005793	991823	1944236	51987929	15596378700
BERHUSFR22707958	12498	20963051	171498	361075	21508122	6452436600
BERHUSFR22707972	35954	17747475	150439	239693	18173561	5452068300
BERHUSFR22707978	14570	6860093	142324	393049	7410036	2223010800
BERHUSFR22707992	93940	33554756	432032	1084418	35165146	10549543800
BERHUSFR22707993B	5187	21765488	65890	201043	22037608	6611282400
BERHUSFR22707997	17463	6355309	167969	572393	7113134	2133940200
BERHUSFR22707999	74632	43934733	360093	738239	45107697	13532309100
BERHUSFR22708000	30756	24835876	132967	328031	25327630	7598289000
BERHUSFR22708002B	13314	20978246	586773	1793941	23372274	7011682200
BERHUSFR22708007	130044	41218623	1629712	4129623	47108002	14132400600
BERHUSFR22708015	609379	133046735	4226983	14296280	152179377	45653813100
BERHUSFR22708019	28066	20320693	338192	742796	21429747	6428924100
BERHUSFR22708020	36042	23238019	175480	348292	23797833	7139349900
BERHUSFR22708023	7279	9070690	58017	124359	9260345	2778103500
BERHUSFR22708025	6908	5680260	10326	19898	5717392	1715217600
BERHUSFR22708027	40678	19274963	686216	2236219	22238076	6671422800
BERHUSFR22708030	28871	18800956	1454452	4358305	24642584	7392775200
BERHUSFR22708032	12892	18993129	64205	102777	19173003	5751900900
BERHUSFR22708033	56327	20535040	130207	268905	20990479	6297143700
BERHUSFR22708037	139087	37854417	802339	2004491	40800334	12240100200
BERHUSFR22708141	41322	21393011	164900	511673	22110906	6633271800
BERHUSFR22708153	40878	19567033	981589	2624631	23214131	6964239300
BERHUSFR22708172	28884	21023501	719517	1567066	23338968	7001690400
BERHUSFR22708177	110275	21109076	599949	1891551	23710851	7113255300
BERHUSFR22708179	37590	12631220	604067	1399319	14672196	4401658800

BERHUSFR22708180	23694	21239259	154677	291602	21709232	6512769600
BERHUSFR22708183	34355	21530313	189324	652302	22406294	6721888200
BERHUSFR22708187	15943	22135597	75790	165209	22392539	6717761700
BERHUSFR22708188	16459	21905788	187476	363596	22473319	6741995700
BERHUSFR22708190	32447	29546619	186956	428440	30194462	9058338600
BERHUSFR22708192	22763	18712831	133664	373952	19243210	5772963000
BERHUSFR22708197	7268	12462190	83559	232926	12785943	3835782900
BERHUSFR22708198	33735	38712862	526267	999259	40272123	12081636900
BERHUSFR22708202	26708	17600945	250776	388131	18266560	5479968000
BERHUSFR22708206	62696	22933403	362384	874594	24233077	7269923100
BERHUSFR22708214	24963	22605168	121481	216482	22968094	6890428200
BERHUSFR22708215	383380	174294643	3505935	8181750	186365708	55909712400
BERHUSFR22708216	3844	4952709	22186	46704	5025443	1507632900
BERHUSFR22708217	12252	21014493	44613	101267	21172625	6351787500
BERHUSFR22708218	25424	20532091	110116	245908	20913539	6274061700
BERHUSFR22708220B	5301	24856540	34458	87239	24983538	7495061400
BERHUSFR22708222	12136	21007424	15720	4005	21039285	6311785500
BERHUSFR22708223B	5192	21304634	353164	387794	22050784	6615235200
BERHUSFR22708224	51119	19309592	363763	1285991	21010465	6303139500
BERHUSFR22708225	40469	21301202	43140	67333	21452144	6435643200
BERHUSFR22708226	81299	47852379	749236	2545659	51228573	15368571900
BERHUSFR22708228	6748	7753473	59435	121601	7941257	2382377100
BERHUSFR22708229	29573	21771611	118611	394865	22314660	6694398000
BERHUSFR22708230	21091	15335656	216845	392798	15966390	4789917000
BERHUSFR22708236	129579	101621173	2455043	3786938	107992733	32397819900
BERHUSFR22708261	35894	17066458	256277	536485	17895114	5368534200
BERHUSFR22708262B	4478	21248009	42145	128032	21422664	6426799200
BERHUSFR22708263	10470	22625422	87751	141678	22865321	6859596300
BERHUSFR22708264	71599	42633137	211610	501130	43417476	13025242800
BERHUSFR22708266	235396	18419138	1256240	4333556	24244330	7273299000
BERHUSFR22708271	86371	37082977	1124642	2767197	41061187	12318356100
BERHUSFR22708279	69536	30501001	778751	3175279	34524567	10357370100
BERHUSFR22708283	9745	5479440	43060	102109	5634354	1690306200
BERHUSFR22708287	24581	16974883	155439	461723	17616626	5284987800
BERHUSFR22708291	20943	18652710	130400	412437	19216490	5764947000
BERHUSFR22708295	6773	11455088	60698	162265	11684824	3505447200
BERHUSFR22708298	10663	7132201	75822	186943	7405629	2221688700
BERHUSFR22708306	12704	22863245	216301	641848	23734098	7120229400

BERHUSFR22708310	34937	15653550	376038	1064206	17128731	5138619300
BERHUSFR22708313	11318	5277860	81152	233515	5603845	1681153500
BERHUSFR22708320	42040	19585923	207269	480828	20316060	6094818000
BERHUSFR23638169	35910	43017501	435390	877783	44366584	13309975200
BERHUSFR23638170	12240	28003291	45794	74764	28136089	8440826700
BERHUSFR23638171	102067	27628439	715413	2318744	30764663	9229398900
BERHUSFR23638173	7236	4351977	70539	164279	4594031	1378209300
BERHUSFR23638175	66547	20567273	491116	1426387	22551323	6765396900
BERHUSFR23638176	8117	23747133	19408	13980	23788638	7136591400
BERHUSFR23638178	70699	24845486	1318317	2690814	28925316	8677594800
BERHUSFR23638179B	13558	23613218	75174	328218	24030168	7209050400
BERHUSFR23638180	44271	35040185	127593	226639	35438688	10631606400
BERHUSFR23638181	182555	69243431	1238817	3525481	74190284	22257085200
BERHUSFR23638182	128897	30780689	985328	2606274	34501188	10350356400
BERHUSFR23638184	9182	22072793	19071	24360	22125406	6637621800
BERHUSFR23638185	4282	12520187	41837	68036	12634342	3790302600
BERHUSFR23638186	49686	10757480	467108	1491060	12765334	3829600200
BERHUSFR23638188	105511	19732030	729711	2931898	23499150	7049745000
BERHUSFR23638190	92617	28837871	567242	1431606	30929336	9278800800
BERHUSFR23638191	11133	21437976	86598	267302	21803009	6540902700
BERHUSFR23638192	26366	19517559	438465	1481640	21464030	6439209000
BERHUSFR23638193	26909	51778016	236790	458086	52499801	15749940300
BERHUSFR23638194	123423	366962110	766511	1141211	368993255	110697976500
BERHUSFR23638197	12400	33036372	57092	150202	33256066	9976819800
BERHUSFR23638200	35166	27725688	994069	2383933	31138856	9341656800
BERHUSFR23638202	84621	19736892	510753	1524621	21856887	6557066100
BERHUSFR23638203	17679	22509144	51007	96785	22674615	6802384500
BERHUSFR23638206	5229	22218287	13529	4691	22241736	6672520800
BERHUSFR23638211	27953	21641944	195665	583327	22448889	6734666700
BERHUSFR23638212	79608	20009155	347769	1263863	21700395	6510118500
BERHUSFR23638214	46537	21167008	660721	1800954	23675220	7102566000
BERHUSFR23638215	66253	43994228	623628	1718156	46402265	13920679500
BERHUSFR23638217B	7911	19802864	219181	526119	20556075	6166822500
BERHUSFR23638218	40624	39552084	344534	721995	40659237	12197771100
BERHUSFR23638219B	5588	23365959	92311	290538	23754396	7126318800
BERHUSFR23638221	16793	22242652	79941	188529	22527915	6758374500
BERHUSFR23638222	19749	21860915	47012	150255	22077931	6623379300
BERHUSFR23638226	6177	22311507	18323	46734	22382741	6714822300

BERHUSFR23638228	18133	16613839	91709	66893	16790574	5037172200
BERHUSFR23638229	17457	30982291	198581	370699	31569028	9470708400
BERHUSFR23638230	60214	28748929	330615	550138	29689896	8906968800
BERHUSFR23638231B	8077	21385599	146210	486025	22025911	6607773300
BERHUSFR23638232	11012	28494867	311866	890008	29707753	8912325900
BERHUSFR23638233	18051	24354515	364373	598290	25335229	7600568700
BERHUSFR23638234	13396	9799978	89043	175681	10078098	3023429400
BERHUSFR23638237	41877	20378535	373452	1168073	21961937	6588581100
BERHUSFR23638238	78141	17693349	782676	3167707	21721873	6516561900
BERHUSFR23638239	126777	31382171	533614	2239366	34281928	10284578400
BERHUSFR23638240	7313	8832631	51719	72541	8964204	2689261200
BERHUSFR23638246	35209	54766963	683027	1399249	56884448	17065334400
BERHUSFR23638248	82818	19694558	682607	2609210	23069193	6920757900
BERHUSFR23638250	19340	21307880	172508	426008	21925736	6577720800
BERHUSFR23638251	34205	13998943	434949	998582	15466679	4640003700
BERHUSFR23638252	35186	42839605	451596	1016936	44343323	13302996900
BERHUSFR23638257	14914	22222011	274969	708026	23219920	6965976000
BERHUSFR23638258	122315	11466452	298846	564275	12451888	3735566400
BERHUSFR23638259	46123	21716805	732422	949740	23445090	7033527000
BERHUSFR23638260	12310	13572427	210992	401934	14197663	4259298900
BERHUSFR23638261	15773	21393204	268488	660494	22337959	6701387700
BERHUSFR23638262	8659	22367399	44244	103275	22523577	6757073100
BERHUSFR23638264	54503	19889056	1427675	3980708	25351942	7605582600
BERHUSFR23638265B	4465	21818729	36605	80711	21940510	6582153000
BERHUSFR23638266	12474	21848161	282889	714985	22858509	6857552700
BERHUSFR23638267	13048	23058196	69951	127904	23269099	6980729700
BERHUSFR23638268	22527	19963830	472986	973886	21433229	6429968700
BERHUSFR23638269	20433	14507058	427285	1055041	16009817	4802945100
BERHUSFR23638270	61906	21980878	551533	2503970	25098287	7529486100
BERHUSFR23638271	26817	30823065	322349	859977	32032208	9609662400
BERHUSFR23638272	3147	22098204	56125	123558	22281034	6684310200
BERHUSFR23638274	35869	32063083	541909	1569200	34210061	10263018300
BERHUSFR23638276	5494	23745385	29165	65812	23845856	7153756800
BERHUSFR23638278	44092	26168826	708795	2396065	29317778	8795333400
BERHUSFR23638280	5921	22950623	42842	47805	23047191	6914157300
BERHUSFR23638281	23082	29488270	301062	850403	30662817	9198845100
BERHUSFR23638284	72183	19233448	676119	2606727	22588477	6776543100
BERHUSFR23638286	360509	109882368	3281186	6719587	120243650	36073095000

BERHUSFR23638287B	20833	23833992	1120056	3126802	28101683	8430504900
BERHUSFR23638289	18273	20803671	100436	218535	21140915	6342274500
BERHUSFR23638290	36405	18937497	362339	1170108	20506349	6151904700
BERHUSFR23638292	42502	33538800	401795	1109239	35092336	10527700800
BERHUSFR23638294	42855	35487808	1275471	3433292	40239426	12071827800
BERHUSFR23638295	29289	16704089	48757	101373	16883508	5065052400
BERHUSFR23638298	11244	21874891	92630	230630	22209395	6662818500
BERHUSFR23638300	40154	15729446	345745	950381	17065726	5119717800
BERHUSFR23638304	5283	21755576	39437	53179	21853475	6556042500
BERHUSFR23638305	13788	18445192	129528	295151	18883659	5665097700
BERHUSFR23638306	54743	19020743	486302	1986794	21548582	6464574600
BERHUSFR23638308	3494	21849747	38370	25760	21917371	6575211300
BERHUSFR23638309	98104	53706630	210378	377545	54392657	16317797100
BERHUSFR23638310	254964	48684185	1688326	4086771	54714246	16414273800
BERHUSFR23638313	21836	22527165	358819	377512	23285332	6985599600
BERHUSFR23638314	17762	7986892	348179	802882	9155715	2746714500
BERHUSFR23638315	29256	34500529	677238	1647781	36854804	11056441200
BERHUSFR23638317	15872	20864236	394642	782809	22057559	6617267700
BERHUSFR23638318	16194	25419499	180191	231815	25847699	7754309700
BERHUSFR23638320	7892	22303749	11490	18531	22341662	6702498600
BERHUSFR23638321	101886	18858347	617760	2415672	21993665	6598099500
BERHUSFR23638322	16370	27855524	16114	12701	27900709	8370212700
BERHUSFR23638323	8110	21433140	29824	58005	21529079	6458723700
BERHUSFR23638324	41213	35125865	1443466	1811780	38422324	11526697200
BERHUSFR23638329	10630	26098280	153476	431916	26694302	8008290600
BERHUSFR23638332	20355	19690057	482592	1482455	21675459	6502637700
BERHUSFR23638335	64357	189005337	1031254	3103113	193204061	57961218300
BERHUSFR23638338	99011	25210360	784903	2516581	28610855	8583256500
BERHUSFR23638339	65768	21869065	582195	2009782	24526810	7358043000
BERHUSFR23638341	25151	36565936	156306	426588	37173981	11152194300
BERHUSFR23638342	40209	44630436	862750	1647761	47181156	14154346800
BERHUSFR23638343	22758	21424684	141758	253863	21843063	6552918900
BERHUSFR23638344	86680	18311410	883334	2472257	21753681	6526104300
BERHUSFR23638347	16227	22739549	36923	37090	22829789	6848936700
BERHUSFR23638348	10427	4952943	74300	229209	5266879	1580063700
BERHUSFR23638352	26516	15267379	437204	961242	16692341	5007702300
BERHUSFR23638354	12557	17381861	104656	283167	17782241	5334672300
BERHUSFR23638356	22045	23800075	139162	366869	24328151	7298445300

BERHUSFR23638358	223372	26311885	1627088	4511339	32673684	9802105200
BERHUSFR23638361	22964	58293897	183646	335419	58835926	17650777800
BERHUSFR23638376	37827	21677163	192796	552686	22460472	6738141600
BERHUSFR23638390	16523	18061587	117844	360189	18556143	5566842900
BERHUSFR23638392	34274	37321341	553025	1973562	39882202	11964660600
BERHUSFR23638415	23088	22101446	174704	621415	22920653	6876195900
BERHUSFR23638416	23923	21580638	210630	581069	22396260	6718878000
BERHUSFR23638436	41241	22128796	494637	1396461	24061135	7218340500
BERHUSFR23638439	148590	44753875	1330189	5071573	51304227	15391268100
BERHUSFR23638446	15353	37851281	93693	177932	38138259	11441477700
BERHUSFR23638447	29521	21783310	331478	696055	22840364	6852109200
BERHUSFR23638448	6058	23942220	99423	179736	24227437	7268231100
BERHUSFR23638840	46511	20089258	670553	1816758	22623080	6786924000
BERHUSFR23638858	19529	20457382	201126	820444	21498481	6449544300
BERHUSFR23638864	24846	15063750	295277	1061449	16445322	4933596600
BERHUSFR23638865	40733	41051472	715593	1409144	43216942	12965082600
BERHUSFR23638872	63652	23220230	495233	801935	24581050	7374315000
BERHUSFR23638881	34377	21624895	559185	1338185	23556642	7066992600
BERHUSFR23638882	28200	21408039	1124345	3028220	25588804	7676641200
BERHUSFR23638886	29958	21685417	75419	134914	21925708	6577712400
BERHUSFR23638887	37930	27810154	542824	1775269	30166177	9049853100
BERHUSFR23638889	34910	27115820	337448	957421	28445599	8533679700
BERHUSFR23638891	59567	19761752	549716	1844484	22215519	6664655700
BERHUSFR23638897B	9473	26686253	113140	305622	27114488	8134346400
BERHUSFR23638904	17836	12892778	39259	34852	12984725	3895417500
BERHUSFR23638906	85919	17487762	891817	4296523	22762021	6828606300
BERHUSFR23638910	12600	22565758	67498	257225	22903081	6870924300
BERHUSFR23638915	45788	15114826	449825	1473004	17083443	5125032900
BERHUSFR23638916	25062	21229470	155280	538140	21947952	6584385600
BERHUSFR23638918	20324	40155761	447069	1568157	42191311	12657393300
BERHUSFR23638921	57477	41208313	2022683	5119974	48408447	14522534100
BERHUSFR23638932	8180	21762800	203638	427497	22402115	6720634500
BERHUSFR23639014	74288	32010688	653706	1694438	34433120	10329936000
BERHUSFR23639036	13514	7651335	245947	383651	8294447	2488334100
BERHUSFR23639038	21798	20224888	463956	1329519	22040161	6612048300
BERHUSFR23639043	10284	21782325	52062	149138	21993809	6598142700
BERHUSFR23639056	83733	38568278	2781858	4970302	46404171	13921251300
BERHUSFR23639069	25681	28967209	214354	862378	30069622	9020886600

BERHUSFR23639116	27842	28433569	194038	359401	29014850	8704455000
BERHUSFR23639121	19190	21185504	306903	866137	22377734	6713320200
BERHUSFR23639124	11374	11395798	65711	120027	11592910	3477873000
BERHUSFR23639125	21265	30018930	250743	719433	31010371	9303111300
BERHUSFR23667932	16179	20669532	148335	181453	21015499	6304649700
BERHUSFR23668007	99615	55995600	543874	1037400	57676489	17302946700
BERHUSFR23668013B	10804	27840186	326307	750543	28927840	8678352000
BERHUSFR23668169	59144	27972589	298936	952702	29283371	8785011300
BERHUSFR23668184B	1579	22329280	23866	50117	22404842	6721452600
BERHUSFR23668185	123051	18807333	1707773	5873119	26511276	7953382800
BERHUSFR23668186	16732	26704896	175752	515182	27412562	8223768600
BERHUSFR23668189	63748	19739580	383911	1237394	21424633	6427389900
BERHUSFR23668190	22511	20151968	334465	828248	21337192	6401157600
BERHUSFR23668195	31012	23234923	488683	1508002	25262620	7578786000
BERHUSFR23668196	9930	11791890	239660	507878	12549358	3764807400
BERHUSFR23668199	43211	20203197	218372	305590	20770370	6231111000
BERHUSFR23668200	74380	20768294	1022677	3969489	25834840	7750452000
BERHUSFR23668203	30905	22415919	403474	1192161	24042459	7212737700
BERHUSFR23668207	21411	24990921	91838	195323	25299493	7589847900
BERHUSFR23668208	27133	29403398	250284	595237	30276052	9082815600
BERHUSFR23668211	16467	17912053	8010	7185	17943715	5383114500
BERHUSFR23668213	16376	23663748	34362	67745	23782231	7134669300
BERHUSFR23668214	38312	35514165	103844	282942	35939263	10781778900
BERHUSFR23668215	46175	14649981	163931	300948	15161035	4548310500
BERHUSFR23668304	12473	12967471	346571	951221	14277736	4283320800
BERHUSFR23668307B	3746	21769287	31650	48969	21853652	6556095600
BERHUSFR23668309	7899	7607438	55038	228361	7898736	2369620800
BERHUSFR23668311	8601	16058836	60098	94135	16221670	4866501000
BERHUSFR23668890	25578	21358136	232484	481948	22098146	6629443800
BERHUSFR23668893	58237	168241029	301676	736873	169337815	50801344500
BERHUSFR23668894	10307	23277229	227557	736663	24251756	7275526800
BERHUSFR23668896	90148	27811200	626071	3263481	31790900	9537270000
BERHUSFR23668899	17791	20393836	52041	57422	20521090	6156327000
BERHUSFR23668901	33150	22006004	382198	799774	23221126	6966337800
BERHUSFR23668902	26684	20312164	305030	769125	21413003	6423900900
BERHUSFR23668904	17065	24897080	127082	232854	25274081	7582224300
BERHUSFR23668908	39032	27519495	79232	115141	27752900	8325870000
BERHUSFR23668911	42685	24000242	252304	563623	24858854	7457656200

BERHUSFR23668913	44672	104829098	271849	679877	105825496	31747648800
BERHUSFR23668916	12061	19889332	22694	36991	19961078	5988323400
BERHUSFR23668917	27297	19570051	298427	722049	20617824	6185347200
BERHUSFR23668918	20679	21752307	248115	835348	22856449	6856934700
BERHUSFR23668920	46064	25066404	605477	1184652	26902597	8070779100
BERHUSFR23668925	13344	23831709	88228	286656	24219937	7265981100
BERHUSFR23668927	35057	24721238	84937	151019	24992251	7497675300
BERHUSFR23668929	33074	22007532	70867	190260	22301733	6690519900
BERHUSFR23668930	17883	23439036	69723	166275	23692917	7107875100
BERHUSFR23668931B	16216	21420576	185660	423732	22046184	6613855200
BERHUSFR23668932	11629	12915665	26143	42651	12996088	3898826400
BERHUSFR23668934	79433	27911942	440272	1361143	29792790	8937837000
BERHUSFR23668936	10728	4912765	91859	315838	5331190	1599357000
BERHUSFR23668939	27075	21425011	391238	691036	22534360	6760308000
BERHUSFR23668940	67036	16502176	709998	595286	17874496	5362348800
BERHUSFR23668944	20964	23857956	87738	252027	24218685	7265605500
BERHUSFR23668946	13279	25223288	120239	313340	25670146	7701043800
BERHUSFR23668947	64730	20241225	635937	1974921	22916813	6875043900
BERHUSFR23668948	344823	145887457	3478710	13764329	163475319	49042595700
BERHUSFR23668949	99843	39298861	479819	1106078	40984601	12295380300
BERHUSFR23668952	15514	19844721	90208	131032	20081475	6024442500
BERHUSFR23668953	15005	25127301	117208	332336	25591850	7677555000
BERHUSFR23668955	71632	53176272	904464	2106842	56259210	16877763000
BERHUSFR23668961	44926	47428303	243086	411956	48128271	14438481300
BERHUSFR23668962	8956	21186051	224452	466493	21885952	6565785600
BERHUSFR23668965	27683	21002806	295826	764024	22090339	6627101700
BERHUSFR23668967	44168	24991660	262550	608665	25907043	7772112900
BERHUSFR23668969	51790	21786825	409215	1181689	23429519	7028855700
BERHUSFR23668970	116947	38621066	517979	1657487	40913479	12274043700
BERHUSFR23668971	24024	21953220	181053	497956	22656253	6796875900
BERHUSFR23668972	23180	24274612	112257	329126	24739175	7421752500
BERHUSFR23668974	29319	20755444	737223	2153092	23675078	7102523400
BERHUSFR23668976	600471	65581302	6624867	19186587	91993227	27597968100
BERHUSFR23668980B	3634	24329376	63991	152807	24549808	7364942400
BERHUSFR23668981	16276	15083517	43404	126783	15269980	4580994000
BERHUSFR23668982	22711	27969848	134594	205139	28332292	8499687600
BERHUSFR23668985	10868	21689651	24726	33710	21758955	6527686500
BERHUSFR23668987	24232	42989754	131582	189143	43334711	13000413300

BERHUSFR23668992	15232	21397182	175533	313051	21900998	6570299400
BERHUSFR23668995	40837	23551383	999388	3130035	27721643	8316492900
BERHUSFR23668996	195819	25387784	1296660	4601749	31482012	9444603600
BERHUSFR23668997B	64227	26508951	923283	3288115	30784576	9235372800
BERHUSFR23669000	16578	20030752	405660	1123027	21576017	6472805100
BERHUSFR23669001	39447	35594284	764251	1706892	38104874	11431462200
BERHUSFR23669002	12173	21845378	135751	485064	22478366	6743509800
BERHUSFR23669003B	8354	21511224	155159	408262	22082999	6624899700
BERHUSFR23669004	13861	17743994	30216	62307	17850378	5355113400
BERHUSFR23669005	26009	31519819	158414	296688	32000930	9600279000
BERHUSFR23669006	14974	21635943	19107	25982	21696006	6508801800
BERHUSFR23669007	20155	45385126	466616	1129664	47001561	14100468300
BERHUSFR23669009	26162	18326235	1018205	2425315	21795917	6538775100
BERHUSFR23669010	37340	33805888	425508	969955	35238691	10571607300
BERHUSFR23669011	22478	21222859	231680	651921	22128938	6638681400
BERHUSFR23669014	4618	15873712	18635	24826	15921791	4776537300
BERHUSFR23669015	54021	19130457	485944	1949703	21620125	6486037500
BERHUSFR23669017	5593	13747076	98238	177021	14027928	4208378400
BERHUSFR23669018	22847	19961295	443855	1218842	21646839	6494051700
BERHUSFR23669019	6799	17221978	41750	100415	17370942	5211282600
BERHUSFR23669021	16991	21743595	73360	114977	21948923	6584676900
BERHUSFR23669023	98974	17655394	776118	3493080	22023566	6607069800
BERHUSFR23669024	438526	139371325	3405494	15443073	158658418	47597525400
BERHUSFR23669026	27248	21190738	124458	291907	21634351	6490305300
BERHUSFR23669028	22458	20738460	52189	52823	20865930	6259779000
BERHUSFR23669030	8267	21770614	22140	31515	21832536	6549760800
BERHUSFR23669031	44465	20944654	354473	1572436	22916028	6874808400
BERHUSFR23669032	79861	18433594	1066428	3028060	22607943	6782382900
BERHUSFR23669033	15841	25309045	573789	1186380	27085055	8125516500
BERHUSFR23669034	20029	21483580	55208	143269	21702086	6510625800
BERHUSFR23669035	13004	21884047	51471	112610	22061132	6618339600
BERHUSFR23669036	24022	39391983	37998	71267	39525270	11857581000
BERHUSFR23669037B	7870	21423704	265735	694566	22391875	6717562500
BERHUSFR23669038	26424	47403752	1125285	2733486	51288947	15386684100
BERHUSFR23669040	179820	13726758	1745316	6154725	21806619	6541985700
BERHUSFR23669042B	15225	20912066	159462	461607	21548360	6464508000
BERHUSFR23669043	19542	26355074	580881	1681918	28637415	8591224500
BERHUSFR23669044	45993	20487558	452657	1408264	22394472	6718341600

BERHUSFR23669047	32815	24213811	154207	586453	24987286	7496185800
BERHUSFR23669048	22072	46301551	245534	456119	47025276	14107582800
BERHUSFR23669049	50016	16487320	213799	284805	17035940	5110782000
BERHUSFR23669050	21636	12733204	246143	755618	13756601	4126980300
BERHUSFR23669052	39166	30857204	740814	2493196	34130380	10239114000
BERHUSFR23669055	25085	25247699	206636	357731	25837151	7751145300
BERHUSFR23669057	88399	44743900	422180	1037797	46292276	13887682800
BERHUSFR23669058	14505	7500782	21016	35076	7571379	2271413700
BERHUSFR23669059	19716	11607872	33998	77554	11739140	3521742000
BERHUSFR23669061B	5140	22841268	170494	543361	23560263	7068078900
BERHUSFR23669062	4557	4862974	23328	66838	4957697	1487309100
BERHUSFR23669063	15232	25757826	300021	694151	26767230	8030169000
BERHUSFR23669064	14379	26419605	109247	267127	26810358	8043107400
BERHUSFR23669065	17613	24699493	93921	281122	25092149	7527644700
BERHUSFR23669066	33802	21834374	210188	324266	22402630	6720789000
BERHUSFR23669067B	6458	20985687	92575	302610	21387330	6416199000
BERHUSFR23669069	11326	30070106	138112	336564	30556108	9166832400
BERHUSFR23669070	44905	22454252	288622	407661	23195440	6958632000
BERHUSFR23669076	52833	32024212	107605	156273	32340923	9702276900
BERHUSFR23669078	33319	23061861	272827	631827	23999834	7199950200
BERHUSFR23669087	13424	23690863	135871	414827	24254985	7276495500
BERHUSFR23669089	11748	21164303	58853	136330	21371234	6411370200
BERHUSFR23669095	110993	41614064	1044690	2816983	45586730	13676019000
BERHUSFR23669103	20799	19181606	207583	730845	20140833	6042249900
BERHUSFR23669111	48225	30875646	382128	556203	31862202	9558660600
BERHUSFR23669120B	1574	2333894	25471	83482	2444421	733326300
BERHUSFR23669121	29385	50630402	386923	789331	51836041	15550812300
BERHUSFR23669130	42872	36160421	197695	492552	36893540	11068062000
BERHUSFR23669137	1568016	849695918	15184109	34361708	900809751	270242925300
BERHUSFR23669138	9109	6586184	62672	171272	6829237	2048771100
BERHUSFR23669144	43993	20958369	214985	577137	21794484	6538345200
BERHUSFR23669147	9630	6098356	68179	138644	6314809	1894442700
BERHUSFR23669153	27915	35872482	53111	11986	35965494	10789648200
BERHUSFR23669154	48185	30548745	84973	120431	30802334	9240700200
BERHUSFR23669156	37145	88757543	241595	254620	89290903	26787270900
BERHUSFR23669157	17185	15771085	600065	1629752	18018087	5405426100
BERHUSFR23669163	10220	22560253	45763	45375	22661611	6798483300
BERHUSFR23669164	36061	28072617	717544	2285454	31111676	9333502800

BERHUSFR23669177	45573	25955636	473647	1623644	28098500	8429550000
BERHUSFR23669181	22505	8653683	306174	998043	9980405	2994121500
BERHUSFR23669190	18218	12837160	126886	367471	13349735	4004920500
BERHUSFR23669252	29815	21080354	292091	564383	21966643	6589992900
BERHUSFR23669261	4363	4654814	29507	36200	4724884	1417465200
BERHUSFR23669262	358049	97132167	4285797	9003583	110779596	33233878800
BERHUSFR23669270	55084	21266418	346229	1076511	22744242	6823272600
BERHUSFR23669368	38128	22645011	146606	318800	23148545	6944563500
BERHUSFR23669372	10658	37886215	25863	20004	37942740	11382822000
BERHUSFR23669375	107042	36232690	409057	884075	37632864	11289859200
BERHUSFR23669379	21427	22646038	118846	220525	23006836	6902050800
BERHUSFR23669424	54939	22392179	852523	2007948	25307589	7592276700
BERHUSFR23669427	10968	24293923	128980	295028	24728899	7418669700
BERHUSFR23669430	36889	19692197	250604	375320	20355010	6106503000
BERHUSFR23669452	26571	10714821	112299	316215	11169906	3350971800
BERHUSFR23669494	84355	33776192	348323	930833	35139703	10541910900
BERHUSFR23669497	94315	21541226	564261	1934404	24134206	7240261800
BERHUSFR23669509	38352	23653949	219871	764599	24676771	7403031300
BERHUSFR23669527	5664	15606055	15280	5975	15632974	4689892200
BERHUSFR23669533	9784	19451757	23422	24336	19509299	5852789700
BERHUSFR23669538	18075	25602192	54367	99852	25774486	7732345800
BERHUSFR23669551	16103	24124233	142332	321094	24603762	7381128600
BERHUSFR23669553	222256	150847524	3650139	8812361	163532280	49059684000
BERHUSFR23669558B	14560	26140008	134859	344368	26633795	7990138500
BERHUSFR23669560	9545	21136779	180398	414564	21741286	6522385800
BERHUSFR23669561	33132	36468365	417943	1361821	38281261	11484378300
BERHUSFR23669562B	6035	23522277	70250	216861	23815423	7144626900
BERHUSFR23669563	31614	24089710	21444	22039	24164807	7249442100
BERHUSFR23669565	33145	23409387	367927	1806559	25617018	7685105400
BERHUSFR23669566	42457	49499079	627617	1557708	51726861	15518058300
BERHUSFR23669568	21314	21822382	232453	760998	22837147	6851144100
BERHUSFR23669569	41585	20959222	252665	635858	21889330	6566799000
BERHUSFR23669571	5376	23897028	33327	92751	24028482	7208544600
BERHUSFR23669574	113458	18844865	719262	3049097	22726682	6818004600
BERHUSFR23669576	14450	20352670	274540	638294	21279954	6383986200
BERHUSFR23669578	14932	20603216	254658	514402	21387208	6416162400
BERHUSFR23669580	62588	23870209	437486	1017428	25387711	7616313300
BERHUSFR23669582	19603	30484847	561556	2163620	33229626	9968887800

BERHUSFR23669585	27132	23943507	423230	1041325	25435194	7630558200
BERHUSFR23669587	23225	33093992	283944	1052248	34453409	10336022700
BERHUSFR23669589	19503	24717414	220942	969932	25927791	7778337300
BERHUSFR23669591	4321	21263360	36322	70039	21374042	6412212600
BERHUSFR23669592	44779	20826016	651089	1994466	23516350	7054905000
BERHUSFR23669593	19407	42339743	216559	669859	43245568	12973670400
BERHUSFR23669595	39720	103776807	634730	1942244	106393501	31918050300
BERHUSFR23669597	18552	24582615	164355	572502	25338024	7601407200
BERHUSFR23669598	31678	42656924	162047	276357	43127006	12938101800
BERHUSFR23669601	20097	21029152	273762	402633	21725644	6517693200
BERHUSFR23669602	27124	24215227	324735	775020	25342106	7602631800
BERHUSFR23669605	44266	22729548	227207	805343	23806364	7141909200
BERHUSFR23669606	6551	16549998	327656	568630	17452835	5235850500
BERHUSFR23669611	19371	23325298	85372	186091	23616132	7084839600
BERHUSFR23669612	81470	18554722	794967	3246461	22677620	6803286000
BERHUSFR23669614	20591	23197652	195235	462270	23875748	7162724400
BERHUSFR23669619	97491	35827968	1082133	2445185	39452777	11835833100
BERHUSFR23669620	25849	25263431	145054	182872	25617206	7685161800
BERHUSFR23669626	19761	21230242	281148	858522	22389673	6716901900
BERHUSFR23669629	12556	21093623	34255	59659	21200093	6360027900
BERHUSFR23669631	43727	32161683	341030	839935	33386375	10015912500
BERHUSFR23669632	10432	6989072	81110	244938	7325552	2197665600
BERHUSFR23669634	7697	24198694	37739	90584	24334714	7300414200
BERHUSFR23669640	31279	25182481	305975	748913	26268648	7880594400
BERHUSFR23669641	9944	22766076	31601	82170	22889791	6866937300
BERHUSFR23669643	19493	21168990	1309922	3181503	25679908	7703972400
BERHUSFR23669645	134698	19081569	1102555	3369641	23688463	7106538900
BERHUSFR23669647	46493	23144389	248298	685970	24125150	7237545000
BERHUSFR23669648	15843	23242902	316509	1123225	24698479	7409543700
BERHUSFR23669652	21674	22985194	193406	517851	23718125	7115437500
BERHUSFR23669654	23263	29526091	349005	744248	30642607	9192782100
BERHUSFR23669661	51356	29395421	290350	675237	30412364	9123709200
BERHUSFR23669673	81031	157417833	742376	1311693	159552933	47865879900
BERHUSFR23669677	98477	18862779	957747	2495964	22414967	6724490100
BERHUSFR23669678	14870	5883745	34836	100832	6034283	1810284900
BERHUSFR23669694	19478	20653514	271704	501840	21446536	6433960800
BERHUSFR23669697	101487	39620134	1157576	2597712	43476909	13043072700
BERHUSFR23669707	13524	21512962	110213	182653	21819352	6545805600

BERHUSFR23669710	21304	21484162	98243	289877	21893586	6568075800
BERHUSFR23669711	315226	206529110	2628732	7098861	216571929	64971578700
BERHUSFR23669720	5799	12249939	24223	43010	12322971	3696891300
BERHUSFR23669721	497724	460927443	1043221	1091834	463560222	139068066600
BERHUSFR23669744	110132	19502285	636519	1781861	22030797	6609239100
BERHUSFR23669747	12147	23092739	87293	328930	23521109	7056332700
BERHUSFR23669752	11757	12927035	84794	134666	13158252	3947475600
BERHUSFR23669781	22786	16888640	150119	525778	17587323	5276196900
BERHUSFR23669792	16717	19719187	110959	201686	20048549	6014564700
BERHUSFR23669794	42313	28657172	328710	799216	29827411	8948223300
BERHUSFR23669800	113272	22543395	757203	3344215	26758085	8027425500
BERHUSFR23669803	67942	20882336	350079	1023471	22323828	6697148400
BERHUSFR23669805	120793	130065274	1712718	2867465	134766250	40429875000
BERHUSFR23669814	866225	195841373	7845817	21752411	226305826	67891747800
BERHUSFR23669840	108800	60950346	823023	2309986	64192155	19257646500
BERHUSFR23669842B	9129	21818938	98314	291130	22217511	6665253300
BERHUSFR23670375B	7208	21273115	138836	411003	21830162	6549048600
BERHUSFR23670424	54496	27772546	474909	1500252	29802203	8940660900
BERHUSFR23670426	45944	29084779	325872	861238	30317833	9095349900
BERHUSFR23670427	54495	22393204	601629	1674973	24724301	7417290300
BERHUSFR23670429	24858	22643932	339151	772541	23780482	7134144600
BERHUSFR23670431	36146	33042266	217632	600374	33896418	10168925400
BERHUSFR23670435	49151	25450541	515757	1353027	27368476	8210542800
BERHUSFR23670438	56474	25437658	754331	2964201	29212664	8763799200
BERHUSFR23670442	23540	42689329	73091	184412	42970372	12891111600
BERHUSFR23670444	55690	36092360	627346	2094464	38869860	11660958000
BERHUSFR23670448	62119	28017306	559612	1571835	30210872	9063261600
BERHUSFR23670449	89787	26217715	577416	1211857	28096775	8429032500
BERHUSFR23670454	59063	21855319	628796	1693321	24236499	7270949700
BERHUSFR23670459	19204	21591129	105644	208208	21924185	6577255500
BERHUSFR23670462	34711	28496737	690035	1447158	30668641	9200592300
BERHUSFR23670465	49315	22246207	129774	357431	22782727	6834818100
BERHUSFR23670466	34154	31477389	159742	257598	31928883	9578664900
BERHUSFR23670467	7945	27763367	89495	191712	28052519	8415755700
BERHUSFR23670468B	6156	21384267	208948	461845	22061216	6618364800
BERHUSFR23670470	36921	21407715	464199	1402958	23311793	6993537900
BERHUSFR23670472	57618	23840243	198704	341276	24437841	7331352300
BERHUSFR23670474	52070	19811018	851752	2514901	23229741	6968922300

BERHUSFR23670477	19159	22648662	169335	574623	23411779	7023533700
BERHUSFR23670478	8930	32758511	76939	103638	32948018	9884405400
BERHUSFR23670480	1685148	252058496	12781242	43746379	310271265	93081379500
BERHUSFR23670482	11275	24607124	102646	292381	25013426	7504027800
BERHUSFR23670484	49814	47564796	69231	158620	47842461	14352738300
BERHUSFR23670486B	9607	26485761	152278	621960	27269606	8180881800
BERHUSFR23670488	5103	21474901	23226	45713	21548943	6464682900
BERHUSFR23670491	43598	20473752	541257	1473304	22531911	6759573300
BERHUSFR23670495	34411	26237575	202900	732596	27207482	8162244600
BERHUSFR23670496	6823	21212530	44627	182944	21446924	6434077200
BERHUSFR23670497	10538	21625863	102157	302620	22041178	6612353400
BERHUSFR23670498B	6796	24025185	98460	415043	24545484	7363645200
BERHUSFR23670504	51312	30558039	425031	1206312	32240694	9672208200
BERHUSFR23670505	16885	22523349	223420	441622	23205276	6961582800
BERHUSFR23670506	26359	31539769	267118	1019657	32852903	9855870900
BERHUSFR23670509	172013	199108798	1800602	4531093	205612506	61683751800
BERHUSFR23670636	120149	28401399	1008371	2233993	31763912	9529173600
BERHUSFR23670663	31306	24384022	409530	1417670	26242528	7872758400
BERHUSFR23670708	19232	20698959	188965	539797	21446953	6434085900
BERHUSFR25324409	24797	21369267	239504	705083	22338651	6701595300
BERHUSFR25324413	15940	34923888	157894	261706	35359428	10607828400
BERHUSFR25324416	53904	21130839	448587	1597059	23230389	6969116700
BERHUSFR25324436	12396	26224903	27042	43808	26308149	7892444700
BERHUSFR25324440	11380	13466755	53933	118169	13650237	4095071100
BERHUSFR25324441	84631	27670213	562333	1744938	30062115	9018634500
BERHUSFR25324447	9632	12366746	50108	124051	12550537	3765161100
BERHUSFR25324450	234506	42992878	2312641	6012947	51552972	15465891600
BERHUSFR25324457	10437	21797264	119920	540870	22468491	6740547300
BERHUSFR25324460	17971	16903337	229220	553486	17704014	5311204200
BERHUSFR25324464	75104	17935994	926652	2953827	21891577	6567473100
BERHUSFR25324466	36895	20298633	421445	2061210	22818183	6845454900
BERHUSFR25324480B	10617	22610424	20083	20913	22662037	6798611100
BERHUSFR25324481	18736	7356505	386033	1090538	8851812	2655543600
BERHUSFR25324490	107123	29097152	782353	1979185	31965813	9589743900
BERHUSFR25324493	13062	17728092	68598	120817	17930569	5379170700
BERHUSFR25324498B	3429	20662477	232103	698309	21596318	6478895400
BERHUSFR25324504	67570	15821798	492108	2279136	18660612	5598183600
BERHUSFR25324506	24651	28497733	244747	604627	29371758	8811527400

BERHUSFR25324522	26776	64748808	515670	1391109	66682363	20004708900
BERHUSFR25324534	18967	24021201	79497	147717	24267382	7280214600
BERHUSFR25324556	11458	26271988	427396	1525595	28236437	8470931100
BERHUSFR25324563	44316	49794348	411492	1104888	51355044	15406513200
BERHUSFR25324568	14671	20803724	126105	446412	21390912	6417273600
BERHUSFR25324575	26450	21396717	380898	1131668	22935733	6880719900
BERHUSFR25324576	7539	10427792	151668	380087	10967086	3290125800
BERHUSFR25324591	21572	15883870	160817	404872	16471131	4941339300
BERHUSFR25324592	23278	18394343	690434	1655106	20763161	6228948300
BERHUSFR25324595	26658	22354404	202291	685676	23269029	6980708700
BERHUSFR25324596	37605	11752097	294498	817282	12901482	3870444600
BERHUSFR25324601	10923	25703871	70022	195670	25980486	7794145800
BERHUSFR25324606	53808	13087044	928860	2761654	16831366	5049409800
BERHUSFR25324610	26965	30708243	424989	1037716	32197913	9659373900
BERHUSFR25324612	69952	36330034	708059	1898849	39006894	11702068200
BERHUSFR25324619	136004	29175102	710952	2499534	32521592	9756477600
BERHUSFR25324622	41154	33686774	287075	672999	34688002	10406400600
BERHUSFR25324634	72012	44671201	554178	1311780	46609171	13982751300
BERHUSFR25324638	5805	8803480	173031	412202	9394518	2818355400
BERHUSFR25324639	72211	18641436	904503	1734269	21352419	6405725700
BERHUSFR25324641	7630	10000645	34625	70279	10113179	3033953700
BERHUSFR25324642	29310	12525639	479379	1101768	14136096	4240828800
BERHUSFR25324646	39268	20714984	290741	1015559	22060552	6618165600
BERHUSFR25324648	145879	16479892	1715888	4808106	23149765	6944929500
BERHUSFR25324649	44183	15712330	621117	1994446	18372076	5511622800
BERHUSFR25324652	31701	11410853	54184	165840	11662578	3498773400
BERHUSFR25324658B	7198	21477251	64633	199111	21748193	6524457900
BERHUSFR25324666	9827	21610954	75072	219397	21915250	6574575000
BERHUSFR25324679	5845	9784299	26961	56178	9873283	2961984900
BERHUSFR25324683	18539	19468894	110403	330828	19928664	5978599200
BERHUSFR25324808	42905	17217237	275515	907033	18442690	5532807000
BERHUSFR25324850	644869	1139172177	10128859	15578637	1165524542	349657362600
BERHUSFR25324856	10994	16567703	83217	233216	16895130	5068539000
BERHUSFR25324865	3565	10567289	27427	64241	10662522	3198756600
BERHUSFR25324881	7989	27598264	120716	217400	27944369	8383310700
BERHUSFR25324882	61248	16058924	643977	1435076	18199225	5459767500
BERHUSFR25324886B	57487	27981575	533627	1810591	30383280	9114984000
BERHUSFR25324889	61472	19905001	608190	1665010	22239673	6671901900

BERHUSFR25324893	34453	15823106	531091	2098748	18487398	5546219400
BERHUSFR25324894	22084	9541045	580941	1378921	11522991	3456897300
BERHUSFR25324895	15624	19161288	52030	129822	19358764	5807629200
BERHUSFR25324897	49597	20646815	389483	1106999	22192894	6657868200
BERHUSFR25324900	26483	21318824	195441	454038	21994786	6598435800
BERHUSFR25324902	367286	33658881	3237908	11222072	48486147	14545844100
BERHUSFR25324903	3131	7119170	7797	14628	7144726	2143417800
BERHUSFR25324904	6328	22970412	48494	89990	23115224	6934567200
BERHUSFR25324905	36112	39979973	549255	1591908	42157248	12647174400
BERHUSFR25324906	27976	32610903	144074	400697	33183650	9955095000
BERHUSFR25324907	38682	32047031	185754	707187	32978654	9893596200
BERHUSFR25324909	7348	23051696	44009	144105	23247158	6974147400
BERHUSFR25324910	30634	12794381	177586	582846	13585447	4075634100
BERHUSFR25324917	38951	19556461	371005	1489894	21456311	6436893300
BERHUSFR25324919	65280	16439088	402582	1292677	18199627	5459888100
BERHUSFR25324920	53428	17676324	578616	1553858	19862226	5958667800
BERHUSFR25324923	29742	24819825	224990	504740	25579297	7673789100
BERHUSFR25324924	4438	11591841	14019	11254	11621552	3486465600
BERHUSFR25324925	17069	20335299	382581	1013155	21748104	6524431200
BERHUSFR25324931	72074	19882594	524057	1560943	22039668	6611900400
BERHUSFR25324932	13143	29897958	173303	410600	30495004	9148501200
BERHUSFR25324933	54603	18984347	1031803	3438965	23509718	7052915400
BERHUSFR25324936	24485	26131146	223332	640819	27019782	8105934600
BERHUSFR25324937	19289	23414155	104154	269412	23807010	7142103000
BERHUSFR25324938	57258	16014533	501628	1972582	18546001	5563800300
BERHUSFR25324943	31376	20968948	183649	443989	21627962	6488388600
BERHUSFR25324945	19492	19217413	106586	279966	19623457	5887037100
BERHUSFR25324946	6253	21581102	58223	112316	21757894	6527368200
BERHUSFR25324948	8805	38659378	22914	20117	38711214	11613364200
BERHUSFR25324950	73124	23513950	510360	1575094	25672528	7701758400
BERHUSFR25324952	12548	24613665	140909	493829	25260951	7578285300
BERHUSFR25324953	23809	23248039	244763	653419	24170030	7251009000
BERHUSFR25324956	15408	28779573	201112	487653	29483746	8845123800
BERHUSFR25324957	6185	21275193	93078	136535	21510991	6453297300
BERHUSFR25324959	65368	21266337	725517	2311364	24368586	7310575800
BERHUSFR25324960	9620	23658733	71560	149440	23889353	7166805900
BERHUSFR25324961	20812	21324992	330507	815936	22492247	6747674100
BERHUSFR25324962	177396	83607829	892819	2058490	86736534	26020960200

BERHUSFR25324963	5841	9313464	99813	282878	9701996	2910598800
BERHUSFR25324964	48596	60016845	318646	711178	61095265	18328579500
BERHUSFR25324967	22615	20915049	455396	1114539	22507599	6752279700
BERHUSFR25324968	7374	35252049	26404	16007	35301834	10590550200
BERHUSFR25324969	8763	22581679	112688	179618	22882748	6864824400
BERHUSFR25324970	4135	21644336	30131	40485	21719087	6515726100
BERHUSFR25324971	142438	182000992	1151084	1515693	184810207	55443062100
BERHUSFR25324972	18257	21933717	96386	154585	22202945	6660883500
BERHUSFR25324973	28244	16252108	265146	492902	17038400	5111520000
BERHUSFR25324974	11494	21070806	151495	239463	21473258	6441977400
BERHUSFR25324976	12962	22172547	131883	288441	22605833	6781749900
BERHUSFR25324981	117359	142604219	421134	567764	143710476	43113142800
BERHUSFR25324983	12939	12925631	73203	219073	13230846	3969253800
BERHUSFR25324989	69640	18525166	622956	2524231	21741993	6522597900
BERHUSFR25324995	39559	33954133	248190	1067156	35309038	10592711400
BERHUSFR25324996	17060	20732662	314390	794497	21858609	6557582700
BERHUSFR25324997	153806	29317763	1061931	2526143	33059643	9917892900
BERHUSFR25325000	39586	13217023	314639	899246	14470494	4341148200
BERHUSFR25325003	39626	19245225	608334	1543831	21437016	6431104800
BERHUSFR25325004	38515	19951100	649288	1816247	22455150	6736545000
BERHUSFR25325009	508330	972221922	7291122	12718837	992740211	297822063300
BERHUSFR25325011	28305	36913790	182428	537784	37662307	11298692100
BERHUSFR25325012	16856	12371730	155422	378630	12922638	3876791400
BERHUSFR25325014	3626	21816433	23311	28926	21872296	6561688800
BERHUSFR25325026	10581	34935326	89460	202535	35237902	10571370600
BERHUSFR25325027	4783	23089677	28283	66074	23188817	6956645100
BERHUSFR25325028	111024	10642257	1144311	3759779	15657371	4697211300
BERHUSFR25325030	46898	31298979	391763	898175	32635815	9790744500
BERHUSFR25325032	4607	10692750	10778	10369	10718504	3215551200
BERHUSFR25325035	41470	22950992	95011	162258	23249731	6974919300
BERHUSFR25325036	25026	22478647	77359	117854	22698886	6809665800
BERHUSFR25325038	77664	22744550	453508	1597368	24873090	7461927000
BERHUSFR25325040B	3484	24880205	18066	22751	24924506	7477351800
BERHUSFR25325041	43477	18040480	198243	272633	18554833	5566449900
BERHUSFR25325042	20162	21391714	351286	1281468	23044630	6913389000
BERHUSFR25325053	55605	31131991	531702	2789817	34509115	10352734500
BERHUSFR25325062	12787	22702722	179604	499368	23394481	7018344300
BERHUSFR25325069	35927	26247517	185532	594957	27063933	8119179900

BERHUSFR25325070	6514	16889390	44819	98448	17039171	5111751300
BERHUSFR25325073	22707	23817311	160107	630248	24630373	7389111900
BERHUSFR25325083	282116	75467255	1939942	8271118	85960431	25788129300
BERHUSFR25325094	48082	43112764	678363	2061871	45901080	13770324000
BERHUSFR25325096	14355	23913069	140952	465589	24533965	7360189500
BERHUSFR25325105	169209	15171756	664413	2286073	18291451	5487435300
BERHUSFR25325113	9201	22937137	172455	333089	23451882	7035564600
BERHUSFR25325150	41360	22957490	397469	1467778	24864097	7459229100
BERHUSFR25325173	8460	21707586	73264	71520	21860830	6558249000
BERHUSFR25325178	73603	21151679	634527	2167624	24027433	7208229900
BERHUSFR25325180	21038	21172239	220350	719431	22133058	6639917400
BERHUSFR25325181B	16537	24961701	242929	886295	26107462	7832238600
BERHUSFR25325182	58852	27040254	786625	2292384	30178115	9053434500
BERHUSFR25325189	9274	43548329	86476	282119	43926198	13177859400
BERHUSFR25325191	6464	22224960	56602	75339	22363365	6709009500
BERHUSFR25325192	73043	70268768	583153	1694068	72619032	21785709600
BERHUSFR25325197	29467	24336749	193323	1046764	25606303	7681890900
BERHUSFR25325200	23952	31416206	252081	809081	32501320	9750396000
BERHUSFR25325218	19896	33237725	361570	1256038	34875229	10462568700
BERHUSFR25325234	6300	34811531	76250	92157	34986238	10495871400
BERHUSFR25325237	49168	52702815	299895	651315	53703193	16110957900
BERHUSFR25325239	43563	21959410	447339	1468782	23919094	7175728200
BERHUSFR25325251	15225	22598032	346200	884221	23843678	7153103400
BERHUSFR25325252	22180	21263383	150873	386537	21822973	6546891900
BERHUSFR25325257	661297	391249606	6724959	13030060	411665922	123499776600
BERHUSFR25325263	64652	20353617	453003	1615705	22486977	6746093100
BERHUSFR25325269	34562	22268823	326651	1142831	23772867	7131860100
BERHUSFR25325271	14080	19629208	146308	237973	20027569	6008270700
BERHUSFR25325272	12051	22276125	59814	134922	22482912	6744873600
BERHUSFR25325274	62618	32578864	1500047	3869834	38011363	11403408900
BERHUSFR25325275	74800	36504681	508866	1983678	39072025	11721607500
BERHUSFR25325276	42610	31167472	655010	3016754	34881846	10464553800
BERHUSFR25325279	5249	23038691	89649	164501	23298090	6989427000
BERHUSFR25325281	14044	15477348	230026	678670	16400088	4920026400
BERHUSFR25325282	7140	21741044	72894	133291	21954369	6586310700
BERHUSFR25325283	17105	71953218	231986	334900	72537209	21761162700
BERHUSFR25325284	23195	24094031	130657	295518	24543401	7363020300
BERHUSFR25325286	12112	28786288	15231	11372	28825003	8647500900

BERHUSFR25325287	7408	21461591	42318	51274	21562591	6468777300
BERHUSFR25325288	17442	7695365	360587	1097773	9171167	2751350100
BERHUSFR25325289	4344	22285043	29873	76271	22395531	6718659300
BERHUSFR25325290	21364	36148306	445235	909049	37523954	11257186200
BERHUSFR25325294	18692	33157335	275241	681881	34133149	10239944700
BERHUSFR25325295	8922	22603008	31715	51402	22695047	6808514100
BERHUSFR25325297	12814	33717727	167203	482220	34379964	10313989200
BERHUSFR25325298	31545	24280055	389987	1018496	25720083	7716024900
BERHUSFR25325300	15184	43233311	88359	150988	43487842	13046352600
BERHUSFR25325302	34150	73086349	1074719	2825261	77020479	23106143700
BERHUSFR25325304	17186	22101843	134636	351608	22605273	6781581900
BERHUSFR25325306	9699	22684686	82644	145412	22922441	6876732300
BERHUSFR25325307	15639	32937003	209648	666631	33828921	10148676300
BERHUSFR25325309	29744	21682284	137893	330720	22180641	6654192300
BERHUSFR25325312B	3926	21768883	62710	108100	21943619	6583085700
BERHUSFR25325313	83907	29022687	937000	3173926	33217520	9965256000
BERHUSFR25325314B	17846	22318696	308026	913762	23558330	7067499000
BERHUSFR25325315	16349	23390300	222595	415997	24045241	7213572300
BERHUSFR25325316	9512	35390186	80830	158575	35639103	10691730900
BERHUSFR25325317	20341	25249737	74598	179038	25523714	7657114200
BERHUSFR25325318	9696	30720544	45208	23991	30799439	9239831700
BERHUSFR25325320	21437	28609032	151205	208116	28989790	8696937000
BERHUSFR25325323	54261	17216900	1103957	3034038	21409156	6422746800
BERHUSFR25325324	14096	34334501	555389	1381687	36285673	10885701900
BERHUSFR25325327	13235	35352379	137780	393672	35897066	10769119800
BERHUSFR25325328	10741	23924365	76362	117677	24129145	7238743500
BERHUSFR25325329	25463	39690378	300355	1045298	41061494	12318448200
BERHUSFR25325331B	18038	32833074	323766	1005810	34180688	10254206400
BERHUSFR25325332	12059	21213849	55000	71317	21352225	6405667500
BERHUSFR25325333	20125	54085764	333577	909757	55349223	16604766900
BERHUSFR25325334	53224	21939960	312755	1378290	23684229	7105268700
BERHUSFR25325336	14901	24701059	164027	538944	25418931	7625679300
BERHUSFR25325337	34895	18492757	1866796	3473348	23867796	7160338800
BERHUSFR25325338	24979	35486081	621146	1784403	37916609	11374982700
BERHUSFR25325340	34001	23392724	138417	418710	23983852	7195155600
BERHUSFR25325341	3598	38928706	31402	8189	38971895	11691568500
BERHUSFR25325342	15299	19848065	457207	937661	21258232	6377469600
BERHUSFR25325343	15353	24686462	148201	600767	25450783	7635234900

BERHUSFR25325344	12474	22805035	148751	324128	23290388	6987116400
BERHUSFR25325348	47076	23509485	375919	1030389	24962869	7488860700
BERHUSFR25325349	24354	20754903	202714	579899	21561870	6468561000
BERHUSFR25325351	31094	51701951	129698	324033	52186776	15656032800
BERHUSFR25325353	17190	21133991	85051	358601	21594833	6478449900
BERHUSFR25325356	96831	56709321	1161073	2123008	60090233	18027069900
BERHUSFR25325359	95037	34512391	587280	1486447	36681155	11004346500

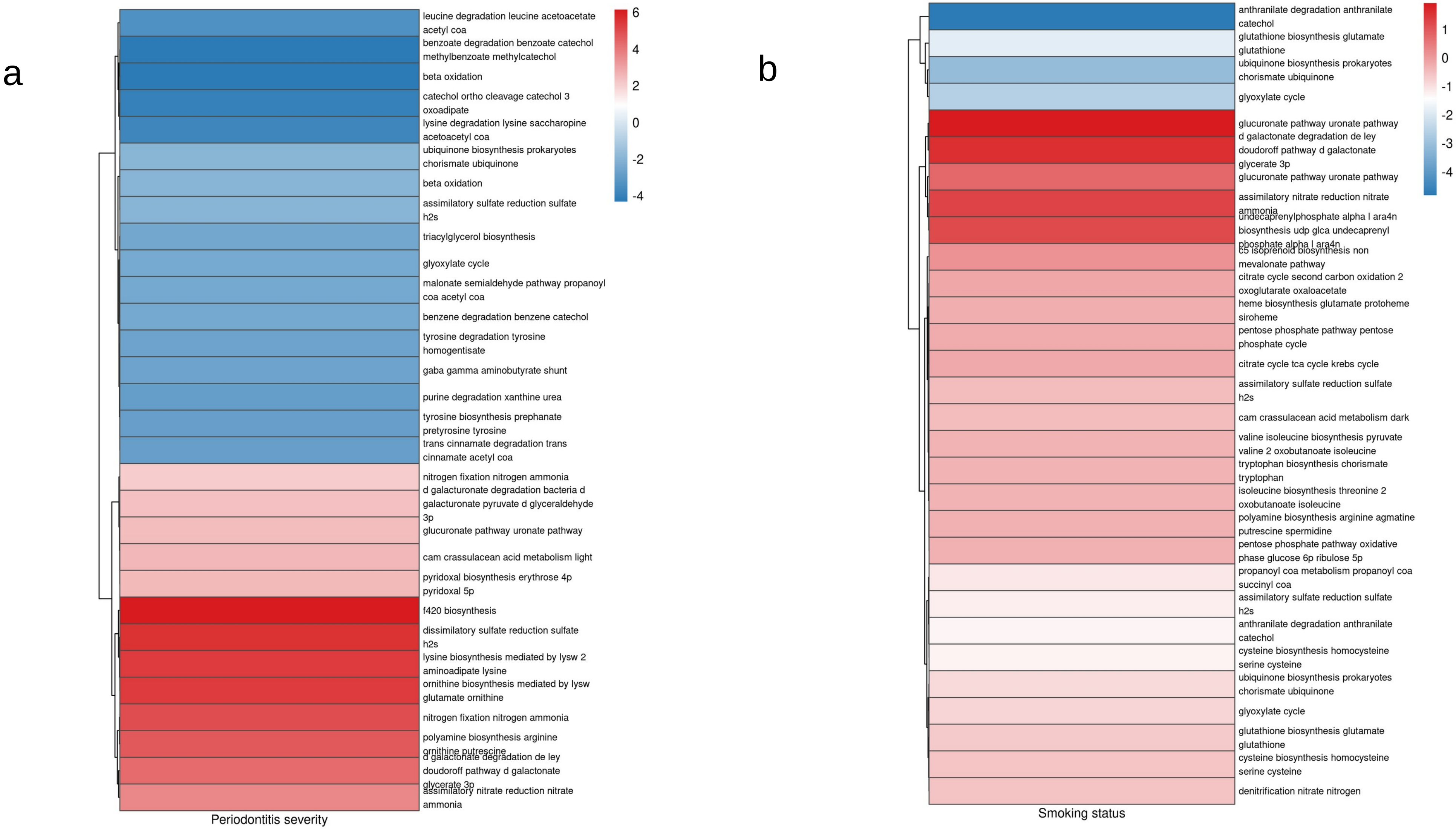

Supplementary Figure S1

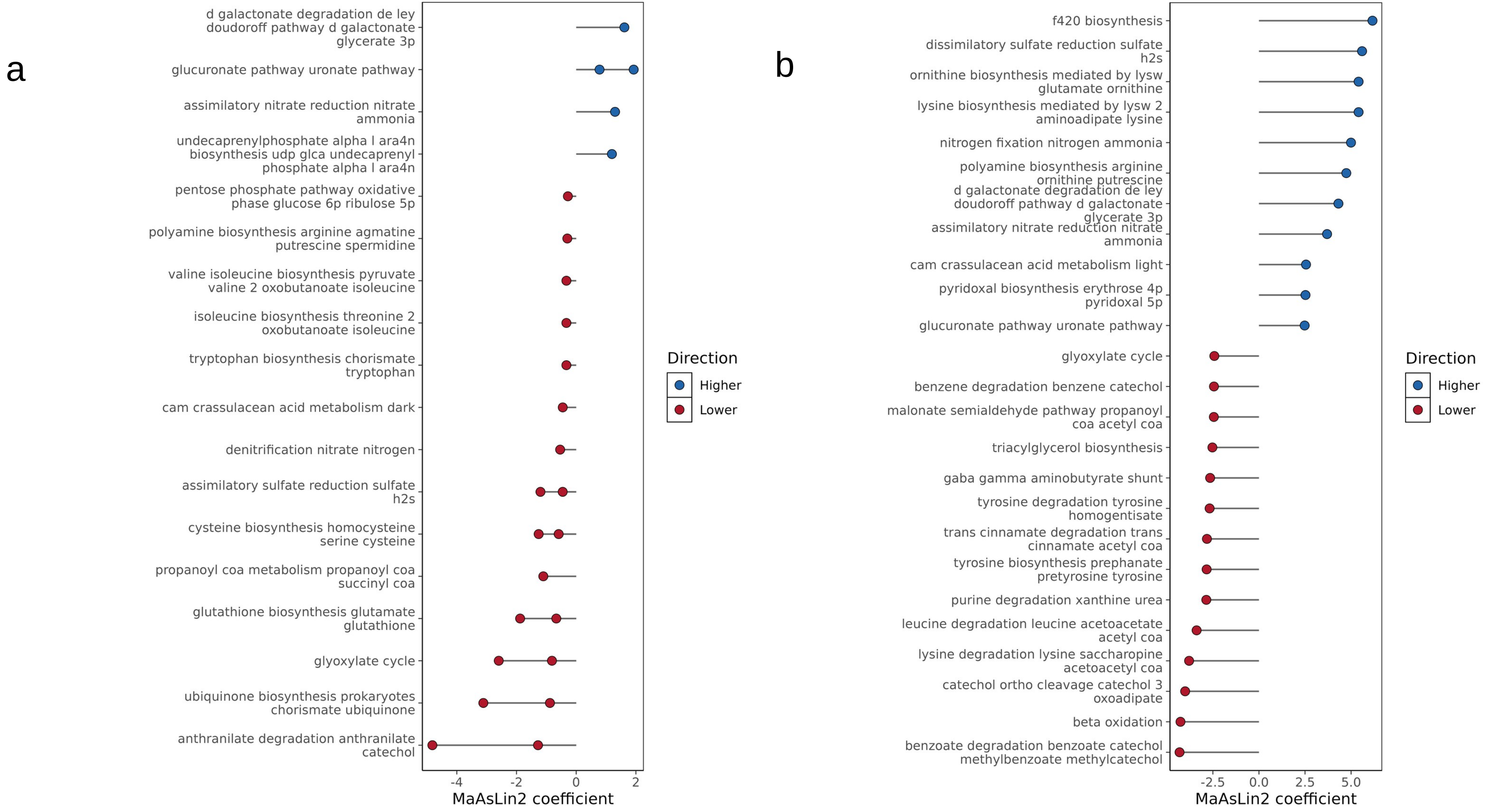

Supplementary Figure S2
